# Prevalence and outcomes of frailty in unplanned hospital admissions: a systematic review and meta-analysis of hospital-wide and general (internal) medicine cohorts

**DOI:** 10.1016/j.eclinm.2023.101947

**Published:** 2023-04-21

**Authors:** Emily L. Boucher, Jasmine M. Gan, Peter M. Rothwell, Sasha Shepperd, Sarah T. Pendlebury

**Affiliations:** a Wolfson Centre for Prevention of Stroke and Dementia, Wolfson Building, Nuffield Department of Clinical Neurosciences, University of Oxford, UK; b Nuffield Department of Population Health, University of Oxford, UK; c NIHR Oxford Biomedical Research Centre and Departments of Acute General (Internal) Medicine and Geratology, Oxford University Hospitals NHS Foundation Trust, UK

**Keywords:** Frailty, Older adults, Hospitals, General (internal) medicine, Mortality, Length of stay, Readmission, Discharge home

## Abstract

**Background:**

Guidelines recommend routine frailty screening for all hospitalised older adults to inform care decisions, based mainly on studies in elective or speciality-specific settings. However, most hospital bed days are accounted for by acute non-elective admissions, in which the prevalence and prognostic value of frailty might differ, and uptake of screening is limited. We therefore did a systematic review and meta-analysis of frailty prevalence and outcomes in unplanned hospital admissions.

**Methods:**

We searched MEDLINE, EMBASE and CINAHL up to 31/01/2023 and included observational studies using validated frailty measures in adult hospital-wide or general medicine admissions. Summary data on the prevalence of frailty and associated outcomes, measurement tools, study setting (hospital-wide vs general medicine), and design (prospective vs retrospective) were extracted and risk of bias assessed (modified Joanna Briggs Institute checklists). Unadjusted relative risks (RR; moderate/severe frailty vs no/mild) for mortality (within one year), length of stay (LOS), discharge destination and readmission were calculated and pooled, where appropriate, using random-effects models. PROSPERO CRD42021235663.

**Findings:**

Among 45 cohorts (median/SD age = 80/5 years; n = 39,041,266 admissions, n = 22 measurement tools) moderate/severe frailty ranged from 14.3% to 79.6% overall (and in the 26 cohorts with low-moderate risk of bias) with considerable heterogeneity between studies (p_het_ < 0.001) preventing pooling of results but with rates <25% in only 3 cohorts. Moderate/severe vs no/mild frailty was associated with increased mortality (n = 19 cohorts; RR range = 1.08–3.70), more consistently among cohorts using clinically administered tools (n = 11; RR range = 1.63–3.70; p_het_ = 0.08; pooled RR = 2.53, 95% CI = 2.15–2.97) vs cohorts using (retrospective) administrative coding data (n = 8; RR range = 1.08–3.02; p_het_ < 0.001). Clinically administered tools also predicted increasing mortality across the full range of frailty severity in each of the six cohorts that allowed ordinal analysis (all p < 0.05). Moderate/severe vs no/mild frailty was also associated with a LOS >8 days (RR range = 2.14–3.04; n = 6) and discharge to a location other than home (RR range = 1.97–2.82; n = 4) but was inconsistently related to 30-day readmission (RR range = 0.83–1.94; n = 12). Associations remained clinically significant after adjustment for age, sex and comorbidity where reported.

**Interpretation:**

Frailty is common in older patients with acute, non-elective hospital admission and remains predictive of mortality, LOS and discharge home with more severe frailty associated with greater risk, justifying more widespread implementation of screening using clinically administered tools.

**Funding:**

None.

**Research in context**

**Evidence before this study**

Current guidelines recommend routine assessment for frailty in all hospitalised older adults to inform care. However, evidence is predominantly from specialty-specific or elective services whereas most hospital bed-days are accounted for by acute, unplanned admissions to generalist services in which the prevalence and prognostic value of frailty might differ. We therefore performed a systematic review and meta-analysis of observational studies using validated frailty measures in adult unplanned hospital-wide or general medicine admissions. We searched MEDLINE, EMBASE and CINAHL from inception to 31/01/2023 using terms relevant to [Frailty], [Geriatric Assessment], [Hospitalisation], [Outcomes] and [Observational Study] and included 45 cohorts (median/SD age = 80/5 years; n = 39,041,266 admissions, n = 22 measurement tools).

**Added value of this study**

We addressed an important evidence gap on frailty in unplanned hospital admissions. The prevalence of moderate/severe frailty ranged from 14.3% to 79.6%, with substantial heterogeneity (p_het_ < 0.001) unrelated to setting or frailty tool. Despite this variation, frailty was consistently associated with increased mortality, particularly in studies using clinically administered tools (n = 11; pooled RR = 2.53, 95% CI = 2.15–2.97, p_het_ = 0.08) vs studies using retrospective coding data, with evidence of a dose response effect. Frailty was also associated with increased length of stay (LOS) and discharge to a location other than home in all studies, but associations with readmission were conflicting. Associations remained significant after adjustment for age, sex, comorbidity and other confounders where reported.

**Implications of all the available evidence**

Frailty prevalence is two-to-four -fold higher in the older acute hospital population with unplanned admission than in community studies and about 50% higher than in the acute surgical setting. Frailty remains predictive of mortality, LOS and discharge destination in the unselected acute hospital setting with more severe frailty linearly associated with worse outcomes. Taken together, the available evidence justifies more widespread screening for both the presence and severity of frailty with clinically administered tools, such as the Clinical Frailty Scale, to inform care and target comprehensive geriatric assessment and interventions.

## Introduction

A large proportion of hospital inpatients are older persons: those aged ≥65 years account for 70% of hospital days in the UK, and 40–50% in the USA and Canada.^1^, ^2^, ^3^ Frailty, which is defined by a loss of physiologic reserve, is associated with reduced quality of life and poor health outcomes. Frailty is most common in older people although it may also be present in younger adults, for example with long-term health conditions such as multiple sclerosis or cancer.^4^^,^^5^ The importance of frailty in the acute care setting has been highlighted in several guidelines, which recommend routine screening for frailty in all older hospital patients using standard tools to inform clinical decisions, personalise care and target comprehensive geriatric assessment (CGA).^6^, ^7^, ^8^ In the Netherlands, screening has even been made mandatory under national legislation.^7^ The frailty construct, which is distinct from that of multi-morbidity, is commonly operationalised using the phenotype model comprising defined traits (for example, slow gait speed and weakness),^9^ the accumulation of deficits and related models (for example, the Hospital Frailty Risk Score-HFRS),^10^ or the pragmatic Clinical Frailty Scale (CFS), and can include cognitive function.^11^

Despite current guidance, uptake of hospital-wide frailty screening using standardised tools has generally been poor.^12^ Barriers to screening include uncertainty about the clinical utility of identifying frailty in acutely unwell patients, limited awareness of frailty tools and insufficient resources, with lack of evidence synthesis on frailty in the acute hospital setting likely a contributing factor.^13^^,^^14^ Most admissions to hospital are unplanned, predominantly to non-specialist general (internal) medicine services, where multi-morbidity and complex care needs are common. However, current guidance is based mostly on studies conducted in outpatient and specialty-specific or elective settings where the prevalence and prognostic value of frailty may differ. Reliable estimates of the burden of frailty in the acute hospital setting are needed to inform policy and plan services, including frailty screening programmes and CGA implementation.^12^ In addition, understanding the prognostic implications of frailty, and of different degrees of frailty, would help inform patient management particularly in settings where frailty is common. Current recommendations do not distinguish between more vs less severe frailty, even though care needs and prognosis are likely to vary considerably.^6^, ^7^, ^8^

Previous evidence synthesis on frailty in the acute hospital setting is limited.^14^ A scoping review (published 2018) included studies across a wide variety of acute settings in which the majority identified frail patients using non-validated methods.^15^ A systematic review (published 2019) on hospitalised older people included a large proportion of cohorts from geriatric medicine services or unspecified acute settings and was undertaken prior to the development and widespread uptake of measures using administrative data, primarily the HFRS.^16^ Other reviews and multi-centre studies have focused on specific settings and populations (e.g., surgery,^17^^,^^18^ including elective procedures,^19^, ^20^, ^21^, ^22^ acute coronary syndrome^23^). We therefore conducted a systematic review to determine i) the prevalence and measurement methods of frailty in adults with acute, unselected, non-elective admissions to hospital and general medicine services and ii) associations with mortality, length of stay (LOS), discharge destination and readmission, including after adjustment for confounding, and by degree of frailty.

## Methods

### Search strategy and selection criteria

This systematic review and meta-analysis was conducted as per the PRISMA guidelines^24^ and registered on PROSPERO (CRD42021235663).^25^ Ethical approval was not required for this study since it used only secondary data from existing published studies. MEDLINE, EMBASE and CINAHL were searched from inception to January 31, 2023 without restrictions using a search strategy developed in consultation with a healthcare librarian. Search terms related to frailty, geriatric assessment, hospitalization, outcomes and observational study design were included (Table S1–S3). We reviewed reference lists of included studies for other potentially eligible studies.

We included cross-sectional and cohort studies of adults ≥18 years with predominantly unplanned admissions (>70% unplanned as stated by the study authors or inferred from the study description) to hospital-wide or general (internal) medicine services, who received usual care. Unplanned hospital-wide and general medicine admissions were both included because the majority of unplanned admissions are to general medicine. Participants were required to be assessed for frailty using validated tools during their admission (Supplemental methods). We excluded studies conducted in outpatient, emergency department, short-stay, geriatric or rehabilitation and mixed settings or specialty-specific settings (except general medicine). One reviewer (ELB, STP, JMG) completed initial title-abstract and full-text screening and a second reviewer (JMG, STP) assessed studies independently where eligibility was unclear. Articles not in English were translated using Google Translate with help from colleagues fluent in the relevant language where needed. The final list of included studies was approved by ELB, SS and STP independently. Data were extracted by one researcher (ELB) using a standardised pro-forma including study and participant characteristics, recruitment method, frailty measurement tool and adjusted and unadjusted data stratified by moderate and severe frailty where reported. Data extraction was verified independently by a second researcher (STP). Risk of bias was assessed independently and in duplicate (ELB, STP) for prevalence and cohort outcomes using modified versions of the Joanna Briggs Institute Critical Appraisal Checklists for Prevalence and Cohort Studies (Tables S4 and S5).^26^^,^^27^ Discrepancies were resolved through discussion.

### Data analysis

Frailty categories (none, mild, moderate, severe frailty—see Box 1) were defined using accepted cut-offs for each tool where possible (Supplemental methods and Tables S6 and S7). For most analyses, we dichotomised frailty as moderate/severe vs no/mild, but also stratified data by the degree of frailty where relevant. For frailty prevalence, we calculated 95% confidence intervals (CI) for prevalence using Wilson's method for binomial proportions. Owing to high levels of heterogeneity across prevalence estimates, we did not undertake pooled analyses and instead reported the range across studies. We did a meta-regression to explore if heterogeneity was explained by differences in mean cohort age and performed pre-specified subgroup analyses i) for each frailty tool, as different tools might capture different aspects of frailty and ii) setting (general medicine vs all admissions). We assessed publication bias by meta-regression of prevalence against study sample size.

Box 1 **Examples of varying degrees of frailty.**

**No or mild frailty**: As defined by the Clinical Frailty Scale (CFS), a person with no frailty is independent with varying levels of activity (CFS of 1–3). A person with mild frailty may experience symptoms that limit activity or need help with high-order instrumental activities of daily living such as transportation and could have mild dementia (CFS 4–5). On the Hospital Frailty Risk Score (HFRS), a person is considered low risk if they have a total of 5 points or fewer from relevant ICD-10 coded conditions, for example chronic renal failure (1.40 points) and pneumonia (1.10 points).
**Moderate frailty:** As defined by the CFS, a person with moderate frailty needs help with outside activities, most instrumental and some basic activities of daily living (CFS of 6). They could also have moderate dementia. On the HFRS, a person is considered moderate risk if they have ICD-10 codes for conditions with a total of 5–10 points, for example chronic kidney disease (1.40 points), pneumonia (1.10 points) and a tendency to fall (3.60 points) or unspecified dementia (2.10 points).
**Severe frailty:** As defined by the CFS, a person with severe frailty is completely dependent for personal care (CFS of 7). They could also have severe dementia. A person with very severe frailty (CFS 8) is also completely dependent and probably would not recover from a minor illness. On the HFRS, a person is considered high risk if they have ICD-10 codes for conditions with a total of 15 points or more, for example chronic kidney disease (1.40 points), pneumonia (1.10 points), a tendency to fall (3.60 points), urinary incontinence (3.20 points), and dementia in Alzheimer's disease (7.10 points).

For the main outcomes analyses, we used data on moderate/severe vs no/mild frailty as this was most frequently reported. We assessed the association of moderate/severe frailty vs no/mild frailty with mortality up to one-year, hospital LOS, discharge to a new institution and readmission within 30-days. For dichotomous outcomes, we calculated relative risks (RR) from the data reported by the authors where at least one event was reported per group.

For continuous data (e.g., LOS), we calculated the ratio of means (ROM) for moderate/severe vs no/mild frailty. 95% CIs for dichotomous and continuous outcomes were determined from the normal approximation of the mean. When multiple estimates were reported for the same cohort (e.g., using different frailty measures), we took the estimate judged to have the best validity (i.e., internal, external, construct and conclusion). Adjusted estimates were extracted but were not used in the main outcomes analyses, because of differences in the reference groups used between studies, and we did not have access to individual patient data to calculate adjusted estimates. We did subgroup analyses based on whether frailty measures were designed for real-time administration by healthcare staff (“clinically administered tools”) or retrospective application usually to administrative data (“retrospective coding tools”). We assessed publication bias through funnel plots with Egger's test for asymmetry.

For the outcomes of discharge to a new institution and readmission, we endeavoured to restrict analyses to people alive at discharge because the competing risk of in-hospital death would otherwise result in underestimation of risk. Where not reported by the authors, we subtracted in-hospital deaths from the denominator. In cases where these data were not reported, we estimated in-hospital deaths based on data provided by the study authors (e.g., 30-day mortality including in-hospital deaths) or excluded the study from analysis. Sensitivity analyses were done using denominators as reported by the authors (e.g., including deaths during admission).

Data were pooled using a random-effects (DerSimonian and Laird) model with inverse-variance weights. Data were not pooled where important unexplained heterogeneity was present, which was evaluated based on differences between populations, measurement tools, setting, and study design; and by assessing differences in the direction of effects, and testing for statistical significance with the Chi^2^ test.^28^ Where it was not possible to perform meta-analysis, we narratively synthesised results.^29^

To assess for a trend in effect size (dose response effect) with increasingly severe frailty, we plotted outcomes stratified by degree of frailty in individual studies on forest plots and performed the Cochran–Armitage test for trend for each cohort. We also assessed the discriminative performance of categorical vs dichotomous frailty scores by calculating the apparent c-statistic to evaluate the prognostic value of differentiating between varying degrees of frailty.

Given differences in the likely sensitivity of different frailty measures and their operationalisation between studies (e.g., accuracy of administrative coding), variation in prevalence beyond that attributable to differences in case-mix and population was expected. Therefore, we explored the relationship between measured prevalence and the predictive value of frailty for mortality (log RR of death) in a meta-regression. The analysis was repeated with the addition of mean cohort age as a covariate in the model.

The certainty of evidence on outcomes was assessed using the Grading of Recommendations, Assessment, Development and Evaluation (GRADE) approach adapted for prognostic questions by ELB with input from SS and STP.^30^ Studies were downgraded from high-certainty evidence due to bias, imprecision, indirectness, unexplained inconsistency in the direction and magnitude of effects.^31^^,^^32^

Statistical analyses were performed in R version 3.6.3 (R Project for Statistical Computing).^33^ using the packages *meta, DescTools* and *pROC*.^34^, ^35^, ^36^

### Role of the funding source

The funder of the study had no role in study design, data collection, data analysis, data interpretation, or writing of the report. All authors (ELB, JMG, PM, SS, STP) had full access to all the data in the study and had final responsibility for the decision to submit for publication.

## Results

**Fig. 1:**
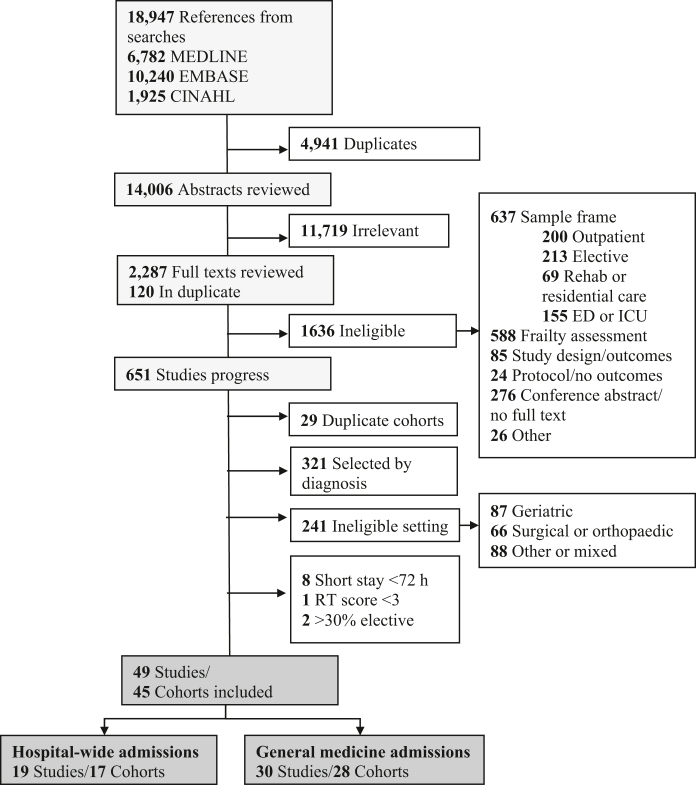
**PRISMA diagram**. PRISMA diagram showing the search results and process of study selection. ED = emergency department, ICU = intensive care unit, RT = reverse triage score.

We screened 14,006 abstracts and 2287 full texts, from which 45 cohorts (n = 39,041,266 admissions, 49 publications)^37^, ^38^, ^39^, ^40^, ^41^, ^42^, ^43^, ^44^, ^45^, ^46^, ^47^, ^48^, ^49^, ^50^, ^51^, ^52^, ^53^, ^54^, ^55^, ^56^, ^57^, ^58^, ^59^, ^60^, ^61^, ^62^, ^63^, ^64^, ^65^, ^66^, ^67^, ^68^, ^69^, ^70^, ^71^, ^72^, ^73^, ^74^, ^75^, ^76^, ^77^, ^78^, ^79^, ^80^, ^81^, ^82^, ^83^, ^84^, ^85^ were eligible for inclusion (Fig. 1). Most studies (30/45) aimed to investigate outcomes associated with frailty. All except one were published after 2010.^72^ The mean/SD age was 80/5 years (range = 64–87 years; 39 cohorts) and minimum age for inclusion was ≥65 years in 35/45 cohorts (Table 1, Table 2).

**Table 1:** Summary of included cohorts from studies of hospital-wide unplanned admissions (n = 17 cohorts).

Study	Location	N	Age	% Female	Exclusion criteria (summary)	Frailty tool	Type of tool
Asmus-Szepesi (2013)^37^	Netherlands	460	76	56%	Age <65 years, refused, terminally ill, unable to follow instructions, LOS <48 h	ISAR-HP	CA
Fujita (2022)^38^	Australia	6771	84	52%	Age <75 years, <30 eFI-AH deficits assessed	eFI-AH, HFRS	CR/RC, RC
GMRC (2019)^39^	England	1507	80	53%	Age <65 years, CCU, end-of-life, logistics, missing 4AT or delirium status	9-point CFS	CA
Gilbert—local cohort (2018)^40^	England	569	80	56%	Age <75 years	HFRS	RC
Gilbert—national cohort (2018)^40^	England	1,013,590	84	57%	Age <75, non-emergency admission	HFRS	RC
Gilbert (2022)^41^	France	1,042,234	85	60%	Age <75 years, non-emergency admission, missing socioeconomic data	HFRS	RC∗
Hollinghurst (2021)^42^	Wales	126,600	79	53%	Age <65 years, non-emergency admission, no GP registration	HFRS, eFI	RC∗, OC
Lim (2023)^43^	Singapore	366	74	56%	Age <65 years, ICU, transferred from other hospital, terminally ill, cognitive impairment/dementia, admitted for stroke or LOS <48 h	Frail-PPS, FAM, ISAR-HP	CA, CA, CA
Lujic (2022)^44^	Australia	257,535	83	57%	Age <75 years	HFRS	RC∗
McAlister (2018)^45^	Canada	452,785	83	60%	Age <75 years, psychiatric or non-urgent admission	HFRS	RC∗
Romero-Ortuno (2016a, 2016b)^46^^,^^47^	England	5899	84	56%	Age <75 years, elective admission	9-point CFS	CA∗
Soong (2015)^48^	England	50,540,141^a^	NR	50%	Age <65 years	Author	RC
Soong (2019)^49^^,^^50^	Multinational	1,366,187	NR	54%	Age <75 years, missing data, LOS <2 days	DF-GFS	RC
Street (2021)^51^	England	282,091^b^	NR	NR	Age <75 years, non-emergency admission	HFRS	RC∗
Timmons (2015)^52^	Ireland	598	80	51%	Age <70 years, refused, moribund on admission	SHARE-FI	CA
Wallis (2015)^53^	England	5764	85	56%	Age <75 years, non-emergency admission	9-point CFS	CA∗
Warnier (2017, 2019)^54^^,^^55^	Netherlands	2581^c^	79	51%	Age <70 years, not community-dwelling, not admitted to a regular ward, LOS <48 h, in hospital deaths (for outcomes data)	VMS, MFST-HP	CA, CA

Age = mean or median age in years. Author = Developed by author (Soong et al., 2015). CA = Clinically administered. CA∗ = Clinically administered, pre-morbid. CFS = Clinical Frailty Scale. CR = Clinical data from chart review. DF-GFS = Dr Foster Global Frailty Score. eFI = Electronic Frailty Index. eFI-AH = eFI Acute Hospital. FAM = Frailty Assessment Method. FRAIL = FRAIL questionnaire. Frail-PPS = Frail-Physical, Psychological and Social. HFRS = Hospital Frailty Risk Score. ISAR-HP = Identification of Seniors at Risk-Hospitalized Patients. MFST-HP = Maastricht frailty screening measure for hospitalised patients. N = Number of patients or admissions. NR = Not reported. OC = Other coded administrative data. RC = Retrospective coding data from the index admission acquired after discharge. RC∗ = Retrospective coding data from the index admission and previous admissions. SHARE-FI = Survey of Health, Ageing and Retirement in Europe-Frailty index. VMS (Veiligheids Management System) = Dutch National Safety Management Program.

a A count of frailty syndromes was only reported for ∼34,044,050 hospital admissions.

b n = 282,091 patients with 674,615 hospital admissions.

c n = 2691, but outcomes data were only reported for 2581 people who did not die in hospital so this number was used in analyses.

**Table 2:** Summary of included cohorts from studies of general medicine admissions (n = 28 cohorts).

Study	Location	N	Age	% Female	Exclusion criteria (summary)	Frailty tool	Type of tool
Anani (2020)^56^	Israel	980	72	43%	Age <55 or >85 years, no consent, advanced illness, bedridden, hospitalized <30 days prior, not admitted via ED	FRAIL	CA
Belga (2016)^57^, ^58^, ^59^	Canada	495	64	51%	Age <18 years, life expectancy <3 months, admitted from long-term care/other hospital, out-of-province, poor English, moderate-severe CI	9-point CFS, Fried, HFRS	CA∗, CA, RC∗
Bonjour (2021)^60^	Switzerland	22,323	80	52%	Age <65 years	HFRS	RC
Buurman (2012)^61^	Netherlands	639	78	54%	Age <65 years, no consent, too ill to participate, transferred from other ward, LOS or ward transfer <48 h, poor Dutch	ISAR-HP	CA
Dani (2017)^62^	England	710	83	59%	Age <70 years, LOS <48 h, poor English	FI	U
Eckart (2019)^63^	Switzerland	4957	82	51%	Age <75 years, non-urgent admission	HFRS	RC∗
Eeles (2012)^64^	Wales	273	82	NR	Age <75 years, no consent, readmission	FI	U
El-Sharkaway (2005)^65^	England	200	82	47%	Age <65 years, terminal illness, life expectancy <3 months	CFS	CA
Evans (2014)^66^	USA	751	84	64%	Age <75 years	FI-CGA	CR
Fitriana (2021)^67^	Indonesia	266	NR	NR	Age <60 years, died in-hospital, transferred to other hospital	FRAIL	CA
Forti (2014)^68^	Italy	470	81	53%	Age <65 years, died, terminal illness, coma, LOS or transfer <48 h, refused, incomplete data	SOF–I	CA
Gregoravic (2016)^69^	Australia	170	82	49%	Age <65 years, transferred to specialty unit	9-point CFS	CA
Hernandez-Luis (2018)^70^	Spain	298	77	53%	Age <61 years, life expectancy <6 months, delirium or impaired consciousness persisting on day two of admission, not admitted via ED	7-point CFS, Fried	CA, CA
Hoogerduijn (2012)^71^	Netherlands	492	78	56%	Age <65 years, too ill to participate	ISAR-HP	CA
Inouye (2003)^72^	USA	535	78	56%	Age <70 years, terminal condition, severe dementia, aphasia, coma, intubation, LOS <48 h, refused	BISEP	CA
Irina (2018)^73^	Israel	179	72	46%	Age <18 years, dementia/CI, ALT >40 IU/L, no follow-up, unable to complete FRAIL	FRAIL	CA
Juma (2016)^74^	Canada	75	81	64%	Age <65 years, palliative or life expectancy <7 days, <1 chronic health conditions and independent ambulation at baseline	9-point CFS	CA
Khandelwel (2012)^75^	India	250	66	38%	Age <60 years, mechanical ventilation/life support, comatose, neurologic deficits	Fried	CA
Laura (2022)^76^	Singapore	1507	76	51%	Age <65 years, live in shelter/nursing home, admitted via HDU, ICU or non-medical unit, no consent, died in hospital, transferred, discharged against medical advice	9-point CFS	CA
McCrow (2016)^77^	Australia	44	81	55%	Age <60 years, unstable CHF, severe CKD, nil by mouth on admission, expected LOS <24 h, poor English	7-point CFS	CA
Nardi (2019)^78^	Italy	541	80	51%	Age <40 years, <2 chronic diseases	FCS-1, MPI	CA, U
Noro (2011)^79^	Nordic countries	763	NR	65%	Age <75 years, CCU	MAPLe-AC	CA
Polidoro (2013)^80^	Italy	140	79	60%	NR	FI	U
Ramdass (2018)^81^	USA	503	80	54%	Age <65 years, refused, died in-hospital, advanced dementia, transferred from other facility, admitted under observation, not living in community	REFS	CA
Rizza (2021)^82^	Italy	80	82	46%	Age <75 years, unable to complete CGA, no consent, end-stage cancer or CKD, connective tissue or inflammatory bowel diseases, sepsis	MPI	U
Rose (2014)^83^	Australia	133	87	61%	Age <70 years, unable to complete REFS, refused	REFS	CA
Sharma (2022)^84^	Australia	263	84	52%	Age <65 years, no consent, terminally ill	REFS	CA
Subramanian (2020)^85^	Australia	1118	NR	NR	Age <80 years, LOS <24 h	HFRS	RC

Age = mean or median age in years. ALT = Alanine transaminase. BISEP = Burden of Illness Score for Elderly Persons. CA = Clinically administered. CA∗ = Clinically administered, pre-morbid. CCU = Coronary Care Unit. CFS = Clinical Frailty Scale. CHF = Congestive heart failure. CI = Cognitive impairment. CKD = Chronic kidney disease. CR = Clinical data from chart review. ED = Emergency Department. FCS-1 = FADOI-COMPLIMED Score 1. FI = Frailty Index. FI-CGA = Frailty index-CGA. FRAIL = Fatigue, Resistance, Ambulation, Illnesses, and Loss of weight questionnaire. Fried = Fried phenotype. HDU = High Dependency Unit. HFRS = Hospital Frailty Risk Score. ICU = Intensive Care Unit. N = Number of patients or admissions. ISAR-HP = Identification of Seniors at Risk-Hospitalized Patients. MAPLe-AC = Method for assigning Priority Levels-Acute Care. MPI = Multidimensional Prognostic Index. NR = Not reported. OC = Other coded administrative data. RC = Retrospective coding data from the index admission acquired after discharge. RC∗ = Retrospective coding data from the index admission and previous admissions. REFS = Reported Edmonton Frail Scale. SOF-1 = Study of Orthopaedic Fractures Index. U = Uncertain.

Forty cohorts in 17 high-income countries with universal healthcare, three in the USA, one each in India and Indonesia were included, largely from urban tertiary care or teaching hospitals. Seventeen cohorts included hospital-wide unplanned admissions (n = 39,002,111) and 28 included general medicine admissions only (n = 39,155). Prior to admission, 68–97% of participants lived at home (12 cohorts) and four studies limited eligibility to community-dwelling adults. The prevalence of any-cause cognitive impairment ranged from 1 to 68% (24 cohorts) and Charlson comorbidity scores ranged from 2 to 8 (14 cohorts). In general medicine cohorts, the most frequent (rank-sum) admission diagnoses were infection, circulatory or respiratory problems (Tables S8–S10).

Twenty-two frailty tools were identified (Tables S3–S11), including 12 developed for the hospital setting (eight clinically administered, three retrospective coding tools, one other). Deficit-accumulation methods were the most common (18/54 prevalence estimates from the 45 cohorts), followed by the CFS (10/54), and phenotype (9/54), multi-dimensional (7/54), brief (5/54) or other (5/54), but there were differences in the operationalisation of tools. For example, some studies calculated the HFRS using the index admission alone (n = 5), whereas others (n = 6) used the index and any other admissions in the previous two years. Three studies used the CFS to assess frailty status pre-admission (i.e., prior to the acute illness), but in the other seven, it was unclear how the CFS was operationalised.

The most common domains covered (Table 3) were mobility, balance or falls (n = 16), function (n = 14) and cognition (n = 14 overall, n = 7 delirium). Twelve assessed psychosocial factors. Data sources included administrative data, questionnaires, physical/functional assessments and chart review (Table S12).^66^^,^^72^ Cognition was assessed using self/proxy report in two measures,^55^^,^^86^ chart review or diagnostic coding in seven^40^^,^^42^^,^^48^^,^^49^^,^^66^^,^^72^^,^^79^ and objective testing in five.^10^^,^^38^^,^^43^^,^^87^^,^^88^ The number of items included ranged from 4 to 6 for brief, 3–9 for phenotype, 8–18 for multidimensional and 31–109 for deficit accumulation tools. All clinically administered tools required additional assessments by staff or questionnaires, but only three studies reported the time required and none evaluated feasibility further.

**Table 3:** Summary of domains included in frailty measures.

CSHA CFS = CSHA Clinical Frailty Scale; ISAR-HP = Identification of Seniors at Risk-Hospitalized Patients; VMS (Veiligheids Management Systeem) = Dutch National Safety Management Program; FAM = Frailty Assessment Method; FRAIL = FRAIL questionnaire; SHARE-FI = SHARE-Frailty index; SOF-1 = Study of Orthopaedic Fractures Index; HFRS = Hospital Frailty Risk Score; eFI = Electronic Frailty Index; eFI-AH = eFI Acute Hospital; FI = Searle et al., 2008 Frailty Index; FI-CGA = Frailty index-CGA; REFS = Reported Edmonton Frail Scale; MFST-HP = Maastricht frailty screening measure for hospitalised patients; MPI = Multidimensional Prognostic Index; MAPLe-AC = Method for assigning Priority Levels-Acute Care; BISEP = Burden of Illness Score for Elderly Persons; FCS-1 = FADOI-COMPLIMED Score 1; Frail-PPS = Frail-Physical, Psychological and Social; DF-GFS = Dr Foster Global Frailty Score; Soong et al., 2015 = Developed by author (Soong et al., 2015).

^a^Originally developed for use in hospital settings.

^b^Complete information on these frailty tools was not available.

^c^Multiple variations of the FI were included in this review.

Risk of bias was low-moderate for 26/45 studies of prevalence (Fig. S1), with common sources of bias related to sample frame and coverage, for example due to restrictive eligibility criteria (Table S13). Risk of bias was low/moderate for 27/37 studies of outcomes (Fig. S2) and the most common source of bias was confounding by comorbidity and illness severity (Table S13). There was no evidence of publication bias in frailty prevalence (Fig. S3) or mortality outcomes (Fig. S4). Certainty of the evidence (GRADE) is reported in Table S14.

**Fig. 2:**
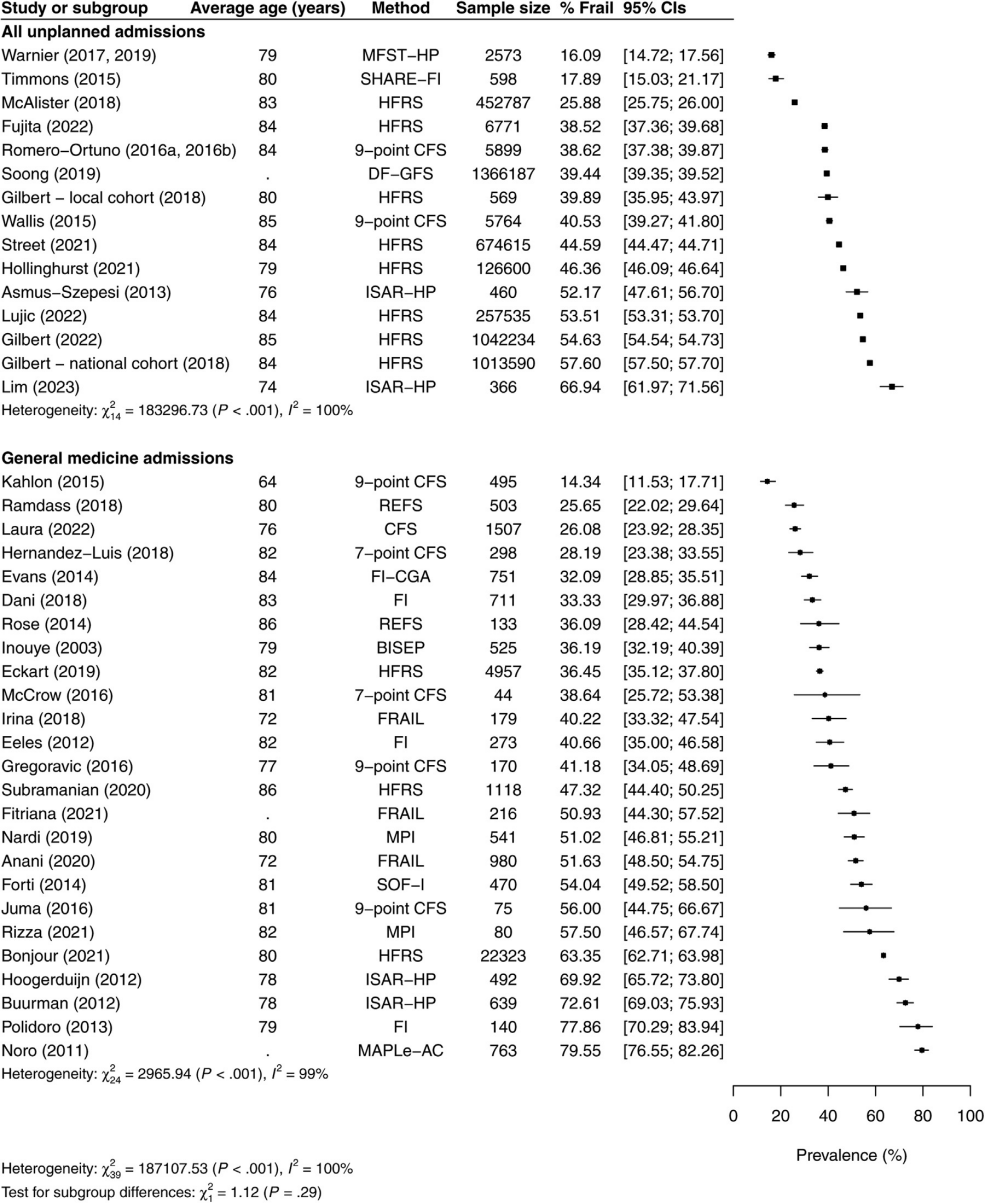
**Prevalence of moderate/severe frailty stratified by study setting**. Forest plots of % prevalence with 95% CI of moderate/severe frailty stratified by hospital-wide unplanned vs general medicine admissions.

The prevalence of moderate/severe frailty ranged from 14.3 to 79.6 in all cohorts (N = 40 cohorts; n = 4,994,931 admissions), and in the 26 cohorts with low-moderate risk of bias, with rates <25% in only 3/45 cohorts. Moderate frailty was more common (range 10.0–50.7%) than severe frailty (range 2.2–39.6%; Fig. S5) in 18/22 cohorts reporting both (p < 0.01). Data were not pooled due to heterogeneity, which remained after stratification by study setting, frailty tool, and risk of bias (p_het_ < 0.001) and was not explained by average cohort age in meta-regression (y-intercept = −1.5402, beta = 0.0174; *R*^*2*^ = 2.02%; p = 0.36). The prevalence of moderate/severe frailty appeared similar between hospital-wide (range 16.1–66.9%; N = 15 cohorts; n = 4,956,548 admissions) and general medicine cohorts (range 14.3–79.6%; N = 25 cohorts; n = 38,383 admissions) (Fig. 2) and for most tools except for brief measures for which prevalence ranged from 52.2 to 72.6% (N = 5 cohorts; n = 4530) (Fig. S6).

**Fig. 3:**
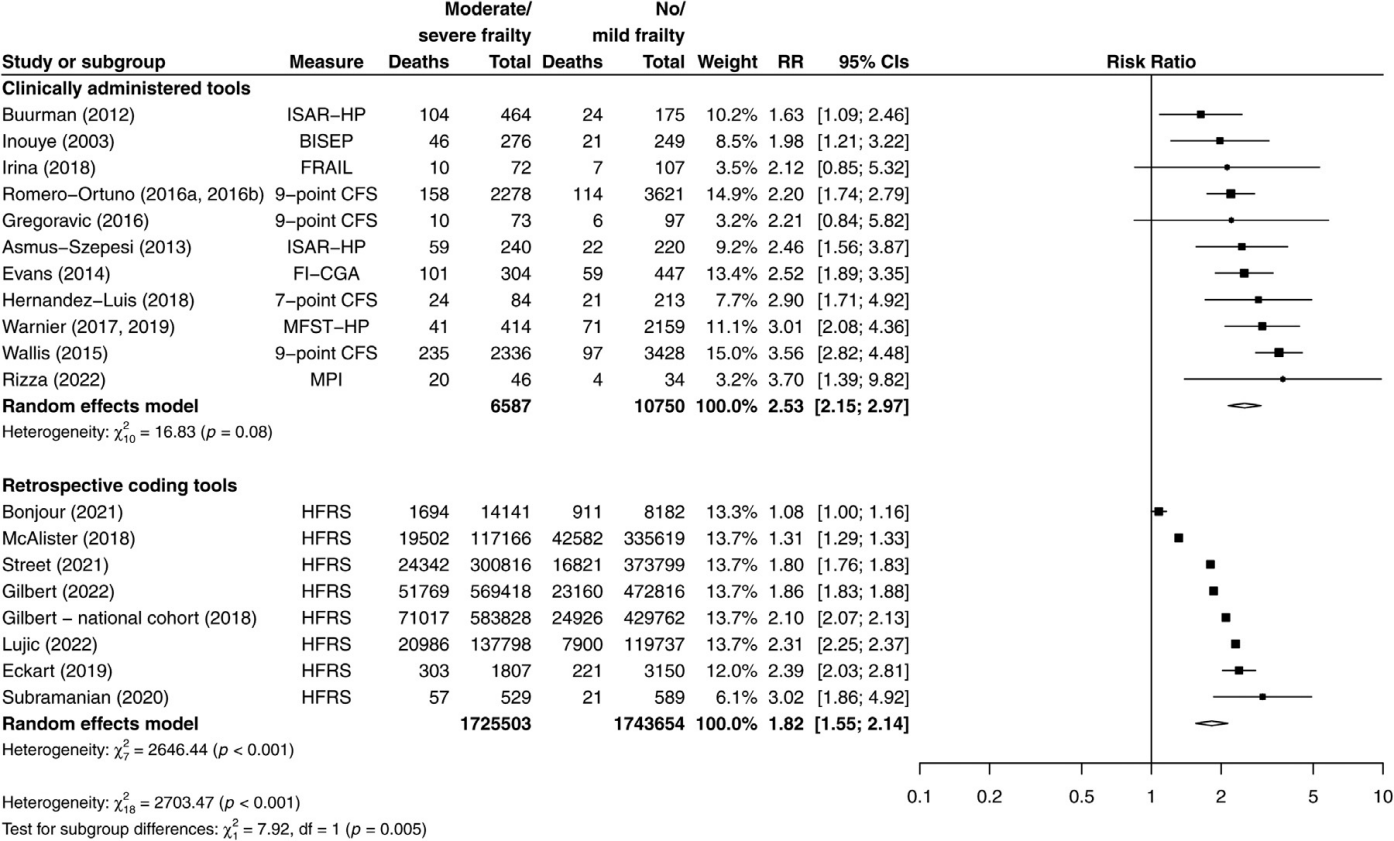
**Unadjusted relative risks of mortality for moderate/severe versus no/mild frailty by type of measure**. Unadjusted relative risks of death with 95% CI for moderate/severe vs no/mild frailty in studies using clinically administered tools versus retrospectively applied tools using administrative diagnostic coding. To avoid artificially reducing the standard error, when multiple estimates were reported for the same cohort (i.e., using different frailty measures), the estimate judged to have the best validity (e.g., validated in a similar setting previously or based on included constructs) was included in the pooled estimate. Estimates not included in the pooled estimate included Warnier et al. (2017, 2019), which reported a RR for 30-day mortality of 8.97 (95% CI 4.71–17.10). Studies with no events in either group are not shown (i.e., Juma 2016 and Khandelwel 2012), but are included in Fig. S8.

Consistent associations with mortality, LOS and discharge to a location other than home were found across all levels of frailty. Moderate/severe frailty was associated with an increased risk of death up to one year after hospital admission in 20/21 cohorts for which data were available (n = 3,486,819) but study estimates were not pooled due to heterogeneity in the absolute size of the effect (p_het_ < 0.001) (Fig. S7). However, when restricted to studies using clinically administered measures, heterogeneity was reduced (p_het_ = 0.08), with a pooled RR of 2.53 (95% CI 2.15–2.97; n = 17,337; N = 11; Fig. 3 and Fig. S8).

**Fig. 4:**
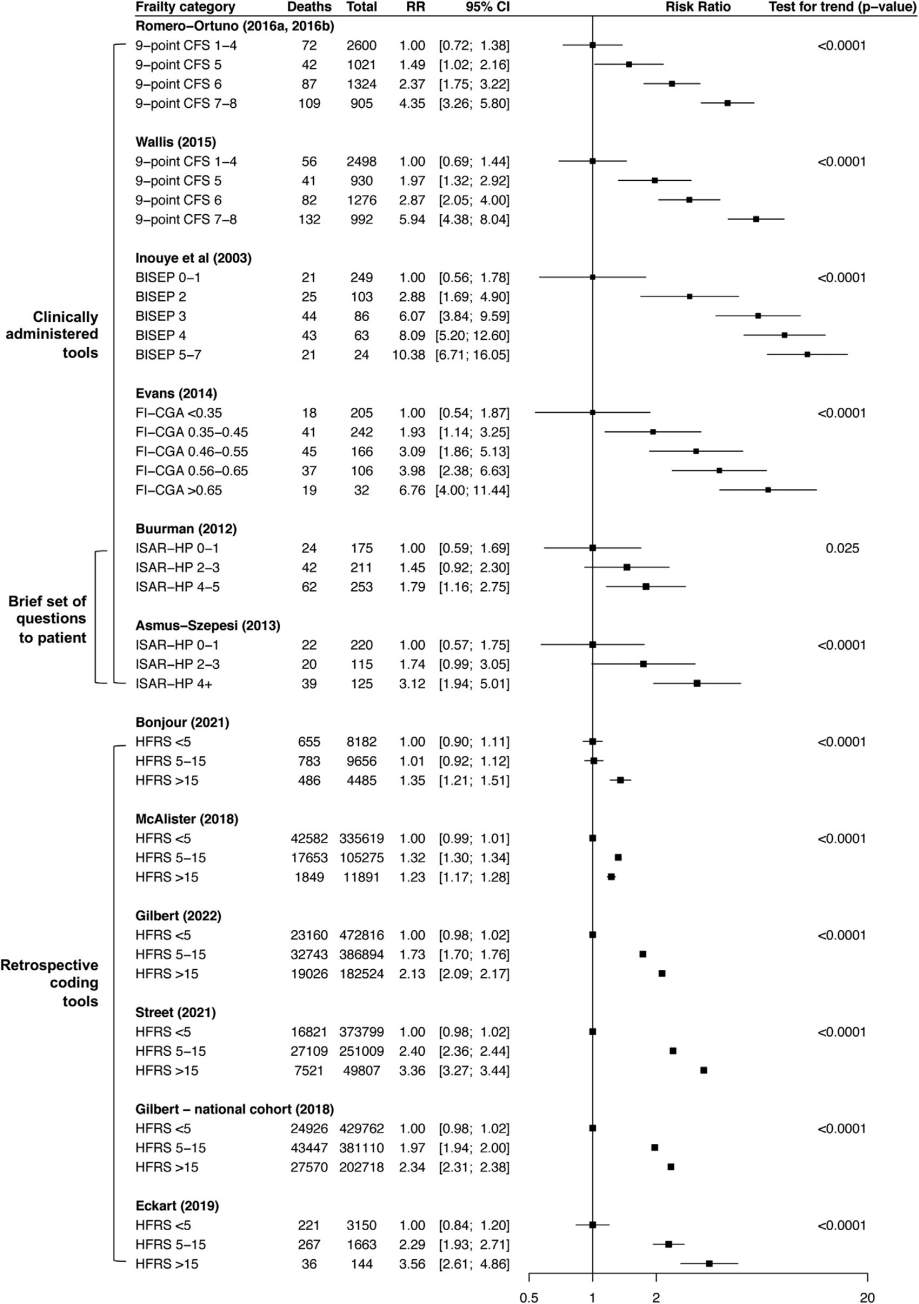
**Unadjusted relative risks for mortality up to one year after discharge across ordinal categories of degree of frailty and two-sided Cochran–Armitage test for trend**. Unadjusted relative risks with 95% CI for death up to one year after discharge by degree of frailty. Studies with >5 events per cell were included. Romero-Ortuno et al. (2016a, 2016b) only reported data for people with CFS 1–8. Dani et al. (2018) (not shown) reported data on mortality up to 3 years and found 48% mortality among people in the first FI tertile, 51% in the second and 60% in the third (n = ∼237 per group). P-values for the two-sided Cochran Armitage test for trend are shown.

Increasing severity of frailty was associated with a stepwise increase in the risk of death up to one year after hospital admission for all six studies using clinically administered (CFS, brief and multidimensional) tools and four of six using retrospective frailty coding tools for which data were available (p for test of binomial proportions = 0.039) suggesting a dose response effect (Fig. 4, Figs. S9 and S10). Discrimination (c-statistic) also improved when ordinal instead of dichotomous frailty groups were used for both clinically administered and retrospective coding tools (Table S17).

Meta-regression of frailty prevalence vs the log RR of death showed that frailty prevalence accounted for half of the variation in mortality risk (y-intercept = 1.0022; beta = −0.0069; *R*^*2*^ = 60.38%; p = 0.04) (Fig. S10). The addition of average cohort age to the model did not account for additional heterogeneity (y-intercept = −1.099, beta_frailty_ = −0.0070; beta_age_ = 0.0255; *R*^*2*^ = 53.42%; p_frailty_ = 0.05; p_age_ = 0.16).

**Fig. 5:**
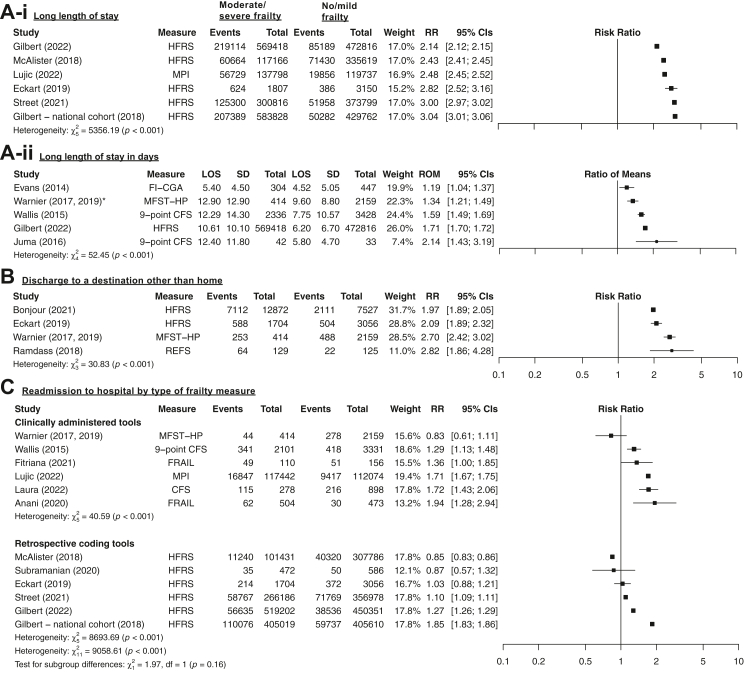
**Unadjusted relative risks of moderate/severe vs no/mild frailty for (A) LOS >8–10 days and ratio of means for LOS in days, (B) discharge to location other than home in survivors and (C) 30-day readmissions stratified by type of frailty measure in survivors**. Unadjusted relative risks with 95% CI of moderate/severe vs no/mild frailty for (A-i) LOS >8–10 days with (A-ii) ratio of means for LOS in days, (B) discharge to location other than home (i.e., nursing home or post-acute care facility) in survivors and (C) 30-day readmissions stratified by type of frailty. For LOS, Forti (2014) was not shown because it excluded in-hospital deaths (relative risk of 1.35, 95% CI 1.11–1.64). In-hospital deaths were estimated for Gilbert (2018) by multiplying the number of 30-day deaths after the date of admission (including in-hospital deaths) for each frailty category by the % of overall deaths that occurred in hospital and McAlister (2018) by using the n and % of survivors with readmissions and may be subject to rounding errors. In-hospital deaths were assumed to be absent from Anani (2020).

**Fig. 6:**
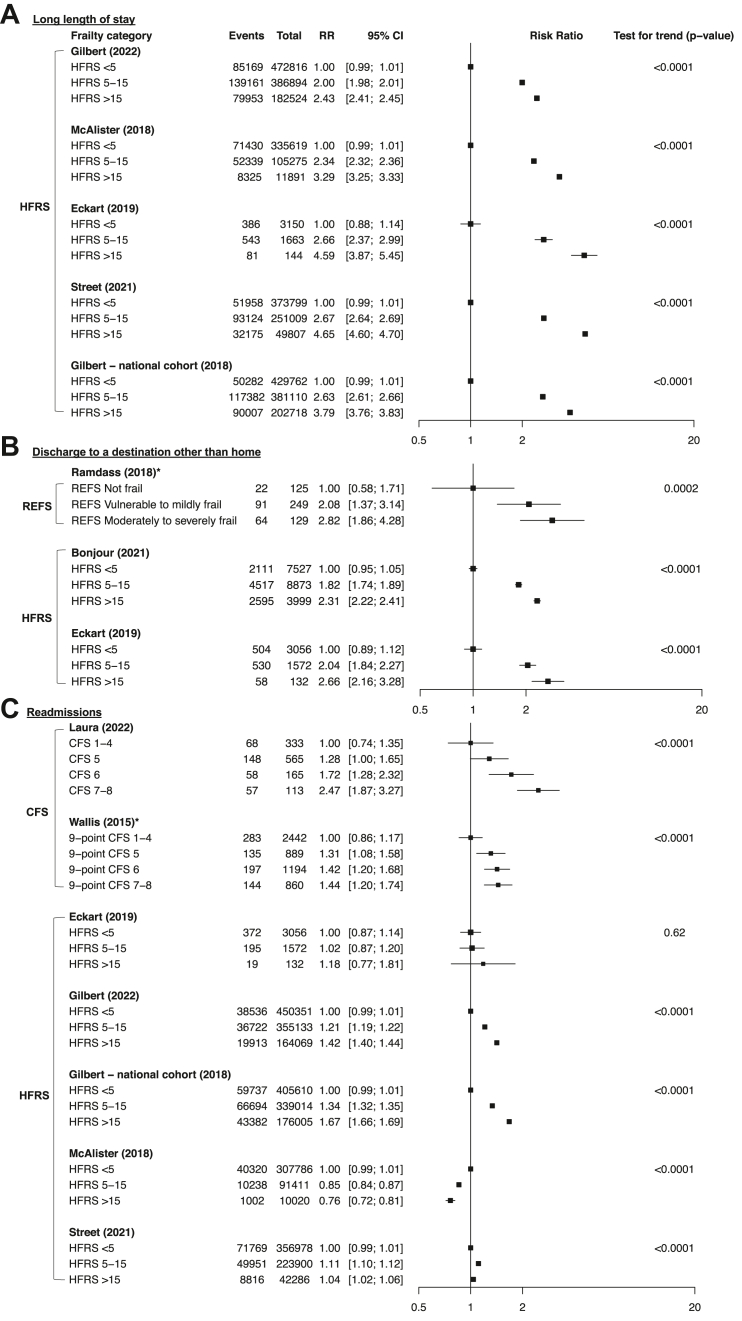
**Unadjusted relative risks across ordinal categories of degree of frailty and two-sided Cochran–Armitage test for trend for (A) long LOS, (B) discharge destination other than home in survivors and (C) readmissions in survivors**. Unadjusted relative risks with 95% CI by degree of frailty for (A) long LOS, (B) discharge destination other than home in survivors and (C) readmissions in survivors. Studies with >5 events per cell were included. P-values for the two-sided Cochran–Armitage for trend (increasing or decreasing) are shown.

All ten studies examining the risk of a longer LOS by frailty status showed an increase in LOS for moderate/severe frailty (n = 3,454,879 admissions). Relative risk for a LOS of >8–10 days ranged from 2.14 to 3.04 for six cohorts (n = 3,445,716) inclusive of inpatient deaths (Fig. 5A), with increasing severity of frailty associated with greater risks in all studies (Fig. 6A). The ratio of means for LOS in days ranged from 1.19 to 2.14 for moderate/severe frailty (n = 1,051,397 admissions; N = 5 cohorts; Fig. 5A). Sensitivity analyses where inpatient deaths were censored showed similar results (Fig. S11A).

All four studies examining discharge to a destination other than home in participants alive at discharge (n = 27,986) also reported increased risks for participants with moderate/severe frailty. The relative risk for discharge to a destination other than home ranged from 1.97 to 2.82 (Fig. 5B), with increasing severity of frailty associated with greater risks in 3/3 studies (Fig. 6B). Similar results were obtained using the denominators reported by the authors (e.g., including patients who died during admission, Fig. S11B).

The risk of 30-day readmissions in moderate/severe frailty showed conflicting findings with variation in the direction of effect in participants alive at discharge (n = 3,055,748; N = 12 cohorts). RRs ranged from 0.83 to 1.94 with 4/12 cohorts showing a reduced or unchanged risk of readmission, not explained by stratification by study setting (Fig. S12) or clinically administered vs retrospective coding tools (Fig. 5C). Similar results were obtained using the denominators reported by the authors (e.g., including patients who died during admission, Fig. S11C).

Regarding adjusted analyses for outcomes, five individual studies adjusted for a minimum of age, sex and comorbidity and also reported unadjusted estimates (Table S15). Odds ratios (adjusted relative risks were not available) were a mean of 15% lower than crude estimates for moderate frailty and 20% lower for severe frailty (Table S16). Eleven studies variably adjusted for multiple covariates including multi-morbidity (and did not report unadjusted estimates), but nevertheless found clinically important associations of frailty with death. Risk of death remained significant after adjustment/stratification for illness severity on admission (i.e., NEWS, MEWS) in two studies.^47^^,^^63^ Additional adjusted estimates are reported in the supplementary material (Table S15), but the majority could not be pooled due to differences in reporting and statistical heterogeneity (Figs. S13 and S14).

## Discussion

Frailty was common in the older acute unselected hospital population with a median prevalence of around 40%, although estimates varied. Despite the wide variation in prevalence, moderate to severe frailty remained an independent predictor of mortality up to one-year, longer LOS and discharge to a care home, although the evidence on readmission risk was conflicting. More severe frailty associated with worse outcomes, suggesting a dose response effect. Associations were strongest and most consistent across studies using clinically administered tools, but variation in measured frailty prevalence also accounted for a large proportion of statistical heterogeneity in mortality risk ratios.

Variation in frailty prevalence could not be attributed to average age, setting or frailty measure. Differences in eligibility criteria, such as the need to be able to complete questionnaires or give consent will have impacted case-mix and probably frailty prevalence. The proportion of participants who were care home residents or had cognitive impairment varied widely, although definitions differed. Not all frailty measures included cognition (or delirium, a marker of cognitive frailty), potentially resulting in underestimation of prevalence.^89^ Further, regional differences in demographics, health and social services may have had an impact including number of hospital beds, and access to ambulatory emergency care which may reduce inpatient admissions preferentially amongst younger, fitter people.^90^^,^^91^

Differences in the administration/operationalisation of frailty measures might also contribute to heterogeneity in measured prevalence. For example, retrospective, administrative data-based tools depend on the accuracy of diagnostic coding, which is variable and known to be poor for frailty syndromes.^92^ However, associations between frailty and poor outcomes held irrespective of the frailty tool administered, indicating that all measures identified a group at risk with different tools probably capturing different but overlapping groups.^57^ Studies with higher frailty prevalence generally reported weaker associations with mortality possibly because these studies operationalised frailty in a more inclusive way, identifying some individuals as frail who would have been classed as fit in other studies.

Mortality risk ratios for clinically administered frailty tools appeared quantitatively similar. However, mortality risk ratios for retrospective coding tools varied widely and accuracy of administrative coded data was again, likely a contributing factor. More severe frailty was consistently associated with worse outcomes, supporting the construct of ‘frailty’ as a spectrum rather than a dichotomous state. Future studies may therefore reasonably assume a linear relationship between frailty and mortality as recently described specifically for frailty indices.^93^ Associations between frailty and mortality remained significant after adjustment for multimorbidity and also for severe illness where reported, both factors being prevalent in acute hospital settings.^94^ We also observed a consistent direction of relationship between frailty and LOS and discharge destination, again with a stepwise increase in effect size although the size of the effect varied, possibly because these outcomes are dependent on healthcare system factors.^95^^,^^96^

Comparing our findings with previous studies, frailty prevalence was two-to-four fold higher in the acute hospital setting than in population-based studies (consistent with frailty being a risk factor for hospitalization)^91^^,^^97^ and about 50% higher than in the acute surgical setting.^98^^,^^99^ The median prevalence in our study was lower (∼40% vs 51%) than in general medicine studies using non-validated as well as validated frailty tools^15^ but similar to findings in geriatric medicine/unspecified acute hospital settings (25–97%).^15^ Our findings on mortality are broadly consistent with the greater risk reported in specialist hospital services (2–4 fold increase) and in population-based samples (2-fold increase).^15^^,^^17^, ^18^, ^19^^,^^21^^,^^23^^,^^100^^,^^101^ Specialty-specific studies also found associations with LOS and discharge destination.^21^^,^^23^^,^^100^ Stepwise increases in risk of death have been reported for ‘pre-frailty’ (two-fold increase compared no frailty) and frailty (3.5-fold increase).^16^

Strengths of our review include a comprehensive literature search with detailed synthesis of evidence from a large number of unselected cohorts enhancing generalizability and filling an important evidence gap. We compared clinically administered and retrospective coding frailty tools, examined degrees of frailty and accounted for a proportion of statistical variation. Our review has limitations. First, associations between frailty and readmission risk were inconsistent, and not explained by setting, tool used or the competing risk of death where reported.^45^ This finding was not unexpected, however, because a broad range of healthcare system factors are known to impact readmission risk.^102^, ^103^, ^104^ Second, we could not account for case-mix factors in prevalence estimates using meta-regression or subgroup analysis because of limitations in reporting. Data on cognition were limited, so its impact on outcomes could not be evaluated despite the implications for patient care.^62^^,^^64^ Third, categorisations of moderate and severe frailty were based on accepted cut-offs and author's judgement, but were approximations. Fourth, the applicability of our results to people <65 years is uncertain since they were excluded from the majority of studies.

Our findings provide evidence to support robust implementation of frailty screening in acute hospitals to inform decision making and the targeting of interventions/CGA (see Box 2).^12^ Frailty should inform clinical care through an understanding of likely outcomes but should not be used in isolation to direct clinical decisions. Importantly, future guidelines, policy documents and health economic analyses should also differentiate between varying degrees of frailty. More specifically, our findings support current guidance recommending the CFS as a first line screening tool since it is pragmatic but nevertheless identifies a group at-risk as reliably as more lengthy/complex tools.^6^ However, we found variations in CFS operationalisation in the acute setting, supporting the need for training.^13^ Also, because the CFS is a global frailty score only, further assessment is required to identify frailty domains and fully individualise care. Other clinically administered tools appeared impractical for first line routine use and despite their length, most did not include valid measures of cognition or social risk factors, which are both important drivers of admission.^91^^,^^92^^,^^105^, ^106^, ^107^, ^108^

Box 2 **How data on the prevalence, measurement tools used, and outcomes of frailty in the acute setting can inform policy, planning and care.**

**Policy**

• Staffing levels and skill mix calculations
• Frailty training requirements
• Resourcing and service design

**Service planning**

• Development of frailty care pathways
• Need for Comprehensive Geriatric Assessment and multidisciplinary team care
• Case-mix evaluation

**Clinical care**

• Frailty measures as communication tools for handover and transfers
• Individualisation of acute treatment according to frailty status
• Risk-stratification for further needs assessment or specialised frailty care
• Enhanced discharge planning and strengthening of post-discharge care
• Improved patient and caregiver experience including counselling about prognosis
  • Advanced care planning and power of attorney
  • Readmission avoidance strategies

Several retrospective tools based on administrative data (primarily the HFRS) were identified, which are useful for policy or research purposes. Importantly, retrospective coding frailty tools may incorporate frailty syndromes occurring as complications of admission (e.g., falls, pressure sores) in contrast to prospective clincal tools. In case-mix adjustment for mortality and other acute hospital outcomes, retrospectively acquired frailty measures may therefore conceal preventable safety and quality issues.^109^, ^110^, ^111^

In conclusion, frailty is prevalent in older people with acute, non-elective hospital admissions and is an important independent prognostic factor, with a dose response effect. Our findings support robust implementation of hospital-wide frailty screening in line with current guidance.^6^^,^^8^ Challenges remain around the large scale implementation of CGA which is time consuming and requires multi-disciplinary input. Future studies may explore the use of real-time rich clinical information in hospital electronic health records (i.e., beyond just retrospective diagnostic codes) to identify and monitor frailty and patients’ domain-specific needs, prior to discussions with patients and their families, to reduce the burden to patients and staff. Such a “streamlined CGA” could also be used by (virtual) frailty teams to provide individualised recommendations, goal-setting and decision-making in partnership with patients and carers. These methods should also differentiate between varying degrees of frailty and could enhance the quality of routinely acquired frailty data available for policy and research.

## Contributors

ELB was responsible for the protocol, data acquisition (i.e., running searches, screening articles, extracting data, risk of bias assessment), analysis, interpretation and drafting the manuscript. JMG contributed to the protocol, screened articles and reviewed the final list of studies for inclusion. PMR contributed to the interpretation of data and drafting of the manuscript. SS provided supervision and contributed to study design, development of the protocol, approval of the final list of studies for inclusion, the interpretation of data, and drafting of the manuscript. STP provided supervision and was responsible for study conception, design, development of the protocol, approval of the final list of studies for inclusion, data acquisition (i.e., risk of bias assessment) and interpretation, and drafting of the manuscript. The underlying data was accessed and verified by ELB and STP. All authors (ELB, JMG, PM, SS, STP) had full access to all the data in the study and had final responsibility for the decision to submit for publication.

## Data sharing statement

Data extracted from studies will be made available upon reasonable request by email to sarah.pendlebury@ndcn.ox.ac.uk.

## Declaration of interests

We declare no competing interests.
