# Supplementary material for: Prevalence and outcomes of frailty in unplanned hospital admissions: a systematic review and meta-analysis of hospital-wide and general (internal) medicine cohorts
Source: eClinicalMedicine. 2023 Apr 21;59:101947. doi: 10.1016/j.eclinm.2023.101947 (PMC10149337; doi:10.1016/j.eclinm.2023.101947)
Supplement: Supplementary material [file mmc1.pdf]

**Supplementary Material**

**Table of Contents**

**Supplemental methods ______ 3**

Search strategy ______ 3

Table S1. Search strategy for Medline 3

Table S2. Search strategy for EMBASE 5

Table S3. Search strategy for CINAHL 7

Supplementary Methods ______ 8

Eligibility criteria for frailty tools 8

Potential bias related to the definition of the denominator 8

Modified Joanna Briggs Institute tools for risk of bias assessment ______ 9

Table S4. Modified Joanna Briggs Institute Checklist for Prevalence Studies 9

Table S5. Modified Joanna Briggs Institute Checklist for Cohort Studies 10

Cut-points selected for methods of frailty ascertainment with more than one frailty category ______ 12

Table S6. Ordinal frailty cut-points for data synthesis and meta-analysis. 12

Table S7. Dichotomous frailty cut-points for data synthesis and meta-analysis. 12

**Supplemental results - tables ______ 13**

Table S8. Summary of characteristics of included studies and prevalence estimates ______ 13

Table S9. Summary of included cohorts from studies of all hospital admissions ______ 14

Table S10. Summary of included cohorts from studies of general medicine admissions ______ 17

Table S11. Summary of frailty assessment tools used in included studies ______ 23

Table S12. Summary of data sources used in frailty instruments ______ 28

Table S13. Study-level risk of bias assessment for all admissions and general medicine admissions. _28

Table S14. GRADE summary of findings table for main study outcomes ______ 38

Table S15. Adjusted odds ratios and other measures for main study outcomes ______ 39

Table S16. Percent difference between crude and age/sex adjusted and most adjusted estimates ______ 45

Table S17. C-statistic (area-under-the-receiver-operator-curve) and 95% CIs for study outcomes ______ 46

**Supplemental results - figures ______ 47**

Figure S1. Summary of risk of bias across studies for prevalence ______ 47

Figure S2. Summary of risk of bias across studies for cohort outcomes ______ 48

Figure S3. Prevalence of moderate-to-severe frailty plotted against sample size and meta-regression equation stratified by setting ______ 49

Figure S4. Figure S4. Funnel plots with Egger's test for the unadjusted relative risks of mortality for moderate/severe versus no/mild frailty stratified by type of measure ______ 50

Figure S5. Prevalence of moderate and severe frailty ______ 51

Figure S6. Prevalence of moderate/severe frailty stratified by method of ascertainment ______ 52

Figure S7. Unadjusted relative risks of mortality stratified by time-point for moderate/severe versus no/mild frailty ______ 53

Figure S8. Unadjusted relative risks of moderate/severe versus no/mild frailty stratified by frailty tool type, including studies with no events in either group 54

Figure S9. Unadjusted relative risks of mortality (clinically administered tools only) for moderate and severe versus no/mild frailty 55

Figure S10. Bubble plot for meta-regression of moderate/severe frailty prevalence versus the log of the relative risk for all-cause mortality 56

Figure S11. Unadjusted relative risks for (A) length of stay >8-10 days (excluding those who died in hospital), (B) discharge to a destination other than home (nursing home or post-acute care facility) using overall denominator reported by the study authors, and (C) readmission to hospital using overall denominator reported by the study authors 57

Figure S12. Unadjusted relative risks of readmission for moderate/severe versus no/mild frailty in people who survived to discharge, stratified by setting 58

Figure S13. Adjusted odds (A) and hazard (B) ratios for mortality by degree of frailty 59

Figure S14. Adjusted odds ratios for (A) long length of stay and (B) 30-day readmission 60

**PRISMA Checklist 61**

**Supplemental references 63**

# Supplemental methods
## Search strategy

**Table S1. Search strategy for Medline** (Ovid MEDLINE® Epub Ahead of Print, In-Process & Other Non-Indexed Citations, Ovid MEDLINE® Daily and Ovid MEDLINE®) 1946 to present

| # | Searches |
|---|---|
| 1 | Frailty/ |
| 2 | Frail elderly/ |
| 3 | Geriatric Assessment/ |
| 4 | (frail* adj3 (assessment or index or scale or phenotype or indicator or risk or score or framework or tool or screen* or syndrome)).mp. |
| 5 | debilit*.tw,kf. |
| 6 | frail*.tw,kf. |
| 7 | "physical frailty phenotype".tw,kf. |
| 8 | "deficit accumulation".tw,kf. |
| 9 | "clinical frailty scale".tw,kf. |
| 10 | "Fried frailty".tw,kf. |
| 11 | "brief frailty index".tw,kf. |
| 12 | ("frail scale" or frail-scale).tw,kf. |
| 13 | "modified frailty index".tw,kf. |
| 14 | interRAI*.tw,kf. |
| 15 | ((vulnerable adj (elders* adj survey*)) or VES-13).tw,kf. |
| 16 | "Edmonton Frail Scale".tw,kf. |
| 17 | "frailty staging system".tw,kf. |
| 18 | "Conselice Study of Brain Aging Score".tw,kf. |
| 19 | ("geriatric screening tool" or G-8).tw,kf. |
| 20 | "multidimensional prognostic index".tw,kf. |
| 21 | "kihon checklist".tw,kf. |
| 22 | (("Cardiovascular Health Study" or CHS) adj2 criteria).tw,kf. |
| 23 | ("hospital frailty risk score" or HFRS).tw,kf. |
| 24 | ("electronic frailty index" or eFI).tw,kf. |
| 25 | "geriatric assessment".tw,kf. |
| 26 | or/1-25 |
| 27 | Hospitalization/ |
| 28 | Patient Admission/ |
| 29 | Inpatients/ |
| 30 | (in#patient or in#hospital or hospital*).tw,kf. |
| 31 | (admitted or admission*).tw,kf. |
| 32 | or/27-31 |
| 33 | Prevalence/ or prevalence.tw,kf. |
| 34 | outcome*.tw,kf. |
| 35 | Hospital mortality/ |
| 36 | mortality.tw,kf. |
| 37 | death*.tw,kf. |
| 38 | Survival analysis/ or Proportional hazards/ |
| 39 | surviv$.tw,kf. |
| 40 | Patient Readmission/ |
| 41 | (readmission* or re-admission* or readmitted or re-admitted).tw,kf. |
| 42 | (re-hospital* or rehospital*).tw,kf. |
| 43 | return.tw,kf. |
| 44 | recidivism.tw,kf. |
| 45 | (re-attend* or reattend*).tw,kf. |
| 46 | (re-visit* or revisit*).tw,kf. |
| 47 | exp Length of Stay/ |
| 48 | ((length or duration) adj3 (stay or hospital or admission*)).tw,kf. |
| 49 | or/33-47 |
| 50 | Epidemiologic studies/ |

| | |
|---|---|
| 51 | exp Cohort studies/ |
| 52 | exp Case control studies/ |
| 53 | cohort.mp. |
| 54 | "case control".mp. |
| 55 | (observational adj (study or studies)).mp. |
| 56 | ((Follow-up or "follow up") adj (study or studies)).mp. |
| 57 | (prospective or retrospective).mp. |
| 58 | or/50-57 |
| 59 | 26 and 32 and 49 and 58 |

**Table S2. Search strategy for EMBASE** 1974 to present

| # | Searches |
|---|---|
| 1 | Frailty.tw,kw. |
| 2 | Frail elderly/ |
| 3 | Geriatric Assessment/ |
| 4 | (frail* adj3 (assessment or index or scale or phenotype or indicator or risk or score or framework or tool or screen* or syndrome)).mp. |
| 5 | debilit*.tw,kw. |
| 6 | frail*.tw,kw. |
| 7 | "physical frailty phenotype".tw,kw. |
| 8 | "deficit accumulation".tw,kw. |
| 9 | "clinical frailty scale".tw,kw. |
| 10 | "Fried frailty".tw,kw. |
| 11 | "brief frailty index".tw,kw. |
| 12 | ("frail scale" or frail-scale).tw,kw. |
| 13 | "modified frailty index".tw,kw. |
| 14 | interRAI*.tw,kw. |
| 15 | ((vulnerable adj (elders* adj survey*)) or VES-13).tw,kw. |
| 16 | "Edmonton Frail Scale".tw,kw. |
| 17 | "frailty staging system".tw,kw. |
| 18 | "Conselice Study of Brain Aging Score".tw,kw. |
| 19 | ("geriatric screening tool" or G-8).tw,kw. |
| 20 | "multidimensional prognostic index".tw,kw. |
| 21 | "kihon checklist".tw,kw. |
| 22 | (("Cardiovascular Health Study" or CHS) adj2 criteria).tw,kw. |
| 23 | ("hospital frailty risk score" or HFRS).tw,kw. |
| 24 | ("electronic frailty index" or eFI).tw,kw. |
| 25 | "geriatric assessment".tw,kw. |
| 26 | or/1-25 |
| 27 | Hospitalization/ |
| 28 | Hospital patient/ |
| 29 | (in#patient or in#hospital or hospital*).tw,kw. |
| 30 | (admitted or admission*).tw,kw. |
| 31 | or/27-30 |
| 32 | Prevalence/ or prevalence.tw,kw. |
| 33 | outcome*.tw,kw. |
| 34 | Hospital mortality/ |
| 35 | mortality.tw,kw. |
| 36 | death*.tw,kw. |
| 37 | Survival analysis/ or Proportional hazards/ |
| 38 | surviv$.tw,kw. |
| 39 | Patient Readmission/ |
| 40 | (readmission* or re-admission* or readmitted or re-admitted).tw,kw. |
| 41 | (re-hospital* or rehospital*).tw,kw. |
| 42 | return.tw,kw. |
| 43 | (re-attend* or reattend*).tw,kw. |
| 44 | (re-visit* or revisit*).tw,kw. |
| 45 | recidivism.tw,kw. |
| 46 | exp Length of Stay/ |
| 47 | ((length or duration) adj3 (stay or hospital or admission*)).tw,kw. |
| 48 | or/32-47 |
| 49 | (epidemiolog* adj (study or studies)).mp. |
| 50 | exp cohort analysis/ |
| 51 | exp Case control studies/ |
| 52 | cohort.mp. |
| 53 | "case control".mp. |
| 54 | (observational adj (study or studies)).mp. |

| | |
|---|---|
| 55 | ((Follow-up or "follow up") adj (study or studies)).mp. |
| 56 | (prospective or retrospective).mp. |
| 57 | or/49-56 |
| 58 | 26 and 31 and 48 and 57 |

**Table S3. Search strategy for CINAHL** (no start date available)

| # | Searches |
|---|---|
| 1 | (MH "Frailty Syndrome") OR "frail" OR debilit* OR ( TX (frail* W3 (assessment or index or scale or phenotype or indicator or risk or score or framework or tool or screen* or syndrome)) ) OR (MH "Geriatric Assessment") OR (MH "Geriatric Functional Assessment") OR (MH "Patient Assessment") OR (MH "Clinical Assessment Tools/CL/SN/MO") OR (MH "Functional Assessment") |
| 2 | ("physical frailty phenotype" OR "deficit accumulation" OR "clinical frailty scale" OR "Fried frailty"OR "brief frailty index" OR "frail scale" OR frail-scale OR "modified frailty index" OR interRAI OR (vulnerable W1 (elders* W1 survey*) or VES-13) OR "Edmonton Frail Scale" OR "frailty staging system" OR "Conselice Study of Brain Aging Score" OR "geriatric screening tool" OR G-8 OR "multidimensional prognostic index" OR "kihon checklist" OR (("Cardiovascular Health Study" or CHS) N2 criteria) OR "hospital frailty risk score" OR HFRS OR "electronic frailty index" OR eFI OR "geriatric assessment") |
| 3 | 1 OR 2 |
| 4 | ( (MH "Hospitalization") OR "hospitalization" OR (MH "Inpatients") OR (MH "Aged, Hospitalized") ) OR ( (in#patient or in#hospital or hospital*) ) OR ( (admitted or admission*) ) |
| 5 | (MH "Prevalence") OR "Prevalence" |
| 6 | (MH "Treatment Outcomes") OR "Outcomes" |
| 7 | (MH "Hospital Mortality") OR (MH "Fatal Outcome") OR mortality OR death OR (MH "Survival Analysis") OR surviv$ |
| 8 | (MH "Readmission") OR ( readmission* or re-admission* or readmitted or re-admitted ) OR ( re-hospital* or rehospital* ) OR ( return or re-attend* or reattend or re-visit* or revisit* or recidivism) |
| 9 | (MH "Length of Stay") OR (length or duration) adj3 (stay or hospital or admission*) |
| 10 | 5 OR 6 OR 7 OR 8 OR 9 |
| 11 | (MH "Prospective Studies+") |
| 12 | (MH "Epidemiological Research") OR (MH "Prospective Studies+") OR (MH "Case Control Studies+") |
| 13 | TX ( Cohort OR prospective OR retrospective OR Longitudinal ) OR TX ( Case-control OR case control ) OR TX ( Follow-up or "follow up" W2 (study or studies) ) OR TX ( observational W2 (study or studies) ) |
| 14 | 11 OR 12 OR 13 |
| 15 | 3 AND 4 AND 10 AND 14 [*Exclude Medline results filter applied] |

## Supplementary Methods

### Eligibility criteria for frailty tools

Eligible frailty tools included the Fried phenotype model,[1] deficit accumulation model,[2] clinical measures (including multidimensional tools assessing a wide variety of domains and brief tools with $\leq$5-6 items),[3,4] global assessment (such as the Clinical Frailty Scale),[5] measures adapted from them, or comprehensive geriatric assessment (CGA).[6]

### Potential bias related to the definition of the denominator

For the outcome of long length of stay (LOS), we included people who died in hospital in the main analysis. Consequently, Forti and colleagues (2014) was excluded from the main analysis because the study excluded people who died in hospital.[7]

For the outcomes of discharge destination and readmission, we presented data using people who survived until discharge as the denominator in the main analyses. For discharge destination, we subtracted in-hospital deaths from the denominator in Bonjour and colleagues (2021) and Eckart and colleagues (2019).[8,9] Warnier and colleagues (2017) and Ramdass and colleagues (2018) excluded people who died in hospital from their studies.[10,11]

For readmissions, Warnier and colleagues (2017), Fitriana and colleagues (2021) and Laura and colleagues (2022) excluded people who died in hospital from their studies.[10,12,13] Anani and colleagues (2020) and Wallis and colleagues (2015) were assumed to exclude people who died in hospital.[14,15] For Bonjour and colleagues (2021) it was not possible to exclude inpatient deaths from the denominator used for readmissions, so the study was excluded from main analyses.[8] For Subramanian and colleagues (2020), Eckart and colleagues (2019), Street and colleagues (2021), Lujic and colleagues (2022) and Gilbert and colleagues (2022) inpatient deaths were subtracted from the overall denominators reported in the study.[9,16-19] For McAlister and colleagues (2019), we calculated the denominator from the number of readmissions reported and % of participants alive and discharge who were readmitted.[20] For Gilbert and colleagues (2018), the denominator was calculated by multiplying the number of 30-day deaths after the date of admission (including in-hospital deaths) for each frailty category by the % of overall deaths that occurred in hospital.[21]

In the supplementary material, we have included analyses using the denominators reported by study authors.

**Modified Joanna Briggs Institute tools for risk of bias assessment**

A list of items included in Joanna Briggs Institute (JBI) Checklists for prevalence studies and cohort studies are provided,[22,23] along with specific considerations for this review.

Guidance for scoring is provided in the table below. Each item will be answered as "yes," "no," "not applicable" or "unclear," however, the overall assessment will be made holistically. A study providing an estimate for the prevalence of frailty or its association with outcomes that is **very likely, likely, or unlikely** to be correct for the author's stated target population will be assigned a **low, moderate or high** risk of bias, respectively. In general, a study at a low risk of bias will meet or nearly meet all criteria, whereas a study could be assigned a high risk of bias based on one or more serious flaws in the study design. A study at a moderate risk of bias will be in between—not meeting all criteria, but not having serious methodological concerns either. Additionally, an assessor may also describe an entire study as uncertain if there is insufficient information.

**Table S4. Modified Joanna Briggs Institute Checklist for Prevalence Studies**

| Item | | Scoring Guidance |
|---|---|---|
| **1** | Was the sample frame appropriate to address the target population and was sufficient coverage of the sample frame achieved? (Original items #1 & 5) | **Yes:** The prevalence of frailty is likely to be similar between the sample frame and target population identified by the authors. This may be inferred from participant characteristics, and eligibility criteria **AND** there should also be no important differences in age, sex, or other factors between participants and eligible non-participants.<br><br>**No:** The prevalence of frailty is unlikely to be similar between the target population and the sample frame **OR** the characteristics of participants and eligible non-participants are different such that the prevalence of frailty is unlikely to be similar between groups.<br><br>Also see: NIHR (2020) Improving inclusion of under-served groups in clinical research: Guidance from the NIHR-INCLUDE project. UK: NIHR. Available at: www.nihr.ac.uk/documents/improving-inclusion-of-under-served-groups-in-clinical-research-guidance-from-include-project/25435 (date link accessed) |
| **2** | Were study participants recruited appropriately? | **Yes:** The study uses random sampling, consecutive enrollment or near-complete (≥90%) ascertainment of the target population.<br><br>**No:** Convenience sampling is used. |
| **3** | Was the sample size adequate? | **Yes:** The sample size was ≥384 or a smaller sample size with at least 80% power was calculated and justified by the authors.<br><br>The minimum sample size was estimated using the method described in the JBI manual and published statistical guidance:[23,24]<br><br>$Sample\ size = \frac{Z^2P(1-P)}{d^2}$<br><br>Where:<br>$Z$ = Z statistic for a level of confidence (95% level)<br>$P$ = Expected prevalence (50%)<br>$d$ = Desired precision (5%)<br><br>An expected prevalence of 50% was selected based on the median frailty reported in a scoping review of frailty prevalence in acute care and general medicine settings.[25]<br><br>**No:** The sample size was <384. |
| **4** | Were the study subjects and setting described in detail? | **Yes:** The distribution of age, sex, co-morbidities and other characteristics was reported, such that it is clear who the study results apply to.<br><br>**No:** Age, sex or co-morbidities were not reported. |

| | | |
|---|---|---|
| **5** | Were valid methods used for the identification of the condition? | **Yes:** The authors reference an appropriate validation study or evaluated the performance of the frailty measure used against the frailty evaluated as part of Comprehensive Geriatric Assessment. The method of frailty measurement appears to have reasonable internal, external, construct and conclusion validity and reliability.<br><br>**No:** No validation study is referenced, or the performance of the frailty measure was not evaluated. The method of frailty measurement does not appear to have reasonable internal, external, construct and conclusion validity and reliability. |
| **6** | Was the condition measured in a standard, reliable way for all participants? | **Yes:** If different frailty assessment methods, settings, or assessors were used, the Kappa value was at least 0.80 or stratified data was reported.<br><br>**No:** The prevalence of frailty is unlikely to be similar for all participants, because of the differences listed above. |
| **7** | Was the response rate adequate, and if not, was the low response rate managed appropriately? | **Yes:** The number of participants analyzed was not substantially reduced (<20%) by attrition or missing data. Reasons for withdrawal are reported, and characteristics of participants who completed the study are similar to those who did not. For data that is not missing at random, multiple imputation or other appropriate methods were used.<br><br>**No:** The number of participants analyzed was substantially reduced (>20%) by attrition or missing data. There were important differences between those who withdrew and those who completed the study. Data that is missing not at random was managed with listwise deletion or complete cases analysis. |

## Table S5. Modified Joanna Briggs Institute Checklist for Cohort Studies

| **Item** | | **Scoring Guidance** |
|---|---|---|
| **1** | Were the two groups similar and recruited from the same population? | **Yes:** The two groups had similar characteristics, except for the exposure and were recruited from the same population. This may be inferred from participant characteristics, and eligibility criteria.<br><br>**No:** The two groups had substantially different characteristics, except for the exposure, and/or were recruited from different populations. |
| **2** | Were the exposures measured similarly to assign people to both exposed and unexposed groups? | **Yes:** Frailty was measured similarly between groups and described in detail.<br><br>**No:** Frailty was not measured similarly between groups. |
| **3** | Was the exposure measured in a valid and reliable way? | **Yes:** The study clearly described the method for measuring frailty, which appeared reliable (e.g. measure used, timing of assessment and training of assessor or a measure of reliability was reported). The method for assessing frailty was valid (see item #6-7 in the modified Checklist for Prevalence Studies).<br><br>**No:** The method for measuring frailty did not appear to be reliable or valid. |
| **4** | Were confounding factors identified and handled appropriately? | **Yes:** There does not appear to be important confounding, **OR** confounding factors were identified and controlled for (e.g. multivariable regression, standardization, or stratification). A minimum of age, sex, co-morbidity and illness acuity were adjusted for.<br><br>**No:** Confounding factors (including a minimum of age, sex, and co-morbidity) were not identified or controlled for. Ideally, illness severity will be controlled for as well. |

| | | |
|---|---|---|
| **5** | **For the outcome, “discharge to a location other than home” ONLY:**<br><br>Were the groups/participants free of the outcome at the start of the study (or at the moment of exposure)? | **Yes:** The participants were free of the outcome (i.e. living in a care home or residential facility) at the start of the study/moment of exposure **OR** the study only counted discharge to a *new* care home or residential facility as an event.<br><br>**No:** No, some participants were living in a care home or residential facility at the start of the study and this was not taken into account in evaluating the outcome. |
| **6** | Were the outcomes measured in a valid and reliable way? | **Yes:** Outcomes were assessed in the same way for all participants and the method was valid. In general, this criterion should be satisfied, for example, if the outcome is mortality and it was assessed using a national registry.<br><br>**No:** Outcomes were not assessed in the same way for all participants, or the method used was not valid. |
| **7** | Was the follow up time reported and sufficient to be long enough for outcomes to occur? If the length of follow-up differed between groups, were appropriate strategies used to address it? (Original items #8 & 10) | **Yes:** Follow-up time was sufficient for the outcomes reported **OR** if substantially different between groups, the difference in follow-up time was accounted for in the analysis, for example by using person-time at risk.<br><br>**No:** Follow-up time was not sufficient **AND** not accounted for in the analysis. For example, if a study reported in-patient mortality only, then deaths related to the reason for hospitalization that occurred after discharge would not be counted and could introduce bias. |
| **8** | Was follow up complete, and if not, were the reasons to loss to follow up described and explored? | **Yes:** The number of participants analyzed was not substantially reduced (<20%) by loss to follow up. Reasons for loss to follow up were described, and characteristics of participants who were lost to follow up were reported and were similar to those who were not.<br><br>**No:** The number of participants analyzed was substantially reduced (>20%) by loss to follow up. There were important differences between those who were lost to follow up and those who were not**.** |

## Statistical reporting

| | | |
|---|---|---|
| **1** | Was there appropriate statistical analysis for all relevant outcomes? | **Yes:** Sufficient information are provided to reproduce the analysis, including methods used to adjust for confounding variables. Confidence intervals or a comparable measure of uncertainty are reported or can be calculated.<br><br>**No:** Insufficient information were reported to reproduce the analysis and confidence intervals were not reported/could not be calculated. |

**Cut-points selected for methods of frailty ascertainment with more than one frailty category**

**Table S6. Ordinal frailty cut-points for data synthesis and meta-analysis.** Values represent acceptable ranges.

| Assessment tool | Mild or no frailty | Moderate frailty | Severe frailty | Reference |
|---|---|---|---|---|
| **9-point CFS** | 0-5 | 6 | 7-9 | 5 |
| **BISEP** | 0-1 | 2 | ≥3 | 26 |
| **eFI/eFI-AH** | 0-0.24 | >0.24-0.36 | >0.36 | 27 |
| **FCS-1** | Risk strata 1 | Risk strata 2 | Risk strata 3 | 28 |
| **FI-CGA** | <0.45 | 0.45-0.55 | >0.55 | 29 |
| **FRAIL** | 0 | 1-2 (Pre-frail) | 3-5 | 30 |
| **HFRS** | <5 | 5-15 | >15 | 21 |
| **ISAR-HP** | 0-1 | 2-3 | 4-5 | 31 |
| **MAPLe-AC** | Low or mild | Moderate | High or very high | 32 |
| **MPI** | <33 (Group 1) | 33-66 (Group 2) | >66 (Group 3) | 33 |
| **REFS/EFS** | 0-9 | 10-11 | 11-18 | 34 |

**Table S7. Dichotomous frailty cut-points for data synthesis and meta-analysis.** Values represent acceptable ranges.

| Assessment tool | Fit or mildly frail | Moderate-to-severely frail | Reference |
|---|---|---|---|
| **7-point CFS** | 1-5 | 6-7 | 5 |
| **9-point CFS** | 1-5 | 6-9 | 5 |
| **FI (Eeles et al., 2015)** | <0.25 | ≥0.25 | 35 |
| **FRAIL** | 0-2 | 3-5 | 30 |
| **Fried** | 0-2, or 0-3 | 3-5, or 4-5 | 1 |
| **HFRS** | <5 | ≥5 | 21 |
| **ISAR-HP** | <2 | ≥2 | 31 |
| **REFS/EFS** | Not frail | Vulnerable-Severe | 34 |
| **SOF-I** | 0-1 | ≥2 | 36 |
| | Mild or no frailty ordinal category | Sum of moderate and severe ordinal categories | |

**Note:** Tools with uncertain cut-offs included the Frail-PPS, FAM and method used by Soong et al., 2015 and so estimates were not included in main analyses.

**Supplemental results - tables**

**Table S8. Summary of characteristics of included studies and prevalence estimate**s (data is not weighted by sample size).

| **Characteristic, n, % or range** | **Setting All (n=45)** | **Hospital-wide (n=17)** | **General medicine (n=28)** |
|---|---|---|---|
| **n cohorts** | | | |
| Participant characteristics | | | |
| Median age years (range), n=42 | 64-87 | 74-85 | 64-86 |
| 55-65 | 1 | 0 | 1 |
| 65-75 | 3 | 1 | 2 |
| 75-85 | 29 | 11 | 18 |
| >85 | 4 | 2 | 2 |
| Female, % (range), n=41 | 38-72 | 50-60 | 38-65 |
| Cognitive impairment, % (range), n=24 | 1-68 | 7-51 | 1-68 |
| Median CCI (range), n=14 | 2-8 | 2-3 | 2-8 |
| Social context, n=8 | | | |
| Living alone, % (range) | 12-61 | 29-39 | 12-61 |
| Living with others, % (range) | 48-62 | 57-62 | 48-52 |
| Living situation, n=13* | | | |
| At home, with assistance or independently, % (range) | 68-97 | 72-96 | 58-97 |
| Residential facility, % (range) | 4-34 | 4-11 | 5-34 |
| | | | |
| **n estimates (n=54)** | | | |
| Frailty ascertainment | | | |
| Included non-physical domains | | | |
| Cognitive (not including delirium) | 29 | 14 | 15 |
| Social supports | 18 | 12 | 6 |
| | | | |
| Type of frailty tool used | | | |
| Brief screening tool | 5 | 3 | 2 |
| Clinical frailty scale | 10 | 2 | 8 |
| Deficit accumulation | 18 | 10 | 8 |
| Fried phenotype | 9 | 2 | 7 |
| Multiple domains based on the CGA (‘multi-domain’) | 7 | 2 | 5 |
| Other | 5 | 1 | 2 |

CCI = Charlson Comorbidity Index. *4 additional studies excluded participants from care homes. **Some studies had multiple estimates available (e.g., measured frailty with multiple tools).

**Table S9. Summary of included cohorts from studies of all hospital admissions** (n=17 cohorts with prevalence estimates)

| Author & Year (Study aims) | Location, enrollment dates, sample size | Age, % female | Comorbidity, cognitive impairment | Exclusion criteria | Frailty assessment | Outcomes |
|---|---|---|---|---|---|---|
| Asmus-Szepesi et al., 2013[37] (P) | Netherlands (regional, 450 bed hospital) 2010<br><br>n=460 | 76 (7.2) years<br>56% F | 73% (>1 comorbidity)<br>51% (4% severe) (MMSE, cut-off value not reported) | Age <65 years, refused participation, terminally ill, unable to follow instructions, included in previous admission, LOS <48 hours, not contacted before discharge | ISAR-HP | Mortality, ADLs/IADLs, MMSE, Loneliness Scale, Short Form-20 (1 year) |
| Fujita et al., 2022 (C)[38] | Australia (single hospital) 2019-2020<br><br>n=6771 | 83.5 (N/A) years<br>52% F | (9% (CCI>3),<br>N/A | Age <75 years, no valid eFI-AH, first admission only, <30 eFI-AH items evaluated | eFI-AH, HFRS | Mortality, >10-day LOS, unplanned and fall-related readmission and fall-related readmission (28 days) |
| Geriatric Medicine Research Collaborative 2019 [39] (O) | England (45 acute hospitals) 2018<br><br>n=1507 | 80.0 (8.3) years<br>53% F | N/A<br>N/A | Age <65 years, critical care, imminently approaching the end of life, logistical, missing 4AT score and delirium status | 9-point CFS | N/A |
| Gilbert et al., 2018 (local validation cohort)[21] (D,V) | England (single hospital, unclear) 2013-2015<br><br>n=569 | 79.9 (6.4) years<br>56% F | 1.9 (2.1) (CCI)<br>N/A | Age <75 years | HFRS | N/A |
| Gilbert et al., 2018 (national validation cohort)[21] (D,V) | England (NHS Hospital Episode Statistics) 2014-2015<br><br>n=1013590 | 84.1 (5.9) years<br>57% F | 2.9 (2.6) (CCI)<br>26% (ICD-10 dementia/ Alzheimer's) | Age <75 years, not admitted as an emergency | HFRS | Mortality, emergency readmission (30 days), >10-day LOS |
| Gilbert et al., 2022[19] (V) | France (743 hospitals, PMSI database) 2017 | 84.9 (5.8) years | 1.5 (1.4) (CCI) | Age <75 years, not hospitalised as an emergency, | HFRS | Mortality, LOS > 10 days and 30-day emergency readmissions (30 days) |

| | | | | | | |
|---|---|---|---|---|---|---|
| | n=1,042,234 | 80% F | | first admission only, missing socioeconomic data | | |
| Hollinghurst et al., 2021[40] (C, P) | Wales (multiple hospitals, SAIL Databank) 2013-2017<br><br>N=126600 | 79.3 (8.4) years<br>53% F | N/A<br>N/A | Age <65 years, emergency admission, registered with a GP contributing to SAIL, residential history in Wales | HFRS, eFI | Mortality (inpatient, 90 days), >10day LOS, readmission (30, 90 days), care home admission (1 year) |
| Lim et al., 2023[41] (C, V) | Singapore (tertiary hospital)<br><br>n=366 | 74 years<br>56% F | 2.9 (chronic health conditions) | Age <65 years, admitted to ICU, transferred from another hospital, diagnosed with a terminal illness, cognitive impairment/dementia, admitted for a stroke or had LOS<48h | Frail-PPS, FAM, ISAR-HP | N/A |
| Lujic et al., 2022[18] (O) | Australia (New South Wales Admitted Patient Data) 2008-2013<br><br>n=257,535 | 83 years<br>57% F | 48% (2+ conditions) | Age <75 years | HFRS | Mortality, LOS >10 days, unplanned readmission (30 days) |
| McAlister et al., 2018[20] (C, P) | Ontario, Canada (CIHI Discharge Abstract Database**) 2004-2010<br><br>n=452785 | 83 (5.6) years<br>60% F | 2 (2.2) (CCI)<br>N/A | Age <75 years, psychiatric admissions, not urgent admission | HFRS | Mortality, urgent readmission, >10 day LOS, post-discharge ED visits (30 days) |
| Romero-Ortuno et al., 2016a,[42] Romero-Ortuno et al., 2016b[43] (P) | England (large tertiary university hospital with 1000 acute beds) 2014-2015<br><br>n=5899 | 84.1 (5.9) years<br>56% F | N/A<br>14% (hx dementia) | Age <75 years, elective admission | 9-point CFS | Mortality (inpatient), readmission (30 days), >10 day LOS, discharge to care home, delayed discharge |
| Soong et al., 2015[44] (Prev) | England (NHS Hospital Episode Statistics) 2005-2013<br><br>n=50540141 (prevalence for approximately n=34044050) | N/A<br>50% F | 7% (ICD-10, dementia) | Age <65 years | Author-designed | N/A |

| | | | | | | |
|---|---|---|---|---|---|---|
| Soong et al., 2019* (development cohort)[45] (Prev, P, V) | Global Comparators Database (34 hospitals in Australia, Belgium, Denmark, Finland, Italy, Netherlands, Norway, United Kingdom, USA) 2010-2014<br><br>n=1366187 | N/A<br>72% F | N/A<br>16% (ICD-9/10 dementia/delirium) | Age <75 years, missing age/sex/LOS, LOS <2 days | Dr Foster GFS | Mortality (inpatient), non-elective readmission (30 days), LOS in upper quartile for country |
| Street et al., 2021[17] (O) | Yorkshire and Humber regions, England (13 acute NHS hospitals/49 urban, suburban and rural sites, CUREd database) 2013-2017<br><br>n=282091 patients, 675155 admissions | N/A<br>N/A | 2.0 (1.9) (CCI)<br>N/A | Age <75 years, emergency admission. | HFRS | Mortality (inpatient), readmission (30 days), >10 day LOS |
| Timmons et al., 2015[46] (O) | Cork County, Ireland (three urban public hospitals, two rural public hospitals, and one private hospital) 2012-2013<br><br>n=598 | 79.7 (6.6) years<br>51% F | N/A<br>25% (SMMSE<27/30, IQCODE, health records, DSM-IV, expert review) | Age <70 years, day-cases, moribund on admission, refused participation | SHARE-FI | N/A |
| Wallis et al., 2015[15] (P) | England (tertiary/university hospital) 2013-2014<br><br>n=5764 | 84.6 (5.9) years<br>56% F | N/A<br>24% (hx dementia) | Age <75 years, not admitted as an emergency | 9-point CFS | Mortality (inpatient), readmission (30 days), >10 day LOS, mean LOS, transfer to geriatric ward |
| Warnier et al., 2017,[10] Warnier et al., 2019[47] (P) | Maastricht University Medical Center+, Maastricht, Netherlands (university hospital with 715 beds) 2013<br><br>n=2691 | 78.9 (6.4) years<br>51% F | N/A<br>N/A | Age <70 years, not community-dwelling, not admitted to a regular/general ward, or LOS <48 hours | MFST-HP | Mortality, readmission (30 days, 120 days), mean LOS, discharge to care facility, |

C=compare existing tools, D=develop a new tool, P=investigate prognosis, Prev=describe frailty prevalence, V=validate a tool, O=other**.** SAIL=Secure Anonymised Information Linkage Databank; CUREd=The Connected Health Cities Urgent and Emergency Care Research database. A sMMSE<27 is suggestive of dementia or mild cognitive impairment, but additional criteria must be met to formally diagnose dementia based on the DSM-V (i.e. cognitive deficits interfere with daily activities and are not explained by delirium or another mental condition). *****Prevalence estimate only available for development cohort, so the validation cohort was not included in the summary descriptive measures; ** Linked to Registered Person's Database, National Ambulatory Care Reporting System, Continuing Care Reporting System.

**Table S10. Summary of included cohorts from studies of general medicine admissions** (n=28 cohorts)

| **Author & Year (Study aims)** | **Location, enrollment dates, sample size** | **Age, % female** | **Comorbidity, cognitive impairment** | **Admission diagnoses, 3 most common (%)** | **Exclusion criteria** | **Frailty assessment** | **Outcomes (follow-up)** |
|---|---|---|---|---|---|---|---|
| Anani et al., 2020[14] (P) | Israel (Internal medicine department at a tertiary care hospital) (dates not reported)<br><br>n=980 | 72 (65-79) years<br>43% F | N/A<br>1% (dementia, unspecified) | N/A<br>N/A<br>N/A | Age <55 or >85 years, no consent, advanced malignancy, cirrhosis, dementia, COPD or CHF, hx major stroke, admitted for diabetic foot or severe PVD, bedridden, mechanical ventilation on admission, or hospitalized <30 days ago, not admitted from emergency department | FRAIL | Readmission (30 days) |
| Belga et al., 2016[48], Kahlon et al., 2015,[49] McAlister et al., 2019[50] (C, P) | University of Alberta and Royal Alexandra hospitals, Edmonton, Canada (General internal medicine wards at teaching hospitals (n=2)) (2013-2014)<br><br>n=495 | 64 years<br>51% F | 2.4 (CCI)<br>N/A | 1. Circulatory (10%),<br>2. Respiratory (8%),<br>3. Infection (5%) | Age <18 years, life expectancy <3 months, admitted from or discharged back to long-term care facilities or other hospitals, out-of-province, poor English, moderate-severe cognitive impairment | 9-point CFS, Fried, HFRS | Comparison with other risk measures, LOS, readmission, mortality, composite of readmission & mortality, emergency department visit (30 days) |
| Bonjour et al., 2021[8] (Pr, P) | CHUV, Lausanne, Switzerland (Internal medicine unit at university hospital) (2009-2017)<br><br>n=22,323 | 80.2 (8.2) years<br>52% F | 2 (0-4) (CCI)<br>10% (delirium, ICD-9 codes) | 1. Other (65.4%),<br>2. Circulatory (12.4%),<br>3. Infection (>10.8%) | Age <65 years | HFRS | LOS, ICU stay, discharge destination, mortality (30, 90 days, 1 year), readmission rate, costs, DRG category |
| Buurman et al., 2012[51] (DEFENCE cohort) (P, O) | Academic Medical Center, Amsterdam; University Medical Centre Utrecht, Utrecht; and Spaarne Hospital, Hoofddorp, Netherlands (General medical wards at tertiary and regional hospitals with >1000 beds (n=2), and 455 beds (n=1), respectively) (2006-2008)<br><br>n=639 | 78.2 (7.8) years<br>54% F | 3.5 (2.3) (CCI)<br>40% (MMSE≤15 and CGA during admission) | 1. Infection (41%),<br>2. Circulatory (4%)<br>N/A | Age <65 years, patient/relative did not consent, too ill to participate, transferred from other ward, >48 hours after admission, transferred to ICU, CCU or other ward within 48 hours of admission, or poor Dutch | ISAR-HP | Clinical characteristics, mortality, functional decline (3 months and 1 year) |

| | | | | | | | |
|---|---|---|---|---|---|---|---|
| Dani et al., 2017[52] (O) | London, England (Acute medical unit at an urban general hospital ) (2007-2007)<br><br>n=710 | 83.1 (7.4) years<br>59% F | N/A<br>42% (dementia, DSM-IV) | N/A<br>N/A<br>N/A | Age <70 years, LOS <48 hours, poor English | FI | Mortality in the absence or presence of delirium (3 years) |
| Eckart et al., 2019[9] (TRIAGE cohort) (P, V) | Kantonsspital, Aarau, Switzerland (Medical admissions to a tertiary care hospital) (2015-2018)<br><br>n=4957 | 82 (75-85) years<br>51% F | N/A<br>7% (dementia, ICD-10) | N/A<br>N/A<br>N/A | Age <75 years, non-urgent admission | HFRS | Discharged home (with or without help) or assisted living, all-cause mortality, readmission (30 days) |
| Eeles et al., 2012[35] (P, O) | South Wales, UK (General medical service at a district general hospital)<br><br>n=273 | 82.3 (7.5) years<br>N/A | N/A<br>N/A | N/A<br>N/A<br>N/A | Age <75 years, patient/proxy did not consent, readmission | FI | Mortality (5 years) |
| El-Sharkaway et al., 2005[53] (O) | Queens Medical Centre Nottingham, England (Medical wards) (2012-2014)<br><br>n=187 | 81.6 (7.4) years<br>47% F | 4 (3-5) (CCI)*<br>24.5 (6.2) (mean/SD MMSE, cut-point unclear) | N/A<br>N/A<br>N/A | Age <65 years, terminal illness and life expectancy <3 months | CFS | Hydration status in-hospital |
| Evans et al., 2014[29] (P, V) | Mercy Hospital, Buffalo, New York, USA (General medicine) (2010-2011)<br><br>n=751 | 84 (5.5) years<br>64% F | 7.5 (2.3) (CCI)<br>29% (hx dementia) | N/A<br>N/A<br>N/A | Age <75 years, over 24 hours passed since admission | FI-CGA | Mortality, discharge to nursing home, rehab or assisted living (120 days), time-to-complete assessment |
| Fitriana [12] et al., 2021 (P) | Cipto Mangunkusumo Hospital, Jakarta, Indonesia (Acute care ward) (2019)<br><br>N=266 | N/A<br>N/A | N/A<br>26% (AMT<8) | N/A<br>N/A<br>N/A | Age<60 years, died in hospital, transferred to other hospital | FRAIL | Readmission (30 days) |

| | | | | | | | |
|---|---|---|---|---|---|---|---|
| Forti et al., 2014[7] (P) | S.Orsola-Malpighi University Hospital, Bologna, Italy (General internal medicine unit at a university hospital) (2012)<br><br>n=470 | 80.8 (7.5) years<br>53% F | 66% (CCI>/=2)<br>52% (hx cognitive impairment) | 1. Stroke (17%),<br>2. Respiratory (15%),<br>3. Digestive (8%) | Age <65 years, died, terminal illness or coma, discharged/transferred within 48 hours of admission, refused participation or incomplete data | SOF-I | Composite of mortality and discharge location other than previous residence, ward LOS greater than median (i.e. >8 days) |
| Gregoravic et al., 2016[54] (P) | St Vincent's Hospital, Melbourne, Australia (General medical unit at an inner-city tertiary care univer sity-associated hospital) (2013)<br><br>n=170 | 82 years<br>49% F | 6.6 (CCI)<br>N/A | N/A<br>N/A<br>N/A | Age < 65 years, transferred to a specialty unit | 9-point CFS | Mortality, functional decline (3 months) |
| Hernandez-Luis et al., 2018[55] (O) | Canary Islands University Hospital, Tenerife, Spain (Internal medicine department at a teaching hospital) (2013-2015)<br><br>n=298 | 76.6 (61-98) years<br>53% F | 36% (CCI >3)<br>11.7% (dementia, unspecified) | 1. Sepsis (37%),<br>2. Pneumonia (24%),<br>3. UTI (18%) | Age <61 years, life expectancy <6 months, acute delirium or impaired consciousness persisting on second day of admission, not admitted from emergency department | 7-point CFS, Fried | Mortality, rapid cognitive decline (100 days) |
| Hoogerduijn et al., 2012[31] (P) | The Netherlands (Internal medicine wards at a teaching hospital) (2002-2006)<br><br>n=492 | 78 years<br>56% F | N/A.<br>34% (MMSE <24) | 1. Infection (9%),<br>2. Circulatory (1%)<br>N/A | Age <65 years, too ill to participate | ISAR-HP | Functional decline (3 months) |
| Inouye et al., 2003[26] (D, V) | Yale New Haven Hospital, New Haven, USA (Medicine service at a teaching hospital with 800 beds)<br><br>n=535 | 78.7 (6.1) years<br>56% F | 71% (CCI ≧2)<br>27% (dementia, unspecified) | 1. Infection (13%),<br>2. Circulatory (12%),<br>3. Malignancy (2%) | Age < 70 years, terminal condition, severe dementia, aphasia, coma, intubation, discharge within 48 hours, prior enrollment, refused participation | BISEP | Comparison with other illness measures, mortality (1 year) |
| Irina et al., 2018[56] (O) | Chaim Sheba Medical Center, Ramat Gan, Israel (Internal medicine department at a large tertiary care hospital)<br><br>n=179 | 72 (65-79) years<br>46% F | N/A<br>N/A | N/A<br>N/A<br>N/A | Age <18 years, dementia/cognitive decline, admission ALT >40 IU/L, no follow-up data, unable to complete frailty assessment | FRAIL | Mortality, correlation with serum ALT (maximum 456 days) |

| | | | | | | | |
|---|---|---|---|---|---|---|---|
| Juma et al., 2016[57] (P) | Victoria Hospital, London, Canada (Clinical teaching unit)<br><br>n=75 | 81.4 (8.8) years<br>64% F | 6.2 (2.1) (Mean comorbidities)<br>N/A | N/A<br>N/A<br>N/A | Age <65 years, palliative designation or life expectancy <7 days, no chronic health conditions, independent ambulation at baseline | 9-point CFS | Readmission (90 days) |
| Khandelwel et al., 2012[58] (Prev, P, O) | India (Medical wards at a public teaching hospital)<br><br>n=250 | 66.4 (6.3) years<br>38% F | N/A<br>25.79 (4.04) (mean/SD MMSE) | 1. Infection (31%),<br>2. Circulatory (20%),<br>3. Respiratory (15%) | Age <60 years, requiring mechanical ventilation/life support, comatose, obvious neurologic deficits | Fried | Mortality (45 days) |
| Laura et al., 2012[13] (O) | Singapore (General medicine department at hospital) (2018-2020)<br><br>n=1619* | 76.4 (7.9) years<br>51% F | 60% (CCI>=3)<br>27% (dementia or AMTS<8), 10% delirium-CAM | N/A<br>N/A<br>N/A | Age <65 years, admitted from shelters or nursing homes, via HDU/ICU or non-medical units; died in hospital, transferred, or discharged against medical advice, did not consent. | 9-point CFS | Readmission (30 days) |
| McCrow et al., 2016[59] (O) | Queensland, Australia (Internal medicine unit at a large tertiary care hospital) (2012-2013)<br><br>n=44 | 81 (8.5) years<br>55% F | 1.93 (1) (CCI)<br>61% (RUDAS ≧23) | N/A<br>N/A<br>N/A | Age <60 years, unstable CHF, severe CKD, nil by mouth on admission, expected LOS <24 hours, baseline assessment not completed within 24 hours of admission, poor English | 7-point CFS | Association with dehydration and cognitive impairment |
| Nardi et al., 2019[60] (P, V) | Italy (Internal medicine departments (n=29)) (2014)<br><br>n=541 | 79.9 (12) years<br>51% F | N/A<br>N/A | N/A<br>N/A<br>N/A | Age <40 years, fewer than two chronic diseases | MPI | Mortality (1 year), time-to-complete assessment |
| Noro et al., 2011[61] (D, P) | Nordic countries (Acute medical care in hospitals with a catchment of >90 000 persons (multiple))<br><br>n=763 | N/A<br>65% F | N/A<br>N/A | N/A<br>N/A<br>N/A | Age <75 years, in critical condition/admitted to critical care | FCS-1 | Mortality, institutionalization, composite measure (at discharge and 1 year) |

| | | | | | | | |
|---|---|---|---|---|---|---|---|
| Polidoro et al., 2013[62] (O) | Santa Maria Goretti Hospital, Latina, Italy (Internal medicine unit at a university hospital)<br><br>n=140 | 79.3 (7.4) years<br>60% F | N/A<br>62% (MMSE<24) | N/A<br>N/A<br>N/A | Not reported | MAPLe-AC | Association with atrial fibrillation |
| Ramdass et al., 2018[63] (P) | Baystate Medical Center, Springfield, USA (Medical service) (2014-2016)<br><br>n=503 | 80 (75-86) years<br>54% F | 1 (0-4) (GCI)*<br>52 % (Mini-Cog<3) | 1. Infection (22%),<br>2. Respiratory (20%),<br>3. Circulatory (19%) | Age <65 years, refused participation, died during hospitalization, advanced dementia, transferred from other facility, admitted under observation, not living in community | REFS | Discharge to skilled nursing or inpatient rehab facility |
| Rizza et al., 2021[64] (V) | Italy (internal medicine department)<br><br>n=80 | 82.3 (4.4) years<br>46% | | | Age <75 years, unable to complete CGA, no consent, end-stage cancer or CKD, connective tissue or inflammatory bowel diseases, sepsis | MPI | N/A |
| Rose et al., 2014[65] (Prev, P) | Melbourne, Australia (General medical unit at a private hospital) (2012)<br><br>n=133 | 86.5 (6.1) years<br>61% F | N/A<br>68% (Clock drawing errors) | N/A<br>N/A<br>N/A | Age <70 years, unable to complete REFS, refused participation | REFS | Discharge disposition (return to previous residence, rehabilitation, geriatric evaluation and management, palliative care and death), time-to-complete assessment |
| Sharma et al., 2022[66] (O) | Flinders Medical Centre, Adelaide, Australia (medical admissions at a tertiary care hospital)<br><br>N=263 | 84.1 (6.6) years<br>52% | 5.6 (3.5) (CCI)<br>N/A | N/A<br>N/A<br>N/A | Age <65 years, no consent, terminally ill | REFS | N/A |
| Subramanian et al., 2020[16] (P, O) | Frankston Hospital, Melbourne, Australia (Acute medical unit at a metropolitan hospital) (2015)<br><br>n=1118 | N/A | 5 (range: 4-6) (CCI)<br>9% (dementia, delirium, senility, ICD-10) | 1. Injury or orthopaedic (19%),<br>2. Infection (14%),<br>3. Circulatory (10%) | Age <80 years, admitted for <24 hours | HFRS | Mortality (in-hospital), GOC/ACP, LOS (stratified by GOC), rapid response call during admission, discharge location (rehab, previous residence), readmission (28 days) |

C=compare existing tools, D=develop a new tool, P=investigate prognosis, Prev=describe frailty prevalence, V=validate a tool, O=other**.** An MMSE<24 is suggestive of dementia, whereas a MMSE≤15 is suggestive of moderate-severe dementia. An AMT<8 is suggestive of any cognitive impairment (e.g. dementia, possibly delirium). A RUDAS≤22 is suggestive of dementia, as is a Mini-Cog<3. Errors on the clock-drawing test are suggestive of dementia. Additional criteria need to be met to formally diagnose dementia based on the DSM-V (i.e. cognitive deficits interfere with daily activities and are not explained by delirium or another mental condition). *N=1507 included in analyses.

## Table S11. Summary of frailty assessment tools used in included studies

| Frailty Tool | Description of original tool | Cut points | Items (n) | Includes cognition? | Includes social supports? | Relies on retrospective coding data*? | Originally developed for hospital population?* |
|---|---|---|---|---|---|---|---|
| Burden of Illness Score for Elderly Persons (BISEP)[26] | An index scored from 0 to 7, which includes dementia, walking impairment, high-risk diagnoses, low serum albumin and high creatinine. | Risk group I (0-1), II (2), III (3), IV ( ≧ 4) | 5 | Yes (Alzheimer's, organic brain syndrome, or chronic cognitive impairment in medical record) | No | No** | Yes |
| CSHA Clinical Frailty Scale (7- or 9-point CFS)[5] | 7-point scale ranging from 1 (very fit for their age) to 7 (completely dependent, or terminally ill). | Not frail (1-3), apparently vulnerable (4), mildly frail (5), moderate-to-severely frail (6-7) | 1 | N/A | N/A | No | No |
| Developed by author (Soong et al. 2015)[44] | Frailty syndromes identified from ICD-10 diagnostic codes | Not frail (0), frail (≧ 1 or ≧ 4) | 7 | Yes (ICD-10 codes for delirium, dementia and senility) | No | Yes | Yes |
| Dr Foster Global Frailty Score[45] | Prediction model developed from administrative data. Includes demographic information, frailty syndromes, comorbidity and previous admissions. | Unclear | 11 | Yes (ICD-9/10 codes for dementia and delirium) | No | Yes | Yes |
| Dutch National Safety Management Program (VMS)[67,68] | A questionnaire assessing delirium, fall risk, malnutrition (MUST), and functional decline (Katz index). | Frail (≥3 aged 70-80 years; ≥1 over 80 years) | 6 | Yes (Self-report of "transient state of confusion") | No | No | Yes |

| | | | | | | | |
|---|---|---|---|---|---|---|---|
| Eletronic Frailty Index (eFI)[27] | Score derived from 36 equally-weighted deficits coed in primary care read codes in the electronic patients records | Fit (0-0.12); mild frailty (>0.12-0.24), moderate frailty (>0.24-0.36); severe frailty (>0.36) | 36 | Yes (read codes for 'Memory and cognitive problems') | Yes (read codes for 'Social vulnerability') | Yes | No |
| Electronic Frailty Index Acute Hospital (eFI-AH)[69] | Score consisting of 37 deficits, including dementia, delirium, pressure sore risk, falls risk, speech pathology review, polypharmacy or comorbidities from a variety of sources, including routine screening tools or administrative data. | Robust (eFI-AH < 0.05); prefrail (0.05 ≤ eFI-AH < 0.25); frail (0.25 ≤ eFI-AH) | 37 | Yes (Brief Cognitive Screen Six Item Screener | No | Yes** | No |
| FADOI-COMPLIMED Score 1[28] | Score resulting from the average of Barthel Index (ADLs) and Exton Smith (pressure sores) scores after recalibration such that the scale ranges from 0 (best) to 100 (worst). | NA | 2 | No | No | No | Yes |
| Frailty Assessment Measure (FAM)[41] | Score is a subset of Frail-PPS and includes age, sex and 12 items assessing physical frailty (i.e., body mass index, mobility limitations, chronic physical conditions and handgrip strength), age and sex. | Robust (<0.38); Pre-Frail (≥0.38-≤0.82); Frail (>0.82) | 14 | No | No | No | Unclear |
| Frail-Physical, Psychological, Social (Frail-PPS)[41] | Screening tool including physical frailty, age and sex, as well as measures of 'psychologic' and social vulnerability. | Robust (<0.41); Pre-Frail (≥0.41-≤0.83); Frail (>0.83) | 31 | Yes (10-item Short Portable Mental Status Questionnaire) | Yes (6-item Lubben social network scale) | No | No |
| FRAIL questionnaire[30] | 5-point questionnaire evaluating self-reported fatigue, resistance (ability to climb stairs), ambulation, weight loss, and ≧5/11 illnesses. | Robust (0 criteria), pre-frail (1-2), frail (3-5) | 5 | No | No | No | No |

| | | | | | | | |
|---|---|---|---|---|---|---|---|
| Frailty Index; Searle et al., 2008[2] | 40-item index including deficits that are associated with health status, generally increase in prevalence with age, and do not saturate before old age, for example, presbyopia. | N/A, continuous | At least 30-40 | Yes (MMSE score <24, *i.e.* MCI 21-23, mild dementia 18-20, moderate 10-17 or severe <10) | Yes ("Feel lonely") | Yes | No |
| FI-CGA[29] | 55-item index based on data from comprehensive geriatric assessment, including similar deficits to the FI, i.e. those that are associated with health status, generally increase in prevalence with age, and do not saturate before old age. | <0.35, 0.35-0.45, 0.45-0.55, >0.55 | 55 | Yes (As part of CGA) | Yes (As part of CGA) | No | Yes |
| Fried phenotype[1] | Phenotype characterised by five physical characteristics measured with standardized criteria: weakness/grip strength, exhaustion, slow walking speed, low activity, weight loss or sarcopenia. | Not frail (0 criteria), intermediate or pre-frail (1-2), or frail (≧3) | 5 | No | No | No | No |
| Hospital Frailty Risk Score (HFRS)[21] | Score derived from ICD-10 codes that were at least twice as prevalent in a cohort of individuals with one or more frailty syndromes | Low (<5), intermediate (5-15), or high (>15) risk | 109 | Yes (ICD-10 codes for dementia and delirium) | No | Yes | Yes |
| Identification of Seniors at Risk-Hospitalized Patients (ISAR-HP)[31] | A questionnaire (yes/no) assessing use of walking aids, educational attainment, independence in performing IADLs and traveling. | Scores ≧ 2 indicate high-risk for functional decline | 4 | No | No | No | Yes |

| | | | | | | | |
|---|---|---|---|---|---|---|---|
| Maastricht Frailty Screening Tool for Hospitalised Patients (MFST-HP)[34] | A questionnaire (yes/no) assessing physical, psychological and social domains. | Not reported | 15 | Yes ("Patient is known to have a memory problem," and “disorientation and/or confusion at home or during previous admissions”) | Yes ("Patient experiences loneliness") | No | Yes |
| Method for assigning Priority Levels-Acute Care (MAPLe-AC)[32] | Algorithm that includes function, cognitive impairment, behavior issues, decline in decision-making, medication management issues, pressure/stasis ulcers, or problems with the environment. | Low, mild, moderate, high or very high-risk. | 8 | Yes (InterRAI-HC data: “decision making deterioration,” “behavioral symptoms,” CPS>1-3) | No | No** | Yes |
| Multidimensional Prognostic Index (MPI)[33] | An index ranging from 0-1, which includes 8 items (ADLs, IADLs, cognition, comorbidity, nutrition, pressure sore risk, polypharmacy, social support) which were selected from a CGA-like data set. | Low (<0.33), intermediate (0.34-0.66), or high (>0.66) risk | 8 | Yes (SPMSQ, minor 4-7, major 8-10) | Yes (live alone, with family or in institution) | No** | Yes |
| Reported Edmonton Frail Scale (REFS)[34] | Self-reported questionnaire scored from 0-18, including cognition, general health status, function, medication use, social supports, nutrition, mood, continence, and performance. | Not frail (0-5), apparently vulnerable (6-7), mild frailty (8-9), moderate frailty (10-11), or severe frailty (12-18). | 18 | Yes (errors on clock-drawing test) | Yes ("Can you count on someone who is willing and able to meet your needs?") | No | Yes |

| | | | | | | | |
|---|---|---|---|---|---|---|---|
| Study of Orthopaedic Fractures Index (SOF-I)[36] | Index consisting of weight loss of more than 5%, inability to rise from a chair 5 times without using arms, and self-reported reduced energy level. | Robust (0 criteria), intermediate (1), frail (2-3) | 3 | No | No | No | No |
| Survey of Health, Ageing and Retirement in Europe Frailty Index (SHARE-FI)[70] | A self-reported screening questionnaire based on the Fried model of frailty. Adjusted for sex. | Not frail, pre-frail, frail (calculator available for scoring) | 6 | No | No | No | No |

*****i.e. as opposed to prospectively available clinical data. **ICD codes or administrative data in some form were used in the development or use of the tool (or it was unclear if they were used), but the tool can be used with clinical data. MMSE = Mini-Mental State Examination. SPMSQ = Short Portable Mental Status Questionnaire. CPS = Clinical Performance Scale.

**Table S12. Summary of data sources used in frailty instruments**

| Data sources | CFS | Brief | | Fried | | | | | Deficit | | | | | Multi-domain | | | | Other | | | | |
|---|---|---|---|---|---|---|---|---|---|---|---|---|---|---|---|---|---|---|---|---|---|---|
| | CSHA CFS | ISAR-HP | VMS | Fried | FAM | FRAIL | SHARE-FI | SOF-1 | HFRS | eFI | eFI-AH | FI* | FI-CGA** | REFS | MFST-HP | MPI | MAPLe-AC | BISEP | FCS-1 | Frail-PPS | DF-GFS | Soong 2015 |
| Administrative codes (ICD-9/10 or other) | | | | | | | | | x | x | x | x | | | | ? | | x | | | x | x |
| Questionnaire | | x | x | | x | x | x | | | | | | | x | x | | | | | x | | |
| Physical measurements | | | | x | x | | | x | | | x | x | | | | | | | | x | | |
| Routine clinical data*** | | | x | | x | | | | | | x | x | | | | x | | x | | x | | |
| Judgement-based | x | | | | | | | | | | x | | | | | | | | | | | |
| Comprehensive geriatric assessment | | | | | | | | | | | | | x | | | | x | | | | | |
| Standardized scales (e.g. Katz Index) | | | x[a] | | | | | | | | x[b] | x[c] | | | | x[d] | | | x[e] | x[f] | | |
| Total no. items | 1 | 4 | 6 | 5 | 14 | 5 | 5 | 3 | 109 | 36 | 37 | 31+ | 55 | 18 | 15 | 8 | 8 | 5 | 2 | 31 | 11 | 7 |
| Time required to perform (average, in minutes) | | | | | | | | | | | | | 25 | 10 | | 20 | | | | | | |

*Multiple variations of the FI were included in this review

**The FI-CGA is considered to be a variation of the FI, but was presented separately here because it relied on a different data source.

*** Routinely acquired data from the patient's chart

a: Includes the MUST (Malnutrition Universal Screening Tool) and Katz Index

b: May include Brief Cognitive Screen Six Item Screener, Confusion Assessment Method—CAM, Waterlow Scale, Ontario Modified Stratify Falls Risk Screen and speech pathology review.

c: Includes the MMSE (Mini-Mental State Examination)

d: Includes the Katz index of independence in activities of daily living, Lawton instrumental activities of daily living scale, SPMSQ (Short Portable Mental Status Questionnaire), Exton-Smith scale, Mini-nutritional assessment and CIRS-CI (Cumulative rating of illness scale--Comorbidity-index)

e: Includes the Barthel index and Exton-Smith score

f: Includes the 10-item Short Portable Mental Status Questionnaire and 6-item Lubben social network scale.

## Risk of bias

**Table S13. Study-level risk of bias assessment for all admissions and general medicine admissions.**
Risk of bias was assessed with a modified version of the Joanna Briggs Institute Critical Appraisal Checklist for Prevalence Studies and Cohort Studies.[22,71]
The items assessed for prevalence included (1) “Was the sample frame appropriate to address the target population and was sufficient coverage of the sample frame achieved?” (2) “Were study participants recruited appropriately?” (3) “Was the sample size adequate?” (4) “Were the study subjects and setting described in detail?” (5) “Were valid methods used for the identification of the condition?” (6) “Was the condition measured in a standard, reliable way for all participants?” (7) “Was the response rate adequate, and if not, was the low response rate managed appropriately?”[71]
The items assessed for other outcomes included (1) “Were the two groups similar and recruited from the same population?” (2) “Were the exposures measured similarly to assign people to both exposed and unexposed groups?” (3) “Was the exposure measured in a valid and reliable way?” (4) “Were confounding factors identified and handled appropriately?” (5) For the outcome, “discharge to a location other than home” ONLY: “Were the groups/participants free of the outcome at the start of the study (or at the moment of exposure)”? (6) “Were the outcomes measured in a valid and reliable way?” (7) “Was the follow up time reported and sufficient to be long enough for outcomes to occur? If the length of follow-up differed between groups, were appropriate strategies used to address it?” (8) “Was follow up complete, and if not, were the reasons to loss to follow up described and explored?”
Reporting of the statistical analysis was also evaluated, “Was there appropriate statistical analysis for all relevant outcomes?”
Due to incomplete or unclear reporting of methods, some items were assessed as unclear and in other cases, inferences were made according to the author’s judgement.

### All admissions

| | **JBI Prevalence Checklist** | | | | | | | | **JBI Cohort Checklist** | | | | | | | | | **Statistical reporting** | **Rationale** |
|---|---|---|---|---|---|---|---|---|---|---|---|---|---|---|---|---|---|---|---|
| **Author** | **1** | **2** | **3** | **4** | **5** | **6** | **7** | **Overall** | **1** | **2** | **3** | **4** | **5** | **6** | **7** | **8** | **Overall** | **1** | |
| Asmus-Szepesi et al., 2013 | H | H | L | L | L | L | H | High | | L | L | H | | H | U | H | Moderate | H | The prevalence estimate is unlikely to be correct due to the large number of individuals who declined to participate or were deemed ineligible (i.e., because they were terminally ill, unable to follow instructions) – n=2211 (83%) or were lost to follow-up – n=181 (39%) at 3 months, and unclear recruitment strategy. The outcome data reported is likely to be correct, but outcomes were assessed by home visits. Illness severity was not controlled for and statistical reporting was poor. |
| Fujita et al., 2022 | H | L | L | L | L | L | L | Moderate | L | L | L | H | | L | L | H | Moderate | H | The prevalence estimate is likely to be correct, because most people admitted for >24 h had sufficient data to be included (although about 30% were excluded, their median LOS was <0.2 days). Outcomes are likely to be correct. Illness severity and comorbidity were not controlled for, and follow-up for outcomes was only done in hospital records. |

| | | | | | | | | | | | | | | | | | | | |
|---|---|---|---|---|---|---|---|---|---|---|---|---|---|---|---|---|---|---|---|
| Geriatric Medicine Research Collaborative 2019 | H | H | L | H | L | L | L | High | | | | | | | | | | L | The prevalence estimate is unlikely to be correct, because convenience sampling was used, sampling occurred on one day, participant characteristics were not well described, it is unclear if the method of frailty assessment was similar for all individuals (e.g., unlikely that assessors received the same training given that they were clinical staff and not trained researchers) and no statistical adjustments were made. People at the end of life were also excluded. No outcomes of interest were measured. |
| Gilbert et al., 2022 | L | L | L | L | L | L | L | Low | L | L | L | H | | L | L | H | Moderate | L | The prevalence estimate is very likely to be correct. Illness severity was not controlled for, and only inpatient mortality could be assessed. |
| Gilbert et al., 2018 | L | L | L | L | L | L | L | Low | L | L | L | H | | L | L | L | Moderate | L | The prevalence estimate is very likely to be correct and outcomes are likely to be correct. Illness severity was not controlled for. |
| Hollinghurst et al., 2021 | L | L | L | L | L | L | L | Low | L | L | L | H | | L | L | L | Moderate | L | The prevalence estimate is very likely to be correct and outcomes are likely to be correct. Illness severity was not controlled for. |
| Lim et al., 2023 | H | U | H | L | L | L | L | High | | | | | | | | | | H | The prevalence estimate is unlikely to be correct, because the included population was limited to people without cognitive impairment or dementia and the sampling method was unclear. |
| Lujic et al., 2022 | L | L | L | L | L | L | L | Low | L | L | L | H | | L | L | L | Moderate | L | The prevalence estimate is very likely to be correct, but other outcomes are only rated as likely to be correct because illness severity was not controlled for. |
| McAlister et al., 2018 | L | L | L | L | L | L | L | Low | L | L | L | H | | L | L | L | Moderate | L | The prevalence estimate is very likely to be correct and outcomes reported are likely to be correct. The authors adjusted outcomes (e.g. mortality) for age and sex, but not illness severity. |
| Romero-Ortuno et al., 2016a, Romero-Ortuno et al., 2016b | H | L | L | L | L | L | H | Moderate | L | L | L | L | L | L | L | L | Low | L | The prevalence estimate and outcomes reported are likely to be correct. Although 28% (n=2303) of participants missing frailty assessment data and these participants differed from those without missing data, the study's authors performed multiple imputation to address this concern for which results were overall similar. Characteristics of frail vs not frail patients were not well described. Only inpatient mortality was included, so deaths occurring after discharge are unknown. |
| Soong et al., 2015 | L | L | L | H | H | L | L | Moderate | | | | | | | | | | M | The prevalence estimate is likely to be correct based on the measure used by the authors. Sex and non-geriatric co-morbidities were not described. The method for frailty has not been validated. Confidence intervals were not reported, although sample size was very large. No other outcomes were measured. |

| | | | | | | | | | | | | | | | | | | | |
|---|---|---|---|---|---|---|---|---|---|---|---|---|---|---|---|---|---|---|---|
| Soong et al., 2019 | L | L | L | H | U | L | L | Moderate | L | L | L | H | | L | L | L | Moderate | H | The prevalence estimate and outcomes reported are likely to be correct. Nearly all eligible admissions were included, however, some details, e.g., deaths in the validation cohort were unclear. In addition, the accuracy of the administrative data used was not reported and the measure was not compared to other frailty tools. Descriptive statistics were not provided. Illness severity and comorbidity were not controlled for. |
| Street et al., 2021 | L | L | L | L | L | L | L | Low | L | L | L | H | | L | L | L | Moderate | L | The prevalence estimate is very likely to be correct and outcomes reported are likely to be correct. The authors did not adjust outcomes for illness severity. |
| Timmons et al., 2015 | U | U | L | L | L | L | L | Low | | | | | | | | | | L | The prevalence estimate is likely to be correct, although people moribund on admission were excluded, potentially resulting in slight underestimation of prevalence. The study provides adequate descriptions of the frailty assessment process, and participant characteristics. The frailty tool was validated in a community-based population, and confidence intervals are not reported. No other outcomes were measured. |
| Wallis et al., 2015 | L | L | L | L | L | L | H | Moderate | L | L | L | H | | L | L | L | Moderate | L | The prevalence estimate is unlikely to be correct,, because over 20% of participants were missing frailty scores and those missing scores differed in age, cognitive function, ward admitted to, LOS and mortality rates. However, outcomes are likely to be correct, although rates of comorbidity were much higher among frail participants but were adjusted for in multivariate analyses. Illness severity was not controlled for. |
| Warnier et al., 2017, Warnier et al., 2019 | H | L | L | H | L | L | L | Moderate | L | L | L | H | L | L | L | L | Moderate | L | The prevalence estimate is likely correct because most eligible participants were included (n=2691/2787; 97%), but outcomes are unlikely to be correct. However, the studies did not describe participant's comorbidities, or account for age or sex or other confounders, such as illness severity in analyses. It was not clear to what extent loss to follow-up occurred. In addition, it was unclear what counted as a 'regular hospital ward,' and people who were not community-dwelling were excluded. |

**General medicine admissions**

| | **JBI Prevalence Checklist** | | | | | | | | **JBI Cohort Checklist** | | | | | | | | | **Statistical reporting** | **Rationale** |
|---|---|---|---|---|---|---|---|---|---|---|---|---|---|---|---|---|---|---|---|
| **Author** | **1** | **2** | **3** | **4** | **5** | **6** | **7** | **Overall** | **1** | **2** | **3** | **4** | **5** | **6** | **7** | **8** | **Overall** | **1** | |

| | | | | | | | | | | | | | | | | | | | |
|---|---|---|---|---|---|---|---|---|---|---|---|---|---|---|---|---|---|---|---|
| Anani et al., 2020 | H | L | L | L | L | L | U | High | L | L | L | H | | U | U | U | Unclear | H | The prevalence estimate is unlikely to be correct, but there was insufficient information to assign a risk of bias to other outcomes. The authors excluded patients over 85 years of age and others with severe disease, because they assumed these individuals to be frail. It was not clear at what point during admissions frailty was measured, though the measure seemed likely to be reliable. It is unclear how outcomes were determined and how many participants were lost to follow-up or refused to participate. Illness severity was not controlled for. |
| Belga et al., 2016; Kahlon et al., 2015; McAlister et al., 2019 | H | U | L | L | L | L | H | High | L | L | L | H | | L | L | L | Moderate | L | The prevalence estimate is unlikely to be correct and the outcomes reported were likely to be correct. It is unclear if potentially eligible patients who refused screening had similar characteristics to included patients or not. In addition, the sample frame was limited (e.g. people who were discharged to long-term care were excluded) and sampling method was not described clearly. Results were adjusted for age and sex, but not for other factors, e.g. comorbidities that differed between groups and illness severity, although LACE score was adjusted for. |
| Bonjour et al., 2021 | L | L | L | L | L | L | L | Low | L | L | L | H | U | L | L | L | Moderate | L | The prevalence estimate is very likely to be correct and outcomes reported are likely to be correct. Illness severity was not controlled for and it was not clear if participants were free from the outcome of institutionalisation at the start of the study. |
| Buurman et al., 2012 | H | L | L | L | L | L | H | High | L | L | L | H | | L | L | L | Moderate | L | The prevalence estimate is unlikely to be correct, but outcomes are likely to be correct. Individual consent was required and a large number of eligible participants (>20%) refused participation. Non-participants differed in age and mortality than participants. Loss to follow up was unclear, but probably small for mortality (i.e. data were obtained from a municipal registry). Illness severity was not controlled for. |
| Dani et al., 2018 | L | L | L | L | L | L | L | Low | L | L | L | H | | L | L | L | Moderate | L | The outcomes are likely to be correct and prevalence very likely to be correct, but descriptive statistics were not provided for frail vs not frail participants, and mortality was only adjusted for age and sex, but not comorbidity or illness severity. |
| Eckart et al., 2019 | L | L | L | L | L | L | L | Low | L | L | L | H | L | L | L | H | Moderate | L | The prevalence estimate and outcomes reported are very likely to be correct. Variation in coding of ICD-10 scores could result in the misclassification of frailty, however, it is unclear how large this effect would be. In addition, the HFRS has been validated in similar settings previously. Follow-up was done by telephone and it was unclear how complete it was. Illness severity was measured, but not controlled for. |

| | | | | | | | | | | | | | | | | | | | |
|---|---|---|---|---|---|---|---|---|---|---|---|---|---|---|---|---|---|---|---|
| Eeles et al., 2012 | L | L | H | L | L | L | H | Moderate | L | L | L | H | | L | L | L | High | L | The prevalence estimate is likely to be correct, but outcomes reported are unlikely to be correct. Approximately 70% of eligible individuals were enrolled. Additional description of study participants could have been provided. No other major confounding factors, including age were adjusted for. |
| El-Sharkaway et al., 2015 | H | U | H | L | L | L | H | High | | | | | | | | | | L | The prevalence estimate is unlikely to be correct. A large number of individuals who were screened were excluded (n=1209/1409; 86%), including due to being severely ill and refusal to participate. The sampling method was unclear. No other outcomes relevant to the review were reported. |
| Evans et al., 2014 | U | H | L | L | L | L | H | High | L | L | L | H | H | L | L | L | Moderate | L | The prevalence estimate is unlikely to be correct and outcomes are likely to be correct. Even though probability sampling was not used, about 56% (n=950/1702) of the eligible sample was enrolled. It was unclear if sufficient coverage of sample frame was achieved, based on the information provided in the study. The modified method used by the authors has not been validated previously. Co-morbidities were reported and differed between frailty groups, but not included in multivariable models. Risk ratios for the association between age and sex and mortality adjusted for frailty were reported, but the full model including adjusted risk ratios for frailty was not reported. Illness severity was not controlled for. The authors did not distinguish between participants who were newly discharged vs returning to a care home (i.e. participants might have had the outcome at the start of the study). |
| Fitriana et al., 2021 | U | L | H | L | L | L | U | High | L | L | L | H | | H | L | U | High | M | The prevalence estimate and outcomes are unlikely to be correct due to small sample size, lack of adjustment for confounding by age, sex and comorbidity and only ascertaining readmissions at a single hospital site. Also, only those who survived to discharge were included. |
| Forti et al., 2014 | L | L | L | L | U | L | H | Moderate | L | L | U | U | | L | L | L | Moderate | L | The prevalence estimate and outcomes reported are likely to be correct, but were downgraded due to the poor response rate (n=252/685; 31%) and lack of adjustment for age, comorbidity and adjustment for comorbidity, respectively. It is not clear if the SOF-I has been validated in hospital settings, and how valid or reliable assessment was, given that participants were acutely ill and some details of measurement (e.g. timing) were not clearly described. It was unclear which confounding variables were controlled for. |
| Gregoravic et al., 2016 | L | L | H | L | L | L | L | Moderate | L | L | L | H | | L | L | L | Moderate | L | The prevalence estimate and outcomes reported are likely to be correct. The study achieved near-complete coverage of the sample frame. No inter-rater reliability was reported, but the CFS, which was used in this study, generally performs well in this regard. Illness severity was not controlled for. Three-month mortality data were |

| | | | | | | | | | | | | | | | | | | | |
|---|---|---|---|---|---|---|---|---|---|---|---|---|---|---|---|---|---|---|---|
| | | | | | | | | | | | | | | | | | | | obtained from hospital records, so deaths might have been under ascertained if people died outside of the hospital. It was not clear how many individuals were lost to follow-up. |
| Hernandez-Luis et al., 2018 | H | L | H | L | L | L | L | High | L | L | L | H | | H | L | U | High | L | The prevalence and outcomes are unlikely to be correct. People with a life expectancy <6 months and persisting delirium were excluded. Attrition and missing data were not reported. It is unclear who assessed frailty, and which point during the admission and it was unclear how mortality was assessed (i.e., using hospital records or government statistics). Although a multivariable model including frailty was described, the full model including the adjusted risk ratio for frailty was not reported. Illness severity was not controlled for. |
| Hoogerduijn et al., 2012 | H | U | L | L | L | L | H | High | L | L | L | H | | L | L | U | Moderate | L | The prevalence estimate is unlikely to be correct, and outcomes are unclear. A large number of eligible participants were excluded because the outcome of interest could not be assessed in them. People who were too ill to participate were also excluded. In addition, the description of sampling methods and coverage were insufficient. No other outcomes relevant to the review were reported. Confounding, including illness severity, was not adequately accounted for and it was unclear if follow-up was complete. |
| Inouye et al., 2003 | H | L | L | L | L | L | L | Moderate | L | L | L | H | | L | L | L | Moderate | L | The prevalence estimate and outcomes reported are likely to be correct. However, it is unclear if eligible non-participants who declined participation or were excluded for 'other' reasons were similar to included participants. People with terminal illness or severe dementia were also excluded. Some potential confounding factors associated with mortality were described, but not adjusted for with respect to the association between the BISEP score and mortality, e.g. illness severity. |
| Irina et al., 2018 | H | L | H | L | L | L | L | High | L | L | L | H | | H | L | U | High | L | Insufficient information was reported to assign a risk of bias for prevalence, but the risk of bias for other outcomes was high. It was not clear how many participants were excluded, in particular, those who were excluded because they were lost to follow-up and if these individuals differed from included participants. However, people with dementia/cognitive impairment, an elevated ALT or who were unable to complete the assessment were excluded. No confounding factors were identified or adjusted for. |

| | | | | | | | | | | | | | | | | | | | |
|---|---|---|---|---|---|---|---|---|---|---|---|---|---|---|---|---|---|---|---|
| Juma et al., 2016 | H | H | H | L | L | L | L | High | L | L | L | H | L | L | L | L | High | L | The prevalence estimates and outcomes are unlikely to be correct. The sample was small (n=75) and it was unclear how participants were recruited from the ongoing project cited. In addition, people who were deemed palliative had <1 chronic health conditions or were independently ambulating at baseline were excluded. It was unclear if sufficient coverage was achieved and what the response rate was. Outcomes were not adjusted for confounding, even though there were differences between age, sex and comorbidities between exposure groups, nor was illness severity controlled for. |
| Khandelwel et al., 2012 | H | U | H | L | L | L | L | High | L | L | L | H | | U | U | U | Unclear | H | The prevalence estimate reported is unlikely to be correct, and there was insufficient information available to assess the risk of bias for other outcomes. Characteristics of participants and eligible non-participants were not reported as well as the response rate, but people with neurologic deficits were excluded. The timing of frailty assessment with the Fried phenotype was not specified, but physical performance may be affected by illness acuity. No adjustment for confounding variables was performed and it was unclear how outcomes (e.g. mortality) were assessed, as well as the degree to which complete follow-up was achieved. |
| Laura et al., 2022 | H | L | L | L | L | U | Y | Moderate | L | L | U | H | | L | L | L | Moderate | L | The prevalence estimate is likely to be correct or an underestimate, because patients from sheltered accomodation and care homes were excluded and 112 (7%) were excluded from the analysis because they could not be followed-up. It was also unclear who measured frailty and the CFS seemed to be applied in a non-standard way (i.e. taking into account comorbidity). The outcomes are likely to be correct for the target population, but the association between frailty and readmission was not adjusted for age, sex, comorbidity and illness severity. |
| McCrow et al., 2016 | H | H | H | L | L | L | H | High | | | | | | | | | | L | The prevalence estimate is unlikely to be correct. The sample size was small, response rate low, and convenience sampling was undertaken, which is susceptible to bias. People with severe/unstable comorbidities or who were moribound were excluded. No other outcomes of interest were reported. |
| Nardi et al., 2019 | H | L | L | L | L | L | L | Moderate | L | L | L | H | | L | U | U | High | L | It is unclear whether the prevalence estimate and outcomes reported are likely to be correct. The eligibility criteria were limited to people with 2+ chronic diseases. Confounding factors were not identified or adjusted for, and outcomes were assessed by calling participants, but loss to follow-up was not reported. |

| | | | | | | | | | | | | | | | | | | | |
|---|---|---|---|---|---|---|---|---|---|---|---|---|---|---|---|---|---|---|---|
| Noro et al., 2011 | L | L | L | H | L | L | L | Moderate | L | L | L | H | | L | L | L | Moderate | L | The prevalence estimate reported is likely correct, but there was insufficient information to assign a risk of bias for other outcomes. Additional information about participants could have been provided and no response rate or information on the completeness of follow-up was reported. Illness severity was not adequately controlled for. |
| Polidoro et al., 2013 | L | L | H | L | L | L | L | Moderate | | | | | | | | | | L | It is unclear whether the prevalence estimate is likely or unlikely to be correct. The method of frailty assessment was modified slightly by the authors, and it was not clear how the assessment was done. No other outcomes relevant to the review were reported. |
| Ramdass et al., 2018 | H | U | L | L | L | L | H | High | L | L | L | H | L | L | L | L | Moderate | H | The prevalence estimate and outcomes are unlikely to be correct, and outcomes are likely to be correct. A large number of participants who were screened were not included (n=272/775) and those who died, were not community-dwelling or were admitted under observation, which may impact the effect size reported. The sampling method was unclear. Also, outcomes were not adjusted for illness severity. |
| Rizza et al., 2022 | H | H | H | L | L | U | L | High | L | L | U | H | | U | L | U | High | L | The prevalence and outcomes are unlikely to be correct, because the sample was small, people with certain diagnoses (e.g., end-stage CKD, sepsis) were excluded, and consenting, sampling method and CGA details were unclear (e.g. timing). Adjusted outcomes for frailty were not available and it was not clear how deaths were followed-up. |
| Rose et al., 2014 | L | L | H | L | L | L | L | Moderate | L | L | L | H | L | L | L | L | Low | M | The prevalence estimate is likely to be correct, but outcomes are unlikely to be correct. A number of participants were excluded due difficulty on the clock drawing test, which is part of the frailty assessment. The characteristics of these participants were not reported, so it was unclear if sufficient coverage of the sample was achieved. Important confounding factors were not described or adjusted for. |
| Sharma et al., 2022 | H | L | H | L | L | U | L | High | L | L | U | H | U | U | L | H | High | L | The prevalence and outcomes are unlikely to be correct, because the sample was small and sampling method and details of frailty assessment (e.g., training of assessor) not specified. In addition, adjusted outcomes for frailty were not available and it was not clear how follow-up was done. Presumably, people already living in nursing homes were not counted in nursing home admissions. |

| | | | | | | | | | | | | | | | | | | |
|---|---|---|---|---|---|---|---|---|---|---|---|---|---|---|---|---|---|---|
| Subramanian et al., 2020 | L | L | L | L | L | L | L | Low | L | L | L | H | H | L | L | L | Moderate | M | The prevalence estimate is very likely to be correct and outcomes reported are likely to be correct. It is not clear if the coding of the diagnostic codes used in frailty assessment was accurate. Important confounding variables were not adjusted for in the association between frailty and outcomes. |

L = Low. M = Moderate. H = High. U = Unclear.

**Table S14. GRADE summary of findings table for main study outcomes**

| Outcomes | Range of anticipated absolute effects | | Relative effect | № of participants (cohorts) | Certainty of the evidence (GRADE) | |
|---|---|---|---|---|---|---|
| | **No/mild frailty** | **Moderate to severe frailty** | | | Direction of the association | Magnitude of the association |
| **All-cause mortality** | | | | | | |
| Clinically administered* | 0 to 14 (per 100) | 0 to 43 (per 100) | **RR ranged from** 0.31 to 16.06; **pooled** 2.53 [95% CI 2.13 to 3.00] | 17,662 (13 cohorts) | ⊕⊕⊕⊕ HIGH | ⊕⊕⊕◯ MODERATE[a] |
| Retrospective coding data tools* | 4 to 13 (per 100) | 8 to 17 (per 100) | **RR ranged from** 1.08 to 3.02 | 3,469,157 (8 cohorts) | ⊕⊕⊕◯ MODERATE[a] | ⊕⊕◯◯ LOW[ab] |
| **Long length of stay** (>8-10 days)** | 12 to 21 (per 100) | 35 to 52 (per 100) | **RR ranged from** 2.14 to 3.04 | 3,445,716 (6 cohorts) | ⊕⊕⊕◯ MODERATE[a] | ⊕⊕⊕◯ MODERATE[a] |
| **Discharge to NH or SNF (incl. rehab)**** | 16 to 28 (per 100) | 33 to 56 (per 100) | **RR ranged from** 1.97 to 2.82 | 27,986 (4 cohorts) | ⊕⊕⊕◯ MODERATE[a] | ⊕⊕⊕◯ MODERATE[a] |
| **Readmission** Up to 30 days | 6 to 33 (per 100) | 7 to 45 (per 100) | **RR ranged from** 0.83 to 1.94 | 3,055,748 (12 cohorts) | ⊕◯◯◯ VERY LOW[ab] | ⊕◯◯◯ VERY LOW[ab] |

**RR:** Relative risk; **NA**: Not applicable
*Where multiple time-points were reported, we used the estimate closest to up to 30 days post-discharge.
**In those who survived to discharge.

**GRADE Working Group grades of evidence:**
**High certainty:** We are very confident that the true effect lies close to that of the estimate of the effect.
**Moderate certainty:** We are moderately confident in the effect estimate: The true effect is likely to be close to the estimate of the effect, but there is a possibility that it is substantially different.
**Low certainty:** Our confidence in the effect estimate is limited: The true effect may be substantially different from the estimate of the effect.
**Very low certainty:** We have very little confidence in the effect estimate: The true effect is likely to be substantially different from the estimate of effect

a. Risk of bias. b. Inconsistency. c. Indirectness. d. Imprecision. e. Other

**Table S15. Adjusted odds ratios and other measures for main study outcomes**

| Outcome | Measure | Study | Cut-point | Crude | Adjusted | Adjusted for |
|---|---|---|---|---|---|---|
| **Mortality, in-hospital** | OR (95% CIs) | Wallis (2015) | CFS (continuous?) | Not reported | 1.60 (1.48 to 1.74) | Age, gender, Charlson comorbidity index, dementia or current cognitive concern |
| | | Fujita (2022) | HFRS >15<br><br>(ref = <15)<br><br>eFI-AH >0.25<br><br>(ref = <0.25) | Not reported | 1.06 (1.05-1.08)<br><br><br><br>2.81 (2.49-3.17) | Age, gender |
| | | Hollinghurst (2021) | HFRS 5-15<br><br>HFRS >15<br><br>(ref = 0-5) | Not reported | 1.34 (1.29-1.38)<br><br>1.44 (1.37-1.51) | Age, sex |
| | | Soong (2019) | DF-GFS (continuous) | Not reported | 1.06 (1.05 to 1.07) | Age, gender, country |
| | HR (95% CIs) | Romero-Ortuno (2016) | CFS 5<br><br>CFS 6<br><br>CFS 7-8<br><br>(ref = 1-4)*<br><br>*Imputed data for missing CFS scores | Not reported | 1.17 (0.84 to 1.61)<br><br>1.46 (1.09 to 1.96)<br><br>1.98 (1.47 to 2.66) | Age, sex, discharged by medical specialty, Charlson comorbidity index, history of dementia, current cognitive concern, illness acuity (ED-MEWS 4 or more) |
| | | Street (2021) | HFRS 5-15<br><br>HFRS >15<br><br>(ref = 0-5) | Not reported | 2.66 (2.61-2.70)<br><br>5.47 (5.32-5.62) | Age, sex, socioeconomic status, Charlson comorbidity, number of emergency admissions in preceding year, number of operation codes, ambulatory-care sensitive condition, national tariff for Healthcare Resource Group, care home resident, travel time from residence to hospital |
| **Mortality, 30-day** | OR (95% CIs) | Kahlon (2015) | CFS 5-8<br><br>(ref = 1-4) | 2.46 (0.81 to 7.45) | 2.18 (0.65 to 7.39) | Age, sex |

| | | | | | | |
|---|---|---|---|---|---|---|
| | | Gilbert (2018) | HFRS 5-15<br><br>HFRS >15<br><br>(ref = 0-5) | 2.09<br><br>2.56 | 1.65 (1.62 to 1.68)<br><br>1.71 (1.68 to 1.75) | Age, sex, socioeconomic status, admission history, Charlson comorbidity and hospital variation |
| | | Gilbert (2022) | HFRS 5-15<br><br>HFRS >15<br><br>(ref = 0-5) | 1.79 (1.76-1.82)<br><br>2.29 (2.25-2.34) | 1.34 (1.32-1.37)<br><br>1.38 (1.35-1.42) | Age, gender, hospital bed-days, primary care access, city median income and Charlson index categories |
| | | Eckart (2019) | HFRS 5-15<br><br>HFRS >15<br><br>(ref = 0-5) | 2.53 (2.09 to 3.06)<br><br>4.4 (2.94 to 6.57) | 2.65 (2.17 to 3.25)<br><br>4.83 (3.17 to 7.37) | Age, sex, comorbidities not included in HFRS |
| | HR (95% CIs) | Bonjour (2021) | HFRS 5-15<br><br>HFRS >15<br><br>(ref = 0-5) | Not reported | 1.04 (0.95-1.14)<br><br>1.31 (1.16-1.47) | Year of discharge, gender, age group, Charlson index, ICU stay, previous hospitalizations, LOS |
| **Mortality, 3-month** | | Gregoravic (2016) | CFS categories (1-3, 4-5, 7-8 and 9) | 2.5 (1.19 to 5.3) | 2.2 (0.1 to 4.67) | Gender, Charlson co-morbidity score, usual residence |
| | HR (95% CIs) | Bonjour (2021) | HFRS 5-15<br><br>HFRS >15<br><br>(ref = 0-5) | Not reported | 1.04 (0.97-1.12)<br><br>1.22 (1.12-1.34) | Year of discharge, gender, age group, Charlson index, ICU stay, previous hospitalizations, LOS |
| | | Hollinghurst (2021) | HFRS 5-15<br><br>HFRS >15<br><br>(ref = 0-5) | Not reported | 1.29 (1.26-1.32)<br><br>1.41 (1.36-1.45) | Age, sex |
| **Mortality, 1-year** | HR (95% CIs) | Bonjour (2021) | HFRS 5-15<br><br>HFRS >15<br><br>(ref = 0-5) | Not reported | 1.05 (1.00-1.11)<br><br>1.21 (1.13-1.30) | Year of discharge, gender, age group, Charlson index, ICU stay, previous hospitalizations, LOS |
| **Mortality, time-to-event** | HR (95% CIs) | Romero-Ortuno (2017) | CFS 5<br><br>CFS 6<br><br>CFS 7-8<br><br>(ref = 1-4) | Not reported | 1.08 (1.05 to 1.11)<br><br>1.31 (0.94 to 1.81)<br><br>2.10 (1.52 to 2.92) | Age, gender, discharged from medical vs. surgical specialty, Charlson co-morbidity, history of dementia, and current cognitive concern |
| | | Dani (2018) | Increase per SD (0.96 points) on FI | 5.9 (2.1 to 16) | 3.5 (1.2 to 9.9) | Age, sex |

| | | | | | | |
|---|---|---|---|---|---|---|
| | | Noro (2011) | MAPLe-AC<br>Mild<br>Moderate<br>High<br>Very high<br>(ref = Low) | Not reported | <br>0.85 (0.38 to 1.94)<br>1.31 (0.76 to 2.25)<br>2.26 (1.29 to 3.94)<br>2.71 (1.44 to 5.09) | Age, sex, reason for hospitalization, diagnosis |
| | | Buurman (2012) | Intermediate<br>High<br>(ref = Low risk) | 1.15 (0.79 to 1.67)<br>1.81 (1.29 to 2.54) | 1.10 (0.75 to 1.62)<br>1.62 (1.11 to 2.35) | Age, sex, comorbidity |
| **Readmission, 30-day** | OR (95% CIs) | Kahlon (2015) | CFS 5-8<br>(ref = 1-4) | 1.82 (1.13 to 2.97) | 1.90 (1.11 to 3.26) | Age, sex |
| | | Fitriana (2021) | FRAIL (per-point) | Not reported | 0.834 (0.455-1.528) | Function, malignancy, nutrition, depression, cognition (AMT), previous admission |
| | | Wallis (2015) | CFS (per-point) | Not reported | 1.04 (0.99 to 1.10) | Age, gender, Charlson comorbidity index, dementia or current cognitive concern |
| | | Fujita (2022) | HFRS >15<br>(ref = <15)<br>eFI-AH >0.25<br>(ref = <0.25) | Not reported | 1.02 (1.01-1.04)<br>1l11 (1.02-1.20) | Age, gender |
| | | Eckart (2019) | HFRS 5-15<br>HFRS >15<br>(ref = 0-5) | 1.04 (0.88 to 1.24)<br>1.47 (0.95 to 2.26) | 1.04 (0.87 to 1.24)<br>1.67 (1.08 to 2.59) | Age, sex, comorbidities not included in HFRS |
| | | Gilbert (2018) | HFRS 5-15<br>HFRS >15<br>(ref = 0-5) | 1.32<br>1.69 | 1.23 (1.22 to 1.25)<br>1.48 (1.46 to 1.50) | Age, sex, socioeconomic status, admission history, Charlson comorbidity and hospital variation |
| | | Gilbert (2022) | HFRS 5-15<br>HFRS >15 | 1.24 (1.22-1.26)<br>1.48 (1.45-1.51) | 1.04 (1.02-1.05)<br>1.00 (0.98-1.02) | Age, gender, hospital bed-days, primary care access, city median income and Charlson index categories |

| | | | | | | |
|---|---|---|---|---|---|---|
| | | | (ref = 0-5) | | | |
| | | Hollinghurst (2021) | HFRS 5-15<br><br>HFRS >15<br><br>(ref = 0-5) | Not reported | 1.31 (1.25-1.37)<br><br>1.52 (1.43-1.61) | Age, sex |
| | | Soong (2019) | DF-GFS (continuous) | Not reported | 1.02 (1.00 to 1.05) | Age, gender and country |
| | Average Marginal Effect (95% CIs) | Street (2021) | HFRS 5-15<br><br>HFRS >15<br><br>(ref = 0-5) | Not reported | -0.003 (-0.005, -0.001)<br><br>-0.027 (-0.031, -0.023) | Age, sex, socioeconomic status, Charlson comorbidity, number of emergency admissions in preceding year, number of operation codes, ambulatory-care sensitive condition, national tariff for Healthcare Resource Group, care home resident, travel time from residence to hospital |
| **Readmission, 90-day** | HR (95% CIs) | Hollinghurst (2021) | HFRS 5-15<br><br>HFRS >15<br><br>(ref = 0-5) | 1.37 (1.33-1.41)<br><br>1.67 (1.63-1.75) | 1.29 (1.26-1.32)<br><br>1.41 (1.36-1.45) | Age, sex |
| **Readmission, time-to-event** | HR (95% CIs) | Anani (2020) | FRAIL scale =/>3<br><br>(ref = 0-2) | Not reported | 1.59 (1.02 to 2.48) | Age, gender, diabetes mellitus, chronic kidney disease, an active malignancy and serum ALT |
| **Long length of stay** | OR (95% CIs) | Eckart (2019) | HFRS 5-15<br><br>HFRS >15<br><br>(ref = 0-5) | 3.47 (2.99 to 4.02)<br><br>9.21 (6.51 to 13.01) | 3.66 (3.14 to 4.28)<br><br>9.75 (6.83 to 13.92) | Age, sex, comorbidities not included in HFRS |
| | | Gilbert (2018) | HFRS 5-15<br><br>HFRS >15<br><br>(ref = 0-5) | 3.36<br><br>6.03 | 3.29 (3.25 to 3.30)<br><br>6.01 (5.92 to 6.10) | Age, sex, socioeconomic status, admission history, Charlson comorbidity and hospital variation |
| | | Gilbert (2022) | HFRS 5-15<br><br>HFRS >15<br><br>(ref = 0-5) | 2.56 (2.54-2.59)<br><br>3.59 (3.55-3.64) | 2.34 (2.32-2.37)<br><br>3.27 (3.22-3.32) | Age, gender, hospital bed-days, primary care access, city median income and Charlson index categories |
| | | Soong (2019) | DF-GFS (unclear) | Not reported | 1.14 (1.13 to  1.14) | Age, gender and country |

| | | | | | | |
|---|---|---|---|---|---|---|
| | | Wallis (2015) | CFS (per-point) | Not reported | 1.19 (1.14 to 1.23) | Age, gender, Charlson co-morbidity index, dementia or current cognitive concern |
| | | Fujita (2022) | HFRS >15<br>(ref = <15)<br>eFI-AH >0.25<br>(ref = <0.25) | Not reported | 1.20 (1.18-1.21)<br>2.04 (1.91-2.17) | Age, gender |
| | | Hollinghurst (2021) | HFRS 5-15<br>HFRS >15<br>(ref = 0-5) | Not reported | 1.68 (1.63-1.72)<br>2.07 (1.99-2.14) | Age, sex |
| | | Street (2021) | HFRS 5-15<br>HFRS >15<br>(ref = 0-5) | Not reported | 2.66 (2.61-2.70)<br>5.47 (5.32-5.62) | Age, sex, socioeconomic status, Charlson comorbidity, number of emergency admissions in preceding year, number of operation codes, amulatory-care sensitive condition, national tariff for Healthcare Resource Group, care home resident, travel time from residence to hospital |
| | HR (95% CIs) | Anani (2020) | FRAIL scale =/>3<br>(ref = 0-2) | Not reported | 1.55 (1.18 to 2.04) | Age, gender, diabetes mellitus, chronic kidney disease, an active malignancy and serum ALT |
| **Discharge destination, (home)** | OR (95% CIs) | Noro (2011) | MAPLe-AC<br>Low<br>Mild<br>Moderate<br>High<br>(ref = Very high) | Not reported | <br>11.63 (3.77 to 35.88)<br>8.77 (2.84 to 27.13)<br>3.67 (3.07 to 6.51)<br>1.69 (0.94 to 3.02) | Age, sex and reason for hospitalization |
| **Care home admission, 1-year** | HR (95% CIs) | Hollinghurst (2021) | HFRS 5-15<br>HFRS >15 | Not reported | 1.05 (1.00-1.11)<br>1.21 (1.13-1.30) | Age, sex |

| | | | | | | |
|---|---|---|---|---|---|---|
| | | | (ref = 0-5) | | | |
| **Living at home at one year** | OR (95% CIs) | Noro (2011) | MAPLe-AC<br>Low<br>Mild<br>Moderate<br>High<br>(ref = Very high) | Not reported | <br>3.51 (1.74 to 7.10)<br>6.14 (2.76 to 13.69)<br>2.95 (1.66 to 5.24)<br>1.79 (0.98 to 3.26) | Age, sex and reason for hospitalization |

**Table S16. Percent difference between crude and age/sex adjusted and most adjusted estimates** (i.e. age/sex, comorbidity, others) for relative risk by outcome.

| | | **% Change, Mean/SD (range)** | | |
|---|---|---|---|---|
| **Outcome** | **Adjusted for** | **Moderate frailty** | **Severe frailty** | **Studies (n)** |
| **Mortality** | Age/sex | -2.2/2.5 (-0.4 to -4) | 3/7.1 (-8 to 2) | 2 |
| | Most adjusted (all studies) | -15.2/10.2 (1 to -25.1) | -19.5/15.9 (-3.6 to -39.8) | 5 |
| **LOS** | Age/sex | 2.3 (NA) | 2.3 (NA) | 1 |
| | Most adjusted | -3.9/4.1 (-0.9 to -8.6) | -3.5/4.7 (-0.3 to -8.9) | 3 |
| **Readmission** | Age/sex | 1.9 (NA) | 1.4 (NA) | 1 |
| | Most adjusted | -9.2/6 (-4.8 to -16.1) | -12.7/14.1 (-6.1 to -32.4) | 3 |

**Legend:** % change was calculated as (adjusted - crude odds) / crude odds x 100. NA = not applicable. Most adjusted estimates included age/sex, comorbidity and any of illness severity, socio-economic status, median city income, primary care access, admission history, hospital bed-days, hospital variation, year of discharge, ICU stay and/or LOS. Sex and gender were used interchangeably.

**Table S17. C-statistic (area-under-the-receiver-operator-curve) and 95% CIs for study outcomes**

| Study name / type of measure | Frailty tool | C-statistic (95% CI) | | | | | | |
|---|---|---|---|---|---|---|---|---|
| | | Mortality | | | Length of stay (LOS) | | Discharge destination | |
| | | Dichotomous | Ordinal (3 categories) | Ordinal (4-5 categories) | Dichotomous | Ordinal (3 categories) | Dichotomous | Ordinal (3 categories) |
| **Prospective tools** | | | | | | | | |
| **Brief** | | | | | | | | |
| Asmus-Szepesi (2013) | ISAR-HP | 0.63 (0.57-0.68) | 0.66 (0.6-0.72) | 0.66 (0.6-0.73) | NA | NA | NA | NA |
| Buurman (2012) | ISAR-HP | 0.55 (0.51-0.59) | 0.57 (0.52-0.62) | NA | NA | NA | NA | NA |
| Warnier (2017, 2019) | VMS | 0.7 (0.67-0.73) | NA | NA | NA | NA | 0.6 (0.58-0.62) | NA |
| **CFS** | | | | | | | | |
| Hernandez-Luis (2018) | CFS | 0.65 (0.57-0.73) | NA | NA | NA | NA | NA | NA |
| Gregoravic (2016) | CFS | 0.61 (0.48-0.74) | NA | NA | NA | NA | NA | NA |
| Kahlon (2015) | CFS | 0.61 (0.47-0.75) | NA | NA | NA | NA | NA | NA |
| Romero-Ortuno (2016a, 2016b) | CFS | 0.6 (0.57-0.63) | 0.62 (0.59-0.65) | 0.66 (0.63-0.69) | NA | NA | NA | NA |
| Wallis (2015) | CFS | 0.66 (0.64-0.69) | 0.69 (0.66-0.72) | 0.74 (0.71-0.76) | NA | NA | NA | NA |
| **Phenotype** | | | | | | | | |
| Hernandez-Luis (2018) | Fried | 0.67 (0.59-0.75) | NA | NA | NA | NA | NA | NA |
| Irina (2018) | FRAIL | 0.6 (0.48-0.73) | NA | NA | NA | NA | NA | NA |
| **Multidimensional** | | | | | | | | |
| Inouye et al (2003) | BISEP | 0.59 (0.64-0.69) | 0.8 (0.76-0.84) | 0.82 (0.78-0.86) | NA | NA | NA | NA |
| Ramdass (2018) | REFS | NA | NA | NA | NA | NA | 0.58 (0.54-0.62) | 0.63 (0.59-0.68) |
| Warnier (2017, 2019) | MFST-HP | 0.61 (0.56-0.65) | NA | NA | NA | NA | 0.63 (0.61-0.65) | NA |
| **Retrospective or unclear** | | | | | | | | |
| **HFRS** | | | | | | | | |
| Bonjour (2021) | HFRS | 0.51 (0.5-0.53) | NA | NA | NA | NA | 0.63 (0.62-0.63) | NA |
| Eckart (2019) | HFRS | 0.62 (0.6-0.64) | 0.62 (0.6-0.65) | NA | 0.66 (0.64-0.68) | 0.67 (0.65-0.68) | 0.62 (0.60-0.63) | 0.62 (0.6-0.64) |
| Gilbert - national cohort (2018) | HFRS | 0.59 (0.59-0.59) | 0.6 (0.6-0.6) | NA | 0.65 (0.65-0.65) | 0.68 (0.68-0.68) | NA | NA |
| Gilbert (2022) | HFRS | 0.58 (0.58-0.58) | 0.59 (0.59-0.59) | NA | 0.62 (0.62-0.62) | 0.63 (0.63-0.64) | NA | NA |
| Lujic (2022) | HFRS | 0.61 (0.61-0.61) | NA | NA | 0.65 (0.64-0.65) | NA | NA | NA |
| McAlister (2018) | HFRS | 0.53 (0.53-0.53) | 0.53 (0.53-0.53) | NA | 0.64 (0.64-0.64) | 0.64 (0.64-0.65) | NA | NA |
| Street (2021) | HFRS | 0.58 (0.57-0.58) | 0.63 (0.63-0.63) | NA | 0.68 (0.68-0.68) | 0.7 (0.7-0.7) | NA | NA |
| Subramanian (2020) | HFRS | 0.64 (0.59-0.69) | NA | NA | NA | NA | NA | NA |
| **Other deficit accumulation** | | | | | | | | |
| Dani (2018) – 3 year mortality | FI | 0.55 (0.51-0.58) | 0.55 (0.51-0.59) | NA | NA | NA | NA | NA |
| Evans (2014) | FI-CGA | 0.64 (0.6-0.69) | 0.66 (0.62-0.71) | 0.69 (0.64-0.73) | NA | NA | NA | NA |
| Rizza (2022) | MPI | 0.68 (0.58-0.79) | 0.70 (0.59-0.82) | NA | NA | NA | NA | NA |

**Supplemental results - figures**

**Risk of bias summary figures**

**Figure S1. Summary of risk of bias across cohorts for prevalence**

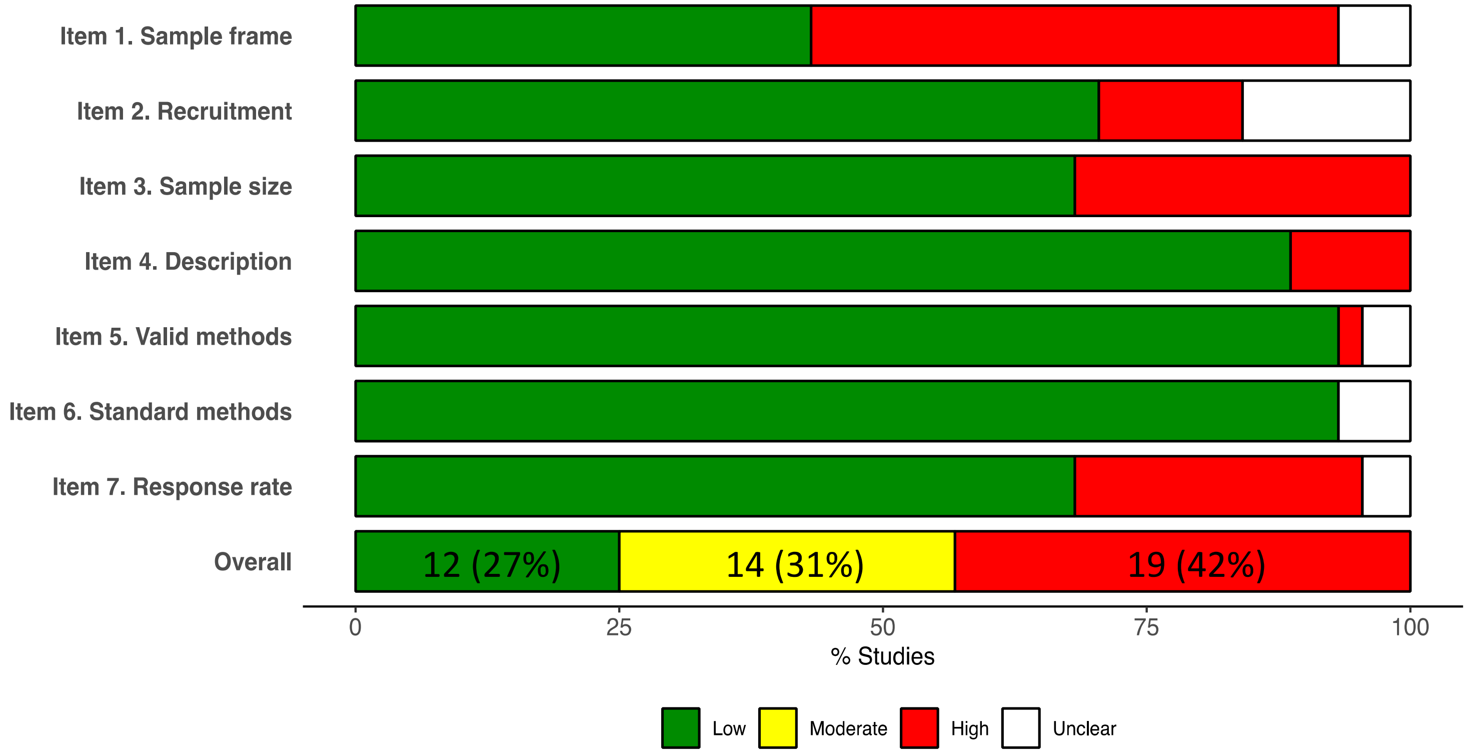


**Legend:** Risk of bias was assessed with a modified version of the Joanna Briggs Institute Critical Appraisal Checklist for Prevalence Studies. Percentages may not add up to 100% due to rounding.

**Figure S2. Summary of risk of bias across studies for cohort outcomes**

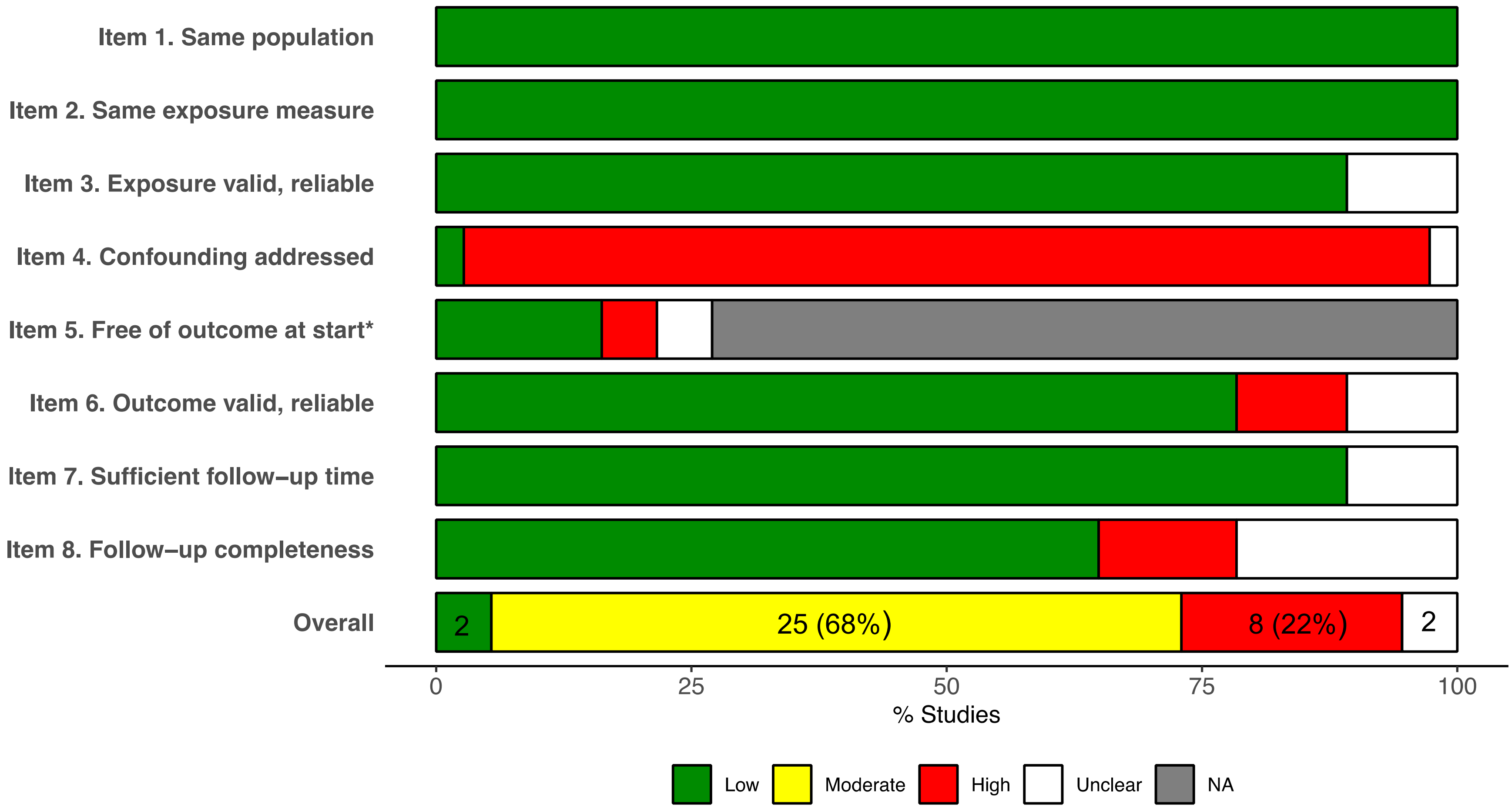


**Legend:** Risk of bias was assessed with a modified version of the Joanna Briggs Institute Critical Appraisal Checklist for Cohort Studies. Percentages may not add up to 100% due to rounding.

**Figure S3. Prevalence of moderate-to-severe frailty plotted against sample size and meta-regression equation stratified by setting.**

**(A) Studies of hospital-wide unplanned admissions.**

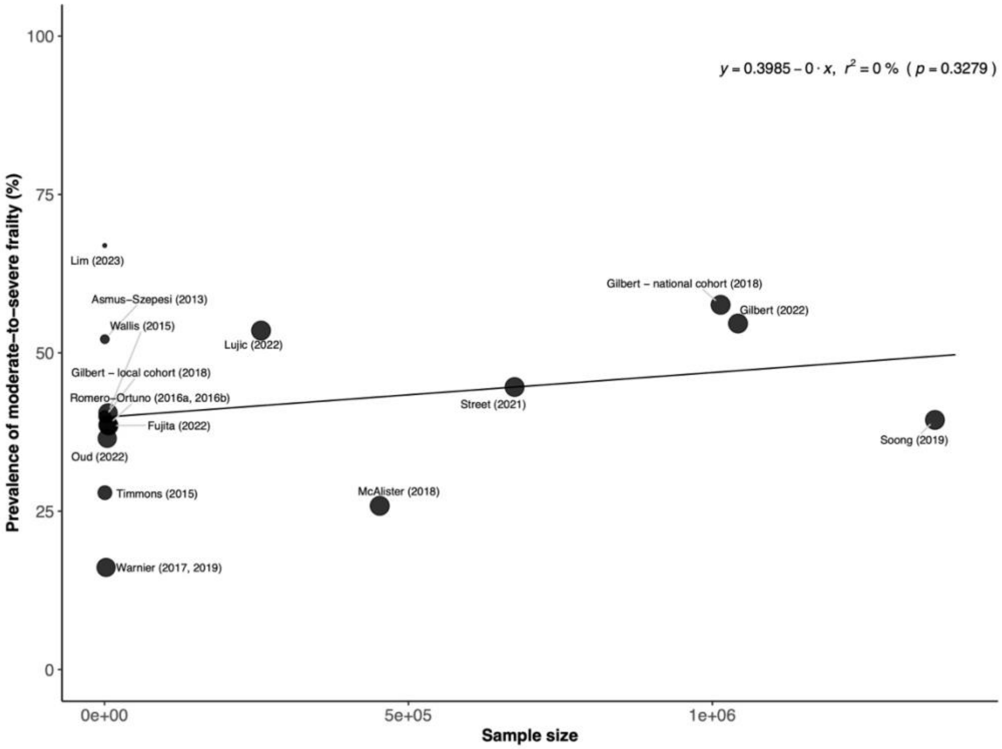


**(B) Studies of general medicine admissions.**

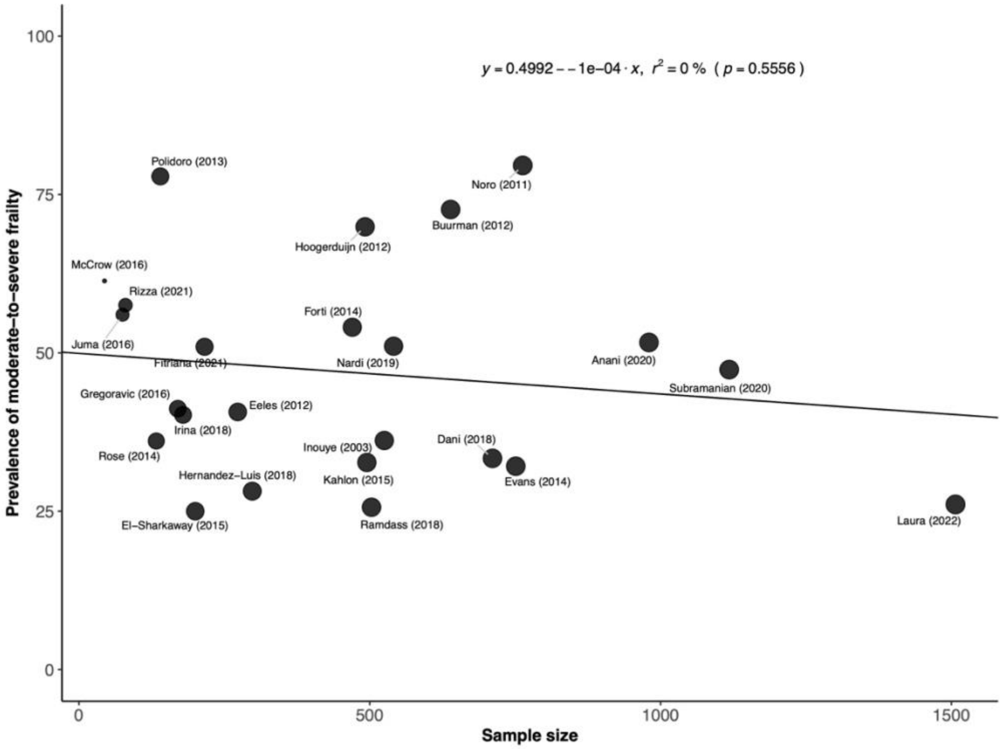


**Note:** Outliers (sample size > Q3 + 1.5*IQR) were removed from analysis, i.e. Bonjour (2021) and Eckart (2019).

**Figure S4. Funnel plots with Egger's test for the unadjusted relative risks of mortality for moderate/severe versus no/mild frailty stratified by type of measure.**

**(A) Clinically administered frailty tools**

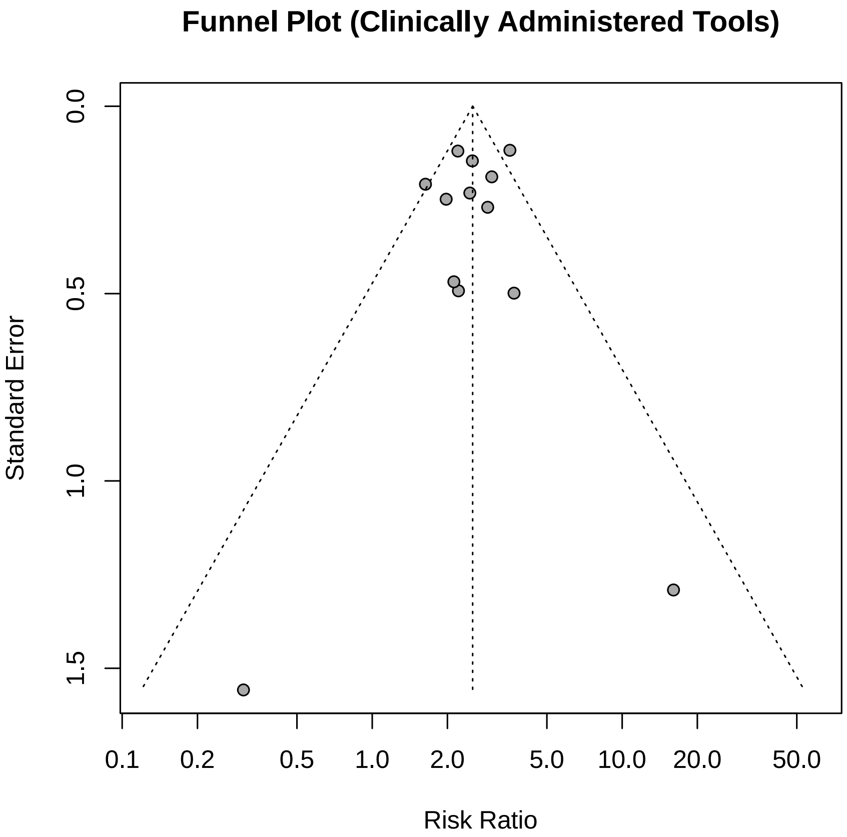


**Egger's test:** Intercept (95% CIs) = -0.33 (-1.75-1.08), p-value = 0.6527, df = 12.

**(B) Retrospective coding tools**

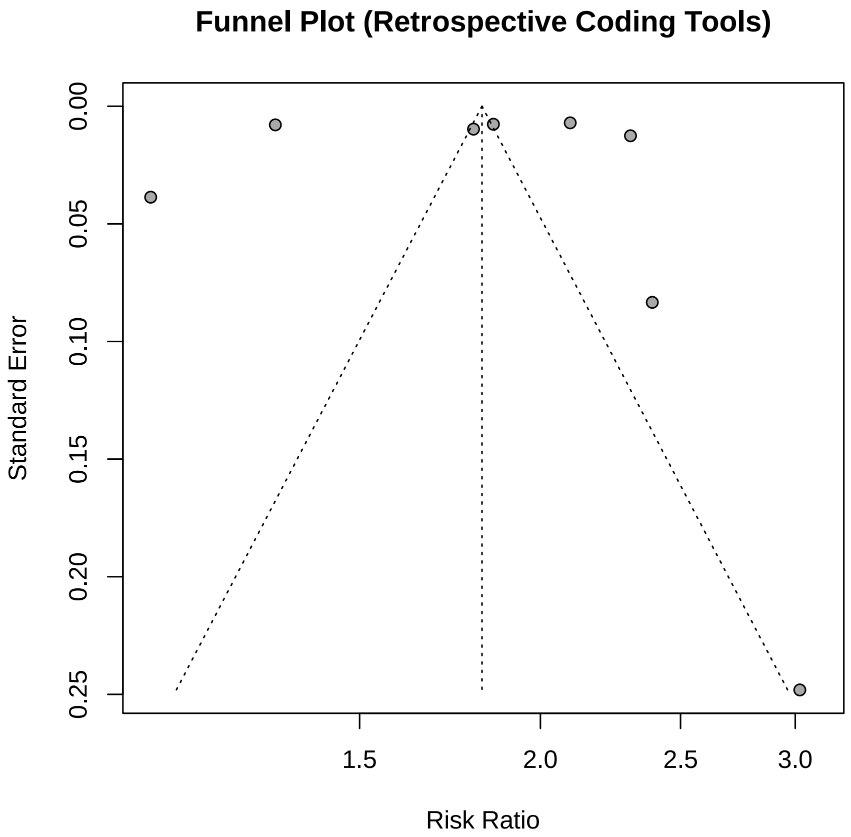


**Egger's test:** Intercept (95% CIs) = -0.35 (-26.28-25.58), p-value = 0.8666, df = 7 (Note: due to the small sample size the test is underpowered, and results cannot be relied on).

**Figure S5. Prevalence of moderate and severe frailty**

| Study or subgroup | Average age (years) | Method | Sample size | % Frail | 95% CIs |
|---|---|---|---|---|---|
| **Moderate** | | | | | |
| Timmons (2015) | 80 | SHARE−FI | 598 | 10.03 | [ 7.87; 12.70] |
| Rose (2014) | 86 | REFS | 133 | 12.03 | [ 7.54; 18.65] |
| Kahlon (2015) | 64 | 9−point CFS | 495 | 12.12 | [ 9.53; 15.29] |
| Laura (2022) | 76 | CFS | 1507 | 14.80 | [13.09; 16.68] |
| Evans (2014) | 84 | FI−CGA | 751 | 18.38 | [15.77; 21.30] |
| Inouye (2003) | 79 | BISEP | 525 | 19.62 | [16.45; 23.23] |
| Nardi (2019) | 80 | MPI | 541 | 20.33 | [17.16; 23.93] |
| Wallis (2015) | 85 | 9−point CFS | 5764 | 22.14 | [21.08; 23.23] |
| Romero−Ortuno (2016a, 2016b) | 84 | 9−point CFS | 5899 | 22.44 | [21.40; 23.53] |
| McAlister (2018) | 83 | HFRS | 452786 | 23.25 | [23.13; 23.37] |
| McCrow (2016) | 81 | 7−point CFS | 44 | 29.55 | [18.16; 44.22] |
| Gilbert − local cohort (2018) | 80 | HFRS | 569 | 30.93 | [27.27; 34.85] |
| Fujita (2022) | 84 | HFRS | 6771 | 31.86 | [30.76; 32.98] |
| Buurman (2012) | 78 | ISAR−HP | 639 | 33.02 | [29.48; 36.76] |
| Eckart (2019) | 82 | HFRS | 4957 | 33.55 | [32.25; 34.88] |
| Hollinghurst (2021) | 79 | HFRS | 126600 | 33.96 | [33.70; 34.22] |
| Rizza (2021) | 82 | MPI | 80 | 35.00 | [25.45; 45.92] |
| Lujic (2022) | 84 | HFRS | 257535 | 35.60 | [35.42; 35.78] |
| Gilbert (2022) | 85 | HFRS | 1042234 | 37.12 | [37.03; 37.21] |
| Street (2021) | 84 | HFRS | 674615 | 37.21 | [37.09; 37.32] |
| Gilbert − national cohort (2018) | 84 | HFRS | 1013590 | 37.60 | [37.51; 37.69] |
| Bonjour (2021) | 80 | HFRS | 22323 | 43.26 | [42.61; 43.91] |
| Noro (2011) | . | MAPLe−AC | 763 | 44.69 | [41.20; 48.24] |
| Juma (2016) | 81 | 9−point CFS | 75 | 50.67 | [39.60; 61.67] |
| Heterogeneity: $c^2_{23}$ = 35187.99 ($P$ < .001), $I^2$ = 100% | | | | | |
| **Severe** | | | | | |
| Kahlon (2015) | 64 | 9−point CFS | 495 | 2.22 | [ 1.25; 3.93] |
| McAlister (2018) | 83 | HFRS | 452787 | 2.63 | [ 2.58; 2.67] |
| Eckart (2019) | 82 | HFRS | 4957 | 2.90 | [ 2.47; 3.41] |
| Fujita (2022) | 84 | HFRS | 6771 | 6.66 | [ 6.09; 7.28] |
| Street (2021) | 84 | HFRS | 674615 | 7.38 | [ 7.32; 7.45] |
| Gilbert − local cohort (2018) | 80 | HFRS | 569 | 8.96 | [ 6.88; 11.59] |
| Laura (2022) | 76 | CFS | 1507 | 11.28 | [ 9.78; 12.98] |
| GMRC (2019) | 80 | 9−point CFS | 1507 | 13.34 | [11.71; 15.15] |
| Evans (2014) | 84 | FI−CGA | 751 | 13.72 | [11.44; 16.36] |
| Hollinghurst (2021) | 79 | HFRS | 126600 | 15.18 | [14.99; 15.38] |
| Romero−Ortuno (2016a, 2016b) | 84 | 9−point CFS | 5899 | 16.17 | [15.25; 17.13] |
| Inouye (2003) | 79 | BISEP | 525 | 16.57 | [13.64; 19.99] |
| Gilbert (2022) | 85 | HFRS | 1042234 | 17.51 | [17.44; 17.59] |
| Lujic (2022) | 84 | HFRS | 257535 | 17.90 | [17.75; 18.05] |
| Wallis (2015) | 85 | 9−point CFS | 5764 | 18.39 | [17.41; 19.41] |
| Gilbert − national cohort (2018) | 84 | HFRS | 1013590 | 20.00 | [19.92; 20.08] |
| Bonjour (2021) | 80 | HFRS | 22323 | 20.09 | [19.57; 20.62] |
| Juma (2016) | 81 | 9−point CFS | 75 | 21.33 | [13.58; 31.88] |
| Rizza (2021) | 82 | MPI | 80 | 22.50 | [14.73; 32.79] |
| Rose (2014) | 86 | REFS | 133 | 24.06 | [17.59; 31.99] |
| Noro (2011) | . | MAPLe−AC | 763 | 34.86 | [31.56; 38.31] |
| McCrow (2016) | 81 | 7−point CFS | 44 | 38.64 | [25.72; 53.38] |
| Buurman (2012) | 78 | ISAR−HP | 639 | 39.59 | [35.87; 43.44] |
| Heterogeneity: $c^2_{22}$ = 95484.76 ($P$ < .001), $I^2$ = 100% | | | | | |

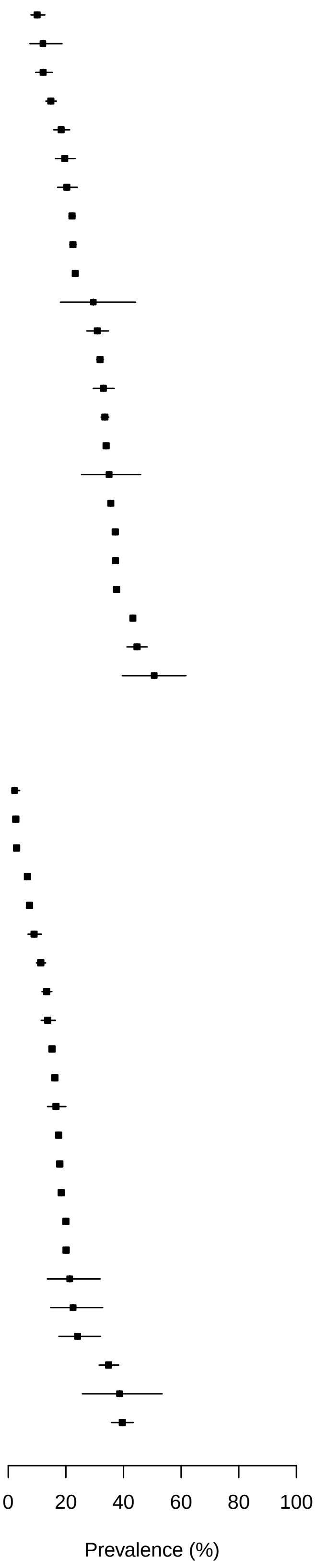


Heterogeneity: $c^2_{46}$ = 445181.17 ($P$ < .001), $I^2$ = 100%

Test for subgroup differences: $c^2_1$ = 43.75 ($P$ < .001)

**Figure S6. Prevalence of moderate/severe frailty stratified by method of ascertainment**

| Study or subgroup | Average age (years) | Method | Sample size | % Frail | 95% CIs |
|---|---|---|---|---|---|
| **Multi−domain** | | | | | |
| Warnier (2017, 2019) | 79 | MFST−HP | 2573 | 16.09 | [14.72; 17.56] |
| Ramdass (2018) | 80 | REFS | 503 | 25.65 | [22.02; 29.64] |
| Rose (2014) | 86 | REFS | 133 | 36.09 | [28.42; 44.54] |
| Nardi (2019) | 80 | MPI | 541 | 51.02 | [46.81; 55.21] |
| Rizza (2021) | 82 | MPI | 80 | 57.50 | [46.57; 67.74] |
| Noro (2011) | . | MAPLe−AC | 763 | 79.55 | [76.55; 82.26] |
| Heterogeneity: $c_5^2 = 947.31$ ($P < .001$), $I^2 = 99\%$ | | | | | |
| **Fried** | | | | | |
| Timmons (2015) | 80 | SHARE−FI | 598 | 17.89 | [15.03; 21.17] |
| Belga (2016)* | 64 | Fried | 495 | 22.02 | [18.59; 25.88] |
| Khandelwel (2012) | 66 | Fried | 250 | 33.20 | [27.66; 39.25] |
| Hernandez−Luis (2018) | 82 | Fried | 298 | 33.56 | [28.44; 39.10] |
| Irina (2018) | 72 | FRAIL | 179 | 40.22 | [33.32; 47.54] |
| Fitriana (2021) | . | FRAIL | 216 | 50.93 | [44.30; 57.52] |
| Anani (2020) | 72 | FRAIL | 980 | 51.63 | [48.50; 54.75] |
| Forti (2014) | 81 | SOF−I | 470 | 54.04 | [49.52; 58.50] |
| Heterogeneity: $c_7^2 = 284.88$ ($P < .001$), $I^2 = 98\%$ | | | | | |
| **Deficit** | | | | | |
| McAlister (2018) | 83 | HFRS | 452787 | 25.88 | [25.75; 26.00] |
| Evans (2014) | 84 | FI−CGA | 751 | 32.09 | [28.85; 35.51] |
| Dani (2018) | 83 | FI | 711 | 33.33 | [29.97; 36.88] |
| Eckart (2019) | 82 | HFRS | 4957 | 36.45 | [35.12; 37.80] |
| Fujita (2022) | 84 | HFRS | 6771 | 38.52 | [37.36; 39.68] |
| Gilbert − local cohort (2018) | 80 | HFRS | 569 | 39.89 | [35.95; 43.97] |
| Eeles (2012) | 82 | FI | 273 | 40.66 | [35.00; 46.58] |
| McAlister (2019)* | 79 | HFRS | 489 | 43.76 | [39.43; 48.19] |
| Street (2021) | 84 | HFRS | 674615 | 44.59 | [44.47; 44.71] |
| Hollinghurst (2021) | 79 | HFRS | 126600 | 46.36 | [46.09; 46.64] |
| Subramanian (2020) | 86 | HFRS | 1118 | 47.32 | [44.40; 50.25] |
| Lujic (2022) | 84 | HFRS | 257535 | 53.51 | [53.31; 53.70] |
| Gilbert (2022) | 85 | HFRS | 1042234 | 54.63 | [54.54; 54.73] |
| Gilbert − national cohort (2018) | 84 | HFRS | 1013590 | 57.60 | [57.50; 57.70] |
| Bonjour (2021) | 80 | HFRS | 22323 | 63.35 | [62.71; 63.98] |
| Polidoro (2013) | 79 | FI | 140 | 77.86 | [70.29; 83.94] |
| Heterogeneity: $c_{15}^2 = 140649.18$ ($P < .001$), $I^2 = 100\%$ | | | | | |
| **CFS** | | | | | |
| Kahlon (2015) | 64 | 9−point CFS | 495 | 14.34 | [11.53; 17.71] |
| Laura (2022) | 76 | CFS | 1507 | 26.08 | [23.92; 28.35] |
| Hernandez−Luis (2018) | 82 | 7−point CFS | 298 | 28.19 | [23.38; 33.55] |
| Romero−Ortuno (2016a, 2016b) | 84 | 9−point CFS | 5899 | 38.62 | [37.38; 39.87] |
| McCrow (2016) | 81 | 7−point CFS | 44 | 38.64 | [25.72; 53.38] |
| Wallis (2015) | 85 | 9−point CFS | 5764 | 40.53 | [39.27; 41.80] |
| Gregoravic (2016) | 77 | 9−point CFS | 170 | 41.18 | [34.05; 48.69] |
| Juma (2016) | 81 | 9−point CFS | 75 | 56.00 | [44.75; 66.67] |
| Heterogeneity: $c_7^2 = 228.94$ ($P < .001$), $I^2 = 97\%$ | | | | | |
| **Other** | | | | | |
| Inouye (2003) | 79 | BISEP | 525 | 36.19 | [32.19; 40.39] |
| Soong (2019) | . | DF−GFS | 1366187 | 39.44 | [39.35; 39.52] |
| Nardi (2019) | 80 | FCS−1 | 541 | 66.54 | [62.46; 70.39] |
| Heterogeneity: $c_2^2 = 152.45$ ($P < .001$), $I^2 = 99\%$ | | | | | |
| **Brief** | | | | | |
| Asmus−Szepesi (2013) | 76 | ISAR−HP | 460 | 52.17 | [47.61; 56.70] |
| Warnier (2017, 2019) | 79 | VMS | 2573 | 53.21 | [51.28; 55.13] |
| Lim (2023) | 74 | ISAR−HP | 366 | 66.94 | [61.97; 71.56] |
| Hoogerduijn (2012) | 78 | ISAR−HP | 492 | 69.92 | [65.72; 73.80] |
| Buurman (2012) | 78 | ISAR−HP | 639 | 72.61 | [69.03; 75.93] |
| Heterogeneity: $c_4^2 = 124.32$ ($P < .001$), $I^2 = 97\%$ | | | | | |

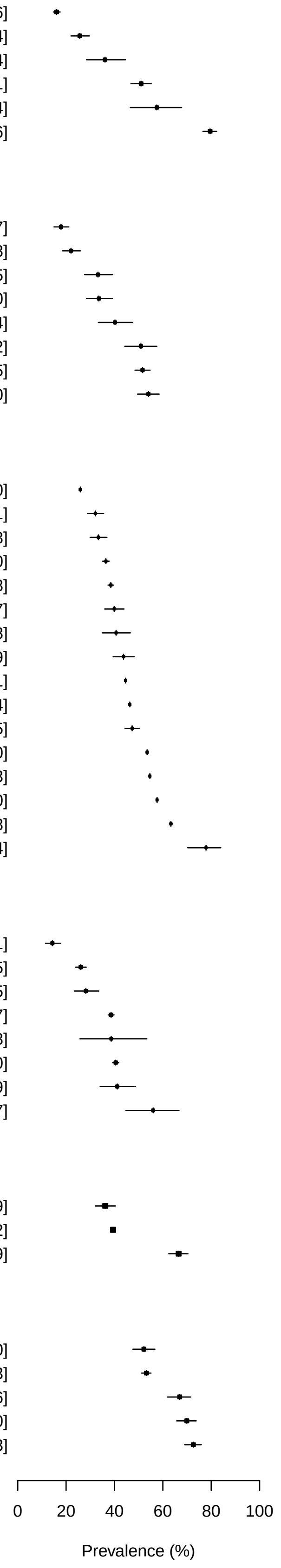


Heterogeneity: $c_{45}^2 = 187378.30$ ($P < .001$), $I^2 = 100\%$

Test for subgroup differences: $c_5^2 = 29.72$ ($P < .001$)

**Figure S7. Relative risks of mortality stratified by time-point for moderate/severe versus no/mild frailty**

| Study or subgroup | Measure | Moderate/ severe frailty Deaths | Moderate/ severe frailty Total | No/ mild frailty Deaths | No/ mild frailty Total | Weight | RR | 95% CIs |
|---|---|---|---|---|---|---|---|---|
| **In−hospital** | | | | | | | | |
| Juma (2016) | 9−point CFS | 0 | 42 | 1 | 33 | 0.6% | 0.31 | [0.01; 6.47] |
| Bonjour (2021) | HFRS | 1269 | 14141 | 655 | 8182 | 23.7% | 1.12 | [1.02; 1.23] |
| Street (2021) | HFRS | 24342 | 300816 | 16821 | 373799 | 24.5% | 1.80 | [1.76; 1.83] |
| Romero−Ortuno (2016a, 2016b) | 9−point CFS | 158 | 2278 | 114 | 3621 | 19.6% | 2.20 | [1.74; 2.79] |
| Subramanian (2020) | HFRS | 57 | 529 | 21 | 589 | 11.8% | 3.02 | [1.86; 4.92] |
| Wallis (2015) | 9−point CFS | 235 | 2336 | 97 | 3428 | 19.8% | 3.56 | [2.82; 4.48] |
| Heterogeneity: $c^2_5$ = 145.3 ($p$ < 0.001) | | | | | | | | |
| **Up to 30 days** | | | | | | | | |
| Bonjour (2021) | HFRS | 1694 | 14141 | 911 | 8182 | 15.0% | 1.08 | [1.00; 1.16] |
| McAlister (2018) | HFRS | 19502 | 117166 | 42582 | 335619 | 15.4% | 1.31 | [1.29; 1.33] |
| Gilbert (2022) | HFRS | 51769 | 569418 | 23160 | 472816 | 15.4% | 1.86 | [1.83; 1.88] |
| Gilbert − national cohort (2018) | HFRS | 71017 | 583828 | 24926 | 429762 | 15.4% | 2.10 | [2.07; 2.13] |
| Lujic (2022) | HFRS | 20986 | 137798 | 7900 | 119737 | 15.4% | 2.31 | [2.25; 2.37] |
| Eckart (2019) | HFRS | 303 | 1807 | 221 | 3150 | 13.8% | 2.39 | [2.03; 2.81] |
| Warnier (2017, 2019) | MFST−HP | 41 | 414 | 71 | 2159 | 9.5% | 3.01 | [2.08; 4.36] |
| Heterogeneity: $c^2_6$ = 2649.52 ($p$ < 0.001) | | | | | | | | |
| **Up to 90−120 days** | | | | | | | | |
| Bonjour (2021) | HFRS | 2857 | 14141 | 1474 | 8182 | 25.4% | 1.12 | [1.06; 1.19] |
| Buurman (2012) | ISAR−HP | 104 | 464 | 24 | 175 | 14.6% | 1.63 | [1.09; 2.46] |
| Gregoravic (2016) | 9−point CFS | 10 | 73 | 6 | 97 | 4.9% | 2.21 | [0.84; 5.82] |
| Evans (2014) | FI−CGA | 101 | 304 | 59 | 447 | 18.7% | 2.52 | [1.89; 3.35] |
| Warnier (2017, 2019) | MFST−HP | 82 | 414 | 164 | 2159 | 20.3% | 2.61 | [2.04; 3.33] |
| Hernandez−Luis (2018) | 7−point CFS | 24 | 84 | 21 | 213 | 11.3% | 2.90 | [1.71; 4.92] |
| Rizza (2022) | MPI | 20 | 46 | 4 | 34 | 4.8% | 3.70 | [1.39; 9.82] |
| Heterogeneity: $c^2_6$ = 89.13 ($p$ < 0.001) | | | | | | | | |
| **Up to a year** | | | | | | | | |
| Bonjour (2021) | HFRS | 5078 | 14141 | 2471 | 8182 | 33.7% | 1.19 | [1.14; 1.24] |
| Buurman (2012) | ISAR−HP | 176 | 464 | 48 | 175 | 25.6% | 1.38 | [1.06; 1.81] |
| Inouye (2003) | BISEP | 46 | 276 | 21 | 249 | 16.3% | 1.98 | [1.21; 3.22] |
| Irina (2018) | FRAIL | 10 | 72 | 7 | 107 | 7.0% | 2.12 | [0.85; 5.32] |
| Asmus−Szepesi (2013) | ISAR−HP | 59 | 240 | 22 | 220 | 17.5% | 2.46 | [1.56; 3.87] |
| Heterogeneity: $c^2_4$ = 16.34 ($p$ = 0.003) | | | | | | | | |

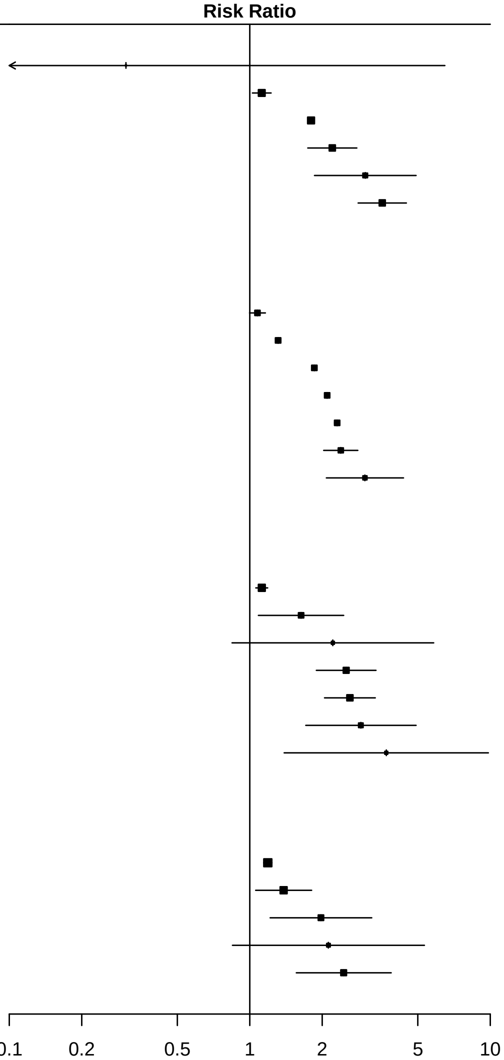


Heterogeneity: $c^2_{24}$ = 3465.95 ($p$ < 0.001)
Test for subgroup differences: $c^2_3$ = 1.75, df = 3 ($p$ = 0.62)

**Legend:** To avoid artificially reducing the standard error, when multiple estimates were reported for the same cohort (i.e. using different frailty measures), the estimate judged to have the best validity was included in the pooled estimate. Estimates not included in the pooled estimate included Warnier et al. (2017, 2019), which reported a RR for 30-day mortality of 8.97 (95% CI 4.71 to 17.10) and 90-120 day mortality of 4.53 (3.26 to 6.30) with the VMS, Hernandez-Luis et al., (2018) which reported a RR for 90-120 mortality of 3.26 (1.88 to 5.67) with the Fried phenotype.

**Figure S8. Unadjusted relative risks of mortality for moderate/severe versus no/mild frailty stratified by frailty tool type**, including studies with no events in either group

| Study or subgroup | Measure | Moderate/ severe frailty Deaths | Moderate/ severe frailty Total | No/ mild frailty Deaths | No/ mild frailty Total | Weight | RR | 95% CIs |
|---|---|---|---|---|---|---|---|---|
| **Clinically administered tools** | | | | | | | | |
| Juma (2016) | 9−point CFS | 0 | 42 | 1 | 33 | 0.4% | 0.31 | [0.01; 6.47] |
| Buurman (2012) | ISAR−HP | 104 | 464 | 24 | 175 | 10.1% | 1.63 | [1.09; 2.46] |
| Inouye (2003) | BISEP | 46 | 276 | 21 | 249 | 8.4% | 1.98 | [1.21; 3.22] |
| Irina (2018) | FRAIL | 10 | 72 | 7 | 107 | 3.5% | 2.12 | [0.85; 5.32] |
| Romero−Ortuno (2016a, 2016b) | 9−point CFS | 158 | 2278 | 114 | 3621 | 14.8% | 2.20 | [1.74; 2.79] |
| Gregoravic (2016) | 9−point CFS | 10 | 73 | 6 | 97 | 3.2% | 2.21 | [0.84; 5.82] |
| Asmus−Szepesi (2013) | ISAR−HP | 59 | 240 | 22 | 220 | 9.1% | 2.46 | [1.56; 3.87] |
| Evans (2014) | FI−CGA | 101 | 304 | 59 | 447 | 13.3% | 2.52 | [1.89; 3.35] |
| Hernandez−Luis (2018) | 7−point CFS | 24 | 84 | 21 | 213 | 7.7% | 2.90 | [1.71; 4.92] |
| Warnier (2017, 2019) | MFST−HP | 41 | 414 | 71 | 2159 | 11.0% | 3.01 | [2.08; 4.36] |
| Wallis (2015) | 9−point CFS | 235 | 2336 | 97 | 3428 | 14.9% | 3.56 | [2.82; 4.48] |
| Rizza (2022) | MPI | 20 | 46 | 4 | 34 | 3.1% | 3.70 | [1.39; 9.82] |
| Khandelwel (2012) | Fried | 5 | 83 | 0 | 167 | 0.5% | 16.06 | [1.28; 201.66] |
| **Random effects model** | | | **6712** | | **10950** | **100.0%** | **2.53** | **[2.13; 3.00]** |
| Heterogeneity: $c^2_{12}$ = 20.71 ($p$ = 0.05) | | | | | | | | |
| **Retrospective coding tools** | | | | | | | | |
| Bonjour (2021) | HFRS | 1694 | 14141 | 911 | 8182 | 13.3% | 1.08 | [1.00; 1.16] |
| McAlister (2018) | HFRS | 19502 | 117166 | 42582 | 335619 | 13.7% | 1.31 | [1.29; 1.33] |
| Street (2021) | HFRS | 24342 | 300816 | 16821 | 373799 | 13.7% | 1.80 | [1.76; 1.83] |
| Gilbert (2022) | HFRS | 51769 | 569418 | 23160 | 472816 | 13.7% | 1.86 | [1.83; 1.88] |
| Gilbert − national cohort (2018) | HFRS | 71017 | 583828 | 24926 | 429762 | 13.7% | 2.10 | [2.07; 2.13] |
| Lujic (2022) | HFRS | 20986 | 137798 | 7900 | 119737 | 13.7% | 2.31 | [2.25; 2.37] |
| Eckart (2019) | HFRS | 303 | 1807 | 221 | 3150 | 12.0% | 2.39 | [2.03; 2.81] |
| Subramanian (2020) | HFRS | 57 | 529 | 21 | 589 | 6.1% | 3.02 | [1.86; 4.92] |
| **Random effects model** | | | **1725503** | | **1743654** | **100.0%** | **1.82** | **[1.55; 2.14]** |
| Heterogeneity: $c^2_{7}$ = 2646.44 ($p$ < 0.001) | | | | | | | | |

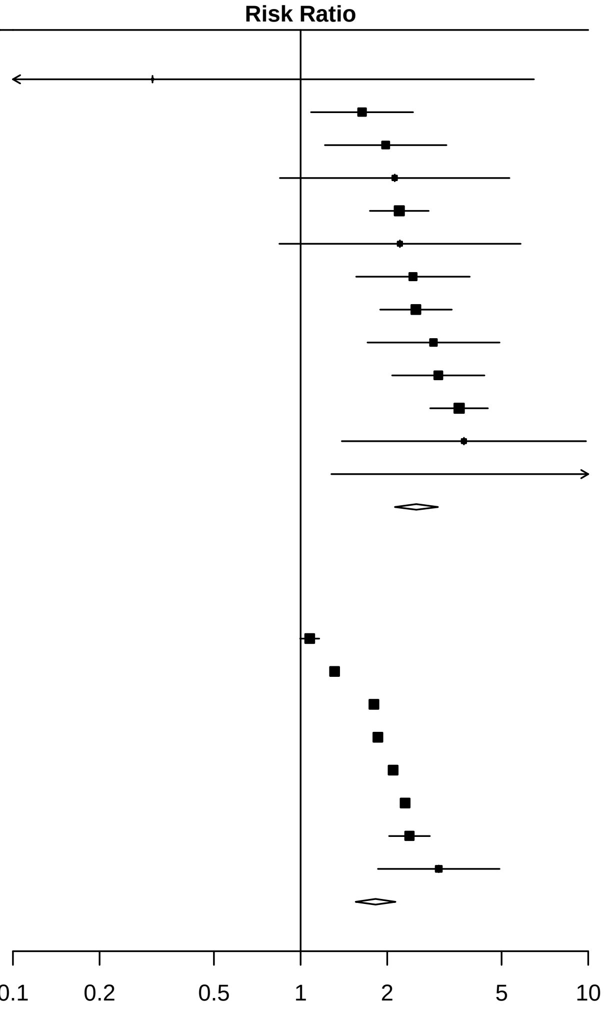


Heterogeneity: $c^2_{20}$ = 2707.64 ($p$ < 0.001)
Test for subgroup differences: $c^2_{1}$ = 7.45, df = 1 ($p$ = 0.006)

**Legend:** To avoid artificially reducing the standard error, when multiple estimates were reported for the same cohort (i.e., using different frailty measures), the estimate judged to have the best validity (e.g., validated in a similar setting previously or based on included constructs) was included in the pooled estimate. Estimates not included in the pooled estimate included Warnier et al. (2017, 2019), which reported a RR for 30-day mortality of 8.97 (95% CI 4.71 to 17.10).

**Figure S9. Unadjusted relative risks of mortality (clinically administered tools only)** for moderate and severe versus no/mild frailty (stratified)

| Study or subgroup | Measure | Moderate/ severefrailty Deaths | Moderate/ severefrailty Total | No/ mild frailty Deaths | No/ mild frailty Total | Weight | RR | 95% CIs |
|---|---|---|---|---|---|---|---|---|
| **Moderate** | | | | | | | | |
| Juma (2016) | 9−point CFS | 0 | 26 | 1 | 33 | 1.2% | 0.36 | [0.01; 9.77] |
| Buurman (2012) | ISAR−HP | 42 | 211 | 24 | 175 | 14.7% | 1.45 | [0.92; 2.30] |
| Romero−Ortuno (2016a, 2016b) | 9−point CFS | 87 | 1324 | 42 | 1021 | 16.1% | 1.60 | [1.12; 2.29] |
| Asmus−Szepesi (2013) | ISAR−HP | 20 | 115 | 22 | 220 | 13.2% | 1.74 | [0.99; 3.05] |
| Evans (2014) | FI−CGA | 45 | 166 | 59 | 447 | 16.3% | 2.05 | [1.46; 2.90] |
| Wallis (2015) | 9−point CFS | 82 | 1276 | 97 | 3428 | 17.0% | 2.27 | [1.70; 3.03] |
| Inouye et al (2003) | BISEP | 25 | 103 | 21 | 249 | 13.7% | 2.88 | [1.69; 4.90] |
| Rizza (2022) | MPI | 11 | 28 | 4 | 34 | 7.8% | 3.34 | [1.19; 9.35] |
| **Random effects model** | | | **3249** | | **5607** | **100.0%** | **1.98** | **[1.66; 2.36]** |
| Heterogeneity: $c_7^2 = 8.16$ ($p = 0.32$) | | | | | | | | |
| **Severe** | | | | | | | | |
| Juma (2016) | 9−point CFS | 0 | 16 | 1 | 33 | 0.9% | 0.40 | [0.01; 16.32] |
| Buurman (2012) | ISAR−HP | 62 | 253 | 24 | 175 | 14.5% | 1.79 | [1.16; 2.75] |
| Romero−Ortuno (2016a, 2016b) | 9−point CFS | 109 | 905 | 42 | 1021 | 15.7% | 2.93 | [2.07; 4.13] |
| Evans (2014) | FI−CGA | 56 | 138 | 59 | 447 | 16.1% | 3.07 | [2.25; 4.20] |
| Asmus−Szepesi (2013) | ISAR−HP | 39 | 125 | 22 | 220 | 13.9% | 3.12 | [1.94; 5.01] |
| Rizza (2022) | MPI | 9 | 18 | 4 | 34 | 7.5% | 4.25 | [1.52; 11.90] |
| Wallis (2015) | 9−point CFS | 153 | 1060 | 97 | 3428 | 16.8% | 5.10 | [3.99; 6.52] |
| Inouye et al (2003) | BISEP | 108 | 173 | 21 | 249 | 14.6% | 7.40 | [4.84; 11.33] |
| **Random effects model** | | | **2688** | | **5607** | **100.0%** | **3.52** | **[2.50; 4.97]** |
| Heterogeneity: $c_7^2 = 32.87$ ($p < 0.001$) | | | | | | | | |

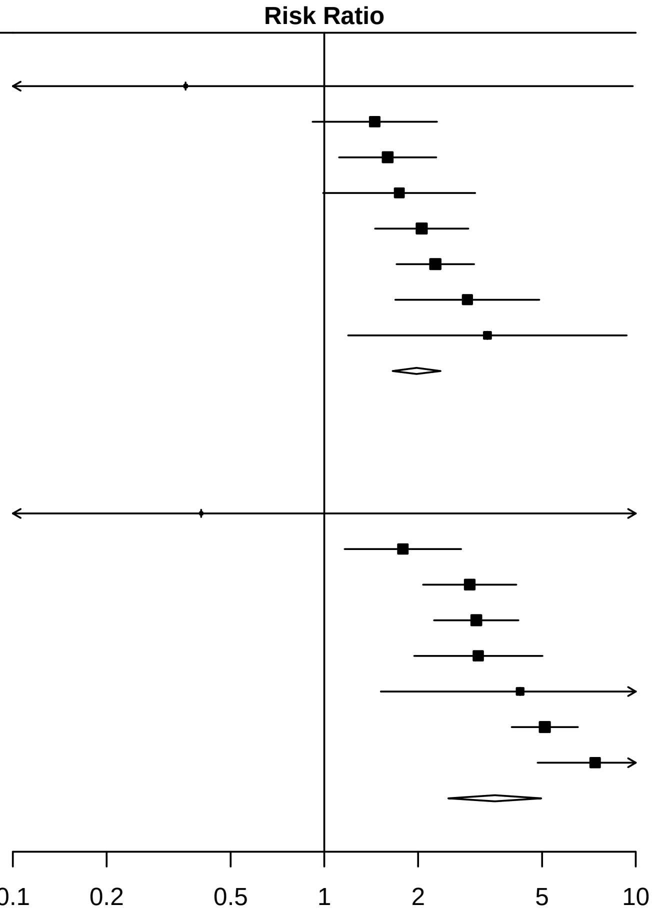


Heterogeneity: $c_{15}^2 = 76.32$ ($p < 0.001$)

Test for subgroup differences: $c_1^2 = 8.63$, df = 1 ($p = 0.003$)

**Figure S10. Bubble plot for meta-regression of moderate/severe frailty prevalence versus logged unadjusted relatives risk for all-cause mortality for moderate/severe versus no/mild frailty**

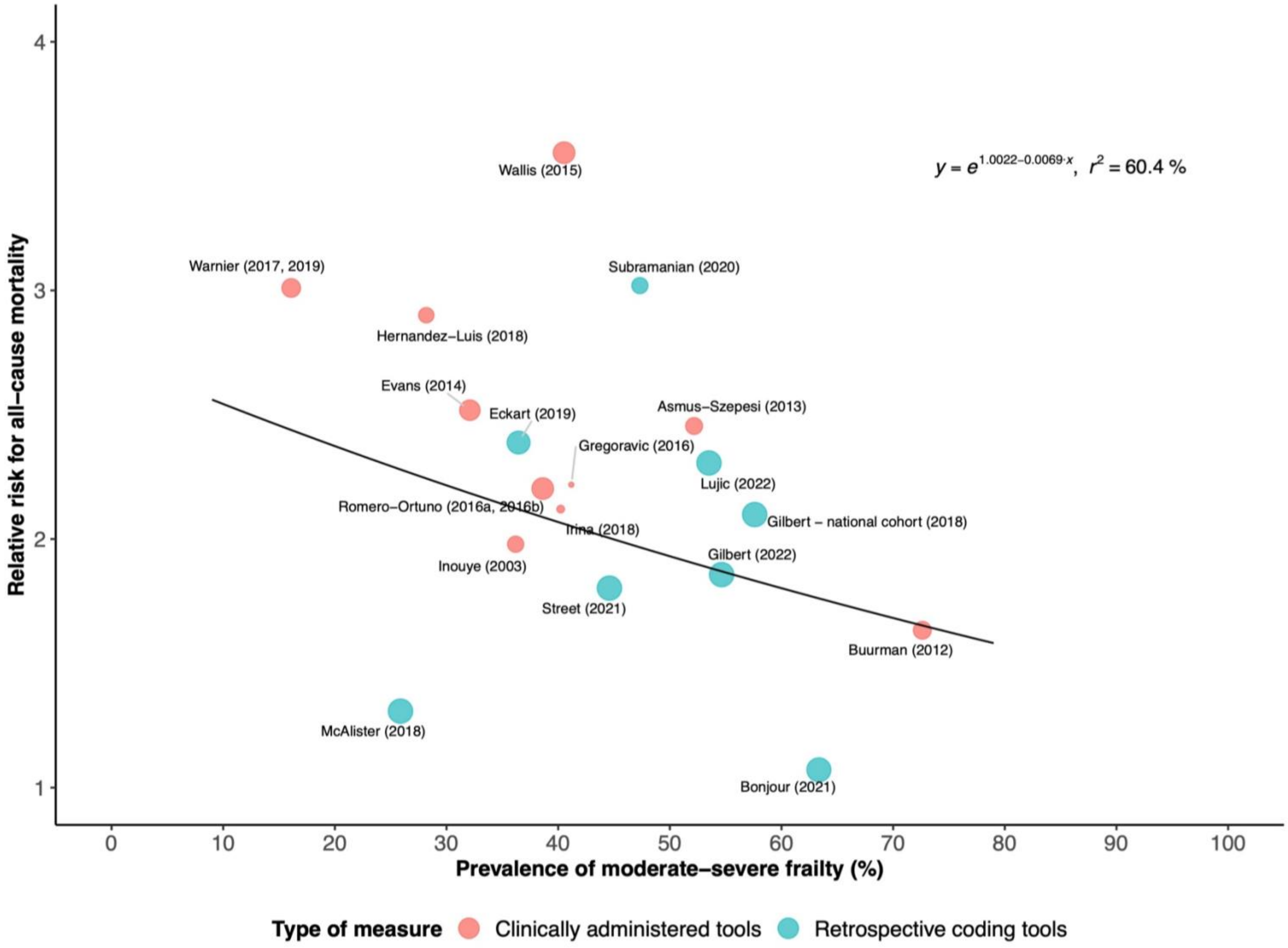


**Legend:** Point size is proportional to the random-effect weight of each study. Khandelwal et al. (2011) is not shown, because no events were reported for the robust-to-mild frailty group. To avoid artificially reducing the standard error, when multiple estimates were reported for the same cohort (i.e. using different frailty measures), the estimate judged to have the best validity (e.g. validated in a similar setting previously or based on included constructs) was included.

**Figure S11. Unadjusted relative risks for (A) length of stay >8-10 days (excluding those who died in hospital), (B) discharge to a destination other than home (nursing home or post-acute care facility) using overall denominator reported by the study authors, and (C) readmission to hospital using overall denominator reported by the study authors.**

**A. Long length of stay**

| Study | Measure | Moderate/ severe frailty Events | Moderate/ severe frailty Total | No/ mild frailty Events | No/ mild frailty Total | Weight | RR | 95% CIs |
|---|---|---|---|---|---|---|---|---|
| Forti (2014) | SOF–I | 138 | 254 | 87 | 216 | 12.7% | 1.35 | [1.11; 1.64] |
| Gilbert (2022) | HFRS | 219114 | 519202 | 85189 | 450351 | 14.7% | 2.23 | [2.22; 2.25] |
| McAlister (2018) | HFRS | 60664 | 101431 | 71430 | 307786 | 14.7% | 2.58 | [2.56; 2.60] |
| Lujic (2022) | MPI | 56729 | 117442 | 19856 | 112074 | 14.7% | 2.73 | [2.69; 2.76] |
| Eckart (2019) | HFRS | 624 | 1704 | 386 | 3056 | 14.0% | 2.90 | [2.59; 3.24] |
| Street (2021) | HFRS | 125300 | 266186 | 51958 | 356978 | 14.7% | 3.23 | [3.21; 3.26] |
| Gilbert – national cohort (2018) | HFRS | 207389 | 405019 | 50282 | 405610 | 14.7% | 4.13 | [4.09; 4.17] |

Heterogeneity: $\chi^2_6 = 13331.14$ ($p < 0.001$)

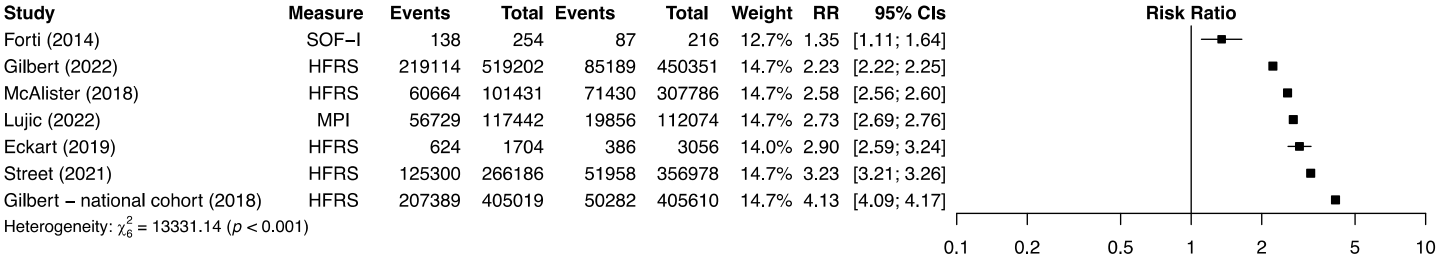


**B. Discharge to a destination other than home**

| Study | Measure | Events | Total | Events | Total | Weight | RR | 95% CIs |
|---|---|---|---|---|---|---|---|---|
| Bonjour (2021) | HFRS | 7112 | 12980 | 2111 | 7593 | 31.7% | 1.97 | [1.89; 2.05] |
| Eckart (2019) | HFRS | 588 | 1807 | 504 | 3150 | 28.8% | 2.03 | [1.83; 2.26] |
| Warnier (2017, 2019) | MFST–HP | 253 | 414 | 488 | 2159 | 28.5% | 2.70 | [2.42; 3.02] |
| Ramdass (2018) | REFS | 64 | 129 | 22 | 125 | 11.0% | 2.82 | [1.86; 4.28] |

Heterogeneity: $\chi^2_3 = 30.61$ ($p < 0.001$)

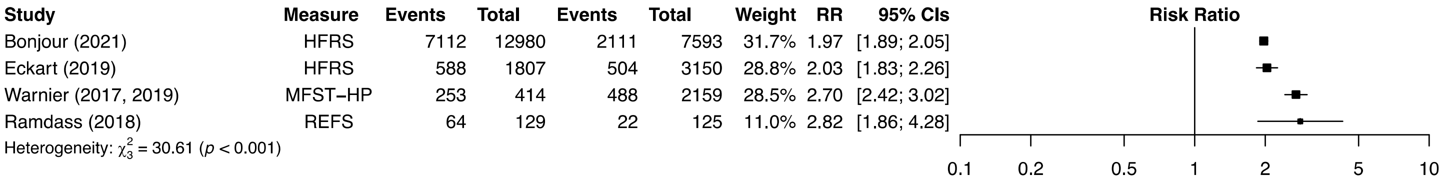


**C. Readmission to hospital**

| Study | Measure | Events | Total | Events | Total | Weight | RR | 95% CIs |
|---|---|---|---|---|---|---|---|---|
| Subramanian (2020) | HFRS | 35 | 529 | 50 | 589 | 4.8% | 0.78 | [0.51; 1.18] |
| McAlister (2018) | HFRS | 11240 | 117166 | 40320 | 335619 | 10.1% | 0.80 | [0.78; 0.81] |
| Warnier (2017, 2019) | MFST–HP | 44 | 414 | 278 | 2159 | 6.4% | 0.83 | [0.61; 1.11] |
| Eckart (2019) | HFRS | 214 | 1807 | 372 | 3150 | 8.8% | 1.00 | [0.86; 1.17] |
| Street (2021) | HFRS | 58767 | 300816 | 71769 | 373799 | 10.2% | 1.02 | [1.01; 1.03] |
| Bonjour (2021) | HFRS | 506 | 12980 | 280 | 7593 | 9.0% | 1.06 | [0.92; 1.22] |
| Wallis (2015) | 9–point CFS | 341 | 2336 | 418 | 3428 | 9.1% | 1.20 | [1.05; 1.37] |
| Gilbert (2022) | HFRS | 56635 | 569418 | 38536 | 472816 | 10.1% | 1.22 | [1.21; 1.24] |
| Gilbert – national cohort (2018) | HFRS | 110076 | 583828 | 59737 | 429762 | 10.2% | 1.36 | [1.34; 1.37] |
| Fitriana (2021) | FRAIL | 49 | 110 | 51 | 156 | 6.3% | 1.36 | [1.00; 1.85] |
| Lujic (2022) | MPI | 16847 | 137798 | 9417 | 119737 | 10.1% | 1.55 | [1.52; 1.59] |
| Anani (2020) | FRAIL | 62 | 504 | 30 | 473 | 4.8% | 1.94 | [1.28; 2.94] |

Heterogeneity: $\chi^2_{11} = 3815.27$ ($p < 0.001$)

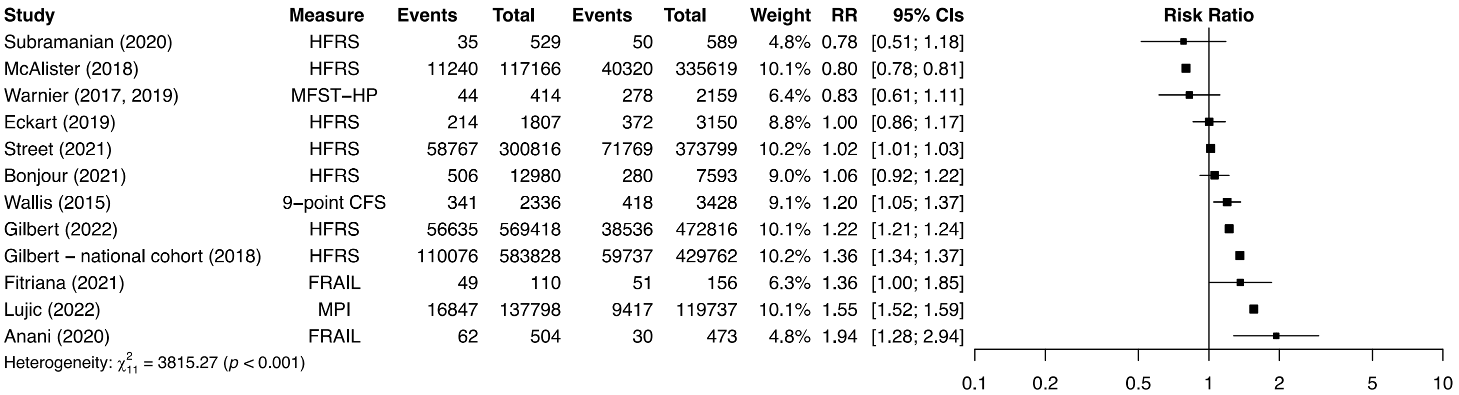


**Legend:** To avoid artificially reducing the standard error, when multiple estimates were reported for the same cohort (i.e. using different frailty measures), the estimate judged to have the best validity was retained. Warnier et al. (2017, 2019) also reported, using the VMS, a relative risk of 1.85 (95% CI 1.60 to 2.14) days for discharge to a nursing home or post-acute care facility and a relative risk for 30-day readmission of 1.09 (95% CI 0.89 to 1.34), but these estimates were not included in meta-analyses for this reason.

**Figure S12. Unadjusted relative risks of readmission for moderate/severe versus no/mild frailty in people who survived to discharge, stratified by setting**

| | | Moderate/ severe frailty | | No/ mild frailty | | | | |
|---|---|---|---|---|---|---|---|---|
| **Study** | **Measure** | **Events** | **Total** | **Events** | **Total** | **Weight** | **RR** | **95% CIs** |
| **General medicine admissions** | | | | | | | | |
| Subramanian (2020) | HFRS | 35 | 472 | 50 | 586 | 17.0% | 0.87 | [0.57; 1.32] |
| Eckart (2019) | HFRS | 214 | 1704 | 372 | 3056 | 23.4% | 1.03 | [0.88; 1.21] |
| Fitriana (2021) | FRAIL | 49 | 110 | 51 | 156 | 19.8% | 1.36 | [1.00; 1.85] |
| Laura (2022) | CFS | 115 | 278 | 216 | 898 | 22.9% | 1.72 | [1.43; 2.06] |
| Anani (2020) | FRAIL | 62 | 504 | 30 | 473 | 16.9% | 1.94 | [1.28; 2.94] |
| **Random effects model** | | | **3068** | | **5169** | **100.0%** | **1.32** | **[0.99; 1.77]** |
| Heterogeneity: $c_4^2 = 24.61$ ($p < 0.001$) | | | | | | | | |
| **All unplanned admissions** | | | | | | | | |
| Warnier (2017, 2019) | MFST−HP | 44 | 414 | 278 | 2159 | 11.9% | 0.83 | [0.61; 1.11] |
| McAlister (2018) | HFRS | 11240 | 101431 | 40320 | 307786 | 14.8% | 0.85 | [0.83; 0.86] |
| Street (2021) | HFRS | 58767 | 266186 | 71769 | 356978 | 14.8% | 1.10 | [1.09; 1.11] |
| Gilbert (2022) | HFRS | 56635 | 519202 | 38536 | 450351 | 14.8% | 1.27 | [1.26; 1.29] |
| Wallis (2015) | 9−point CFS | 341 | 2101 | 418 | 3331 | 14.1% | 1.29 | [1.13; 1.48] |
| Lujic (2022) | MPI | 16847 | 117442 | 9417 | 112074 | 14.8% | 1.71 | [1.67; 1.75] |
| Gilbert − national cohort (2018) | HFRS | 110076 | 405019 | 59737 | 405610 | 14.8% | 1.85 | [1.83; 1.86] |
| **Random effects model** | | | **1411795** | | **1638289** | **100.0%** | **1.23** | **[0.98; 1.55]** |
| Heterogeneity: $c_6^2 = 9032.83$ ($p < 0.001$) | | | | | | | | |

Heterogeneity: $c_{11}^2 = 9058.61$ ($p < 0.001$)

Test for subgroup differences: $c_1^2 = 0.15$, df = 1 ($p = 0.70$)

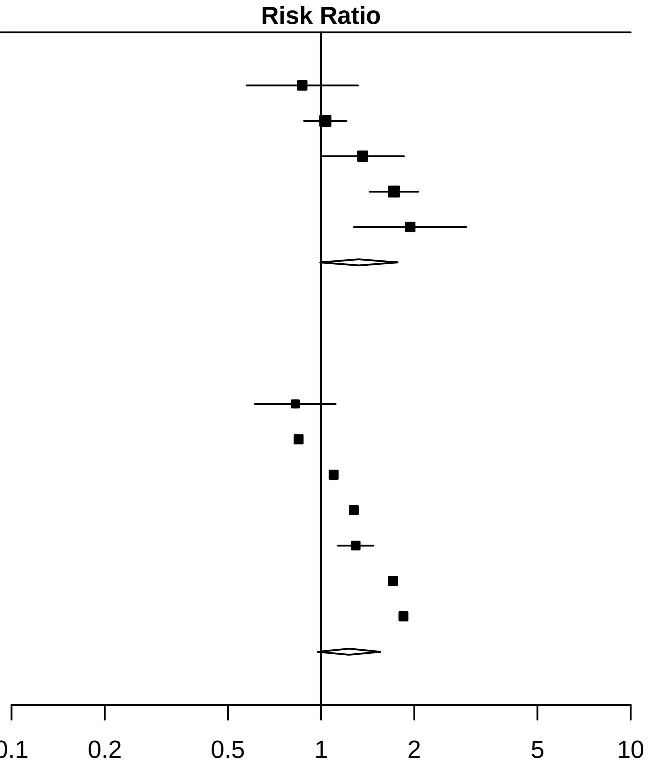

**Figure S13. Adjusted hazard (A) and odds (B) ratios for mortality by degree of frailty**

**(A) Adjusted hazard ratios for mortality**

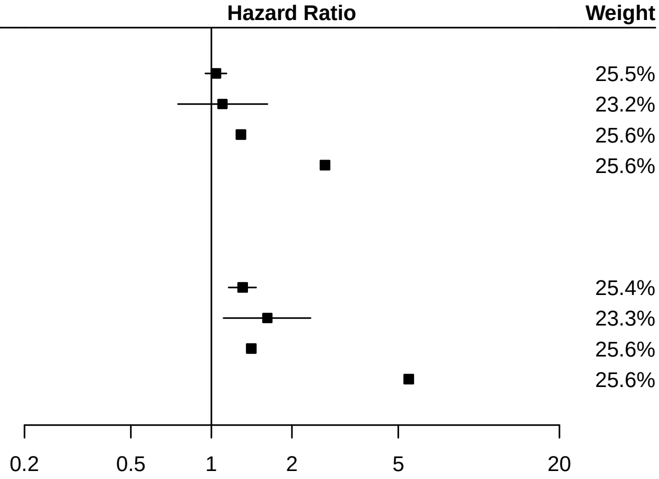


| Study | Frailty measure | HR | 95% CI | Weight |
|---|---|---|---|---|
| **Moderate** | | | | |
| Bonjour (2021) | HFRS | 1.04 | [0.95; 1.14] | 25.5% |
| Buurman (2012) | ISAR−HP | 1.10 | [0.75; 1.62] | 23.2% |
| Hollinghurst (2021) | HFRS | 1.29 | [1.26; 1.32] | 25.6% |
| Street (2021) | HFRS | 2.66 | [2.61; 2.70] | 25.6% |
| Heterogeneity: $c_3^2 = 2652.59$ ($p$ = 0) | | | | |
| **Severe** | | | | |
| Bonjour (2021) | HFRS | 1.31 | [1.16; 1.47] | 25.4% |
| Buurman (2012) | ISAR−HP | 1.62 | [1.11; 2.35] | 23.3% |
| Hollinghurst (2021) | HFRS | 1.41 | [1.36; 1.45] | 25.6% |
| Street (2021) | HFRS | 5.47 | [5.32; 5.62] | 25.6% |
| Heterogeneity: $c_3^2 = 4173.91$ ($p$ = 0) | | | | |

**(B) Adjusted odd ratios for mortality**

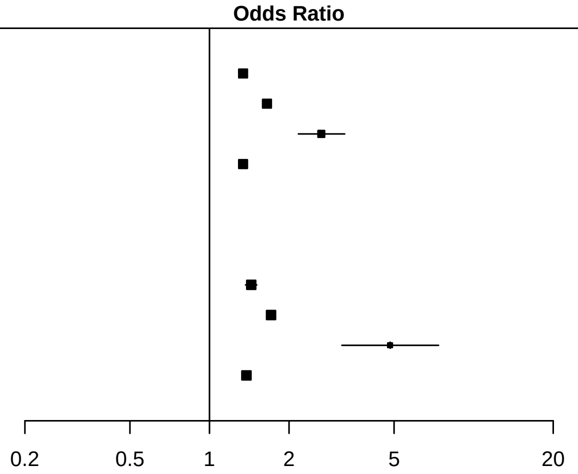


| Study | Frailty measure | OR | 95% CI | Weight |
|---|---|---|---|---|
| **Moderate** | | | | |
| Hollinghurst (2021) | HFRS | 1.34 | [1.29; 1.38] | 27.5% |
| Gilbert (2018) | HFRS | 1.65 | [1.62; 1.68] | 27.8% |
| Eckart (2019) | HFRS | 2.65 | [2.17; 3.25] | 16.8% |
| Gilbert (2022) | HFRS | 1.34 | [1.32; 1.37] | 27.8% |
| Heterogeneity: $c_3^2 = 311.88$ ($p < 0.01$) | | | | |
| **Severe** | | | | |
| Hollinghurst (2021) | HFRS | 1.44 | [1.37; 1.51] | 30.1% |
| Gilbert (2018) | HFRS | 1.71 | [1.68; 1.75] | 31.0% |
| Eckart (2019) | HFRS | 4.83 | [3.17; 7.37] | 8.0% |
| Gilbert (2022) | HFRS | 1.38 | [1.35; 1.42] | 30.9% |
| Heterogeneity: $c_3^2 = 206.07$ ($p < 0.01$) | | | | |

**Legend:** Hollinghurst et al. (2021) adjusted odds and hazard ratios for age and sex. Gilbert et al. (2018) adjusted odds ratios for age, sex, socioeconomic status, admission history, Charlson comorbidity and hospital variation. Eckart et al. (2019) adjusted for age, sex and comorbidities not included in the HFRS. Bonjour et al. (2021) adjusted hazard ratios for age group, gender, year of discharge, Charlson index, ICU stay, previous hospitalisations and length of stay. Buurman et al. (2012) adjusted hazard ratios for age, sex and comorbidity. Street et al. (2021) adjusted hazard ratios for age, sex, socioeconomic status, Charlson comorbidity, number of emergency admissions in preceding year, number of operation codes, ambulatory care sensitive condition, national tariff for Healthcare Resource Group, care home resident, travel time from residence to hospital.

**Figure S14. Adjusted odds ratios for (A) long length of stay and (B) 30-day readmission**.

**(A) Adjusted odds ratios for length of stay >8-10 days**

| Study | Frailty measure | OR | 95% CI | Weight |
|---|---|---|---|---|
| **Moderate** | | | | |
| Hollinghurst (2021) | HFRS | 1.68 | [1.63; 1.72] | 20.2% |
| Street (2021) | HFRS | 2.66 | [2.61; 2.70] | 20.2% |
| Gilbert (2018) | HFRS | 3.29 | [3.25; 3.30] | 20.2% |
| Eckart (2019) | HFRS | 3.66 | [3.13; 4.28] | 19.2% |
| Gilbert (2022) | HFRS | 2.34 | [2.32; 2.37] | 20.2% |
| Heterogeneity: $c_4^2 = 4225.32$ ($p < 0.001$) | | | | |
| **Severe** | | | | |
| Hollinghurst (2021) | HFRS | 2.07 | [1.99; 2.14] | 20.8% |
| Street (2021) | HFRS | 5.47 | [5.32; 5.62] | 20.9% |
| Gilbert (2018) | HFRS | 6.01 | [5.92; 6.10] | 20.9% |
| Eckart (2019) | HFRS | 9.75 | [6.83; 13.92] | 16.5% |
| Gilbert (2022) | HFRS | 3.27 | [3.22; 3.32] | 20.9% |
| Heterogeneity: $c_4^2 = 5019.66$ ($p < 0.001$) | | | | |

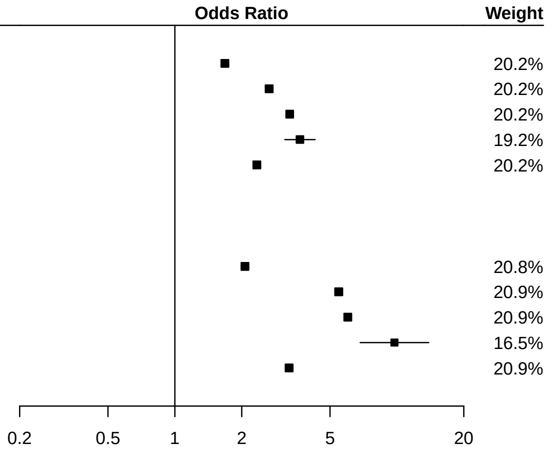


**(B) Adjusted odds ratios for 30-day readmission**

| Study | Frailty measure | OR | 95% CI | Weight |
|---|---|---|---|---|
| **Moderate** | | | | |
| Hollinghurst (2021) | HFRS | 1.31 | [1.25; 1.37] | 26.1% |
| Gilbert (2018) | HFRS | 1.23 | [1.22; 1.25] | 26.6% |
| Eckart (2019) | HFRS | 1.04 | [0.87; 1.24] | 20.8% |
| Gilbert (2022) | HFRS | 1.04 | [1.02; 1.05] | 26.6% |
| **Random effects model** | | | **[1.03; 1.31]** | **100.0%** |
| Heterogeneity: $c_3^2 = 334.47$ ($p < 0.001$) | | | | |
| **Severe** | | | | |
| Hollinghurst (2021) | HFRS | 1.52 | [1.43; 1.61] | 29.1% |
| Gilbert (2018) | HFRS | 1.48 | [1.46; 1.50] | 30.0% |
| Eckart (2019) | HFRS | 1.67 | [1.08; 2.59] | 11.1% |
| Gilbert (2022) | HFRS | 1.00 | [0.98; 1.02] | 29.9% |
| **Random effects model** | | | **[1.04; 1.79]** | **100.0%** |
| Heterogeneity: $c_3^2 = 1037.92$ ($p < 0.001$) | | | | |

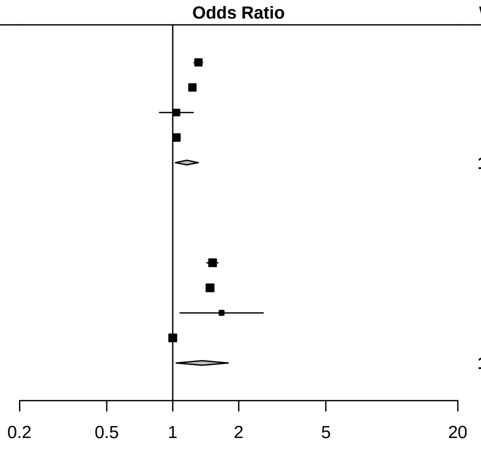


**Legend:** Hollinghurst et al. (2021) adjusted odds ratios for age and sex. Gilbert et al. (2018) adjusted odds ratios for age, sex, socioeconomic status, admission history, Charlson comorbidity and hospital variation. Eckart et al. (2019) adjusted for age, sex and comorbidities not included in the HFRS. Street et al. (2021) adjusted hazard ratios for age, sex, socioeconomic status, Charlson comorbidity, number of emergency admissions in preceding year, number of operation codes, ambulatory care sensitive condition, national tariff for Healthcare Resource Group, care home resident, travel time from residence to hospita

## PRISMA Checklist

| Section/topic | # | Checklist item | Reported on page # |
|---|---|---|---|
| **TITLE** | | | |
| Title | 1 | Identify the report as a systematic review, meta-analysis, or both. | 1 |
| **ABSTRACT** | | | |
| Structured summary | 2 | Provide a structured summary including, as applicable: background; objectives; data sources; study eligibility criteria, participants, and interventions; study appraisal and synthesis methods; results; limitations; conclusions and implications of key findings; systematic review registration number. | 2 |
| **INTRODUCTION** | | | |
| Rationale | 3 | Describe the rationale for the review in the context of what is already known. | 4-5 |
| Objectives | 4 | Provide an explicit statement of questions being addressed with reference to participants, interventions, comparisons, outcomes, and study design (PICOS). | 5 |
| **METHODS** | | | |
| Protocol and registration | 5 | Indicate if a review protocol exists, if and where it can be accessed (e.g., Web address), and, if available, provide registration information including registration number. | 6 |
| Eligibility criteria | 6 | Specify study characteristics (e.g., PICOS, length of follow-up) and report characteristics (e.g., years considered, language, publication status) used as criteria for eligibility, giving rationale. | 6 |
| Information sources | 7 | Describe all information sources (e.g., databases with dates of coverage, contact with study authors to identify additional studies) in the search and date last searched. | 6 |
| Search | 8 | Present full electronic search strategy for at least one database, including any limits used, such that it could be repeated. | 6, Suppl. |
| Study selection | 9 | State the process for selecting studies (i.e., screening, eligibility, included in systematic review, and, if applicable, included in the meta-analysis). | 6 |
| Data collection process | 10 | Describe method of data extraction from reports (e.g., piloted forms, independently, in duplicate) and any processes for obtaining and confirming data from investigators. | 6 |
| Data items | 11 | List and define all variables for which data were sought (e.g., PICOS, funding sources) and any assumptions and simplifications made. | 6 |
| Risk of bias in individual studies | 12 | Describe methods used for assessing risk of bias of individual studies (including specification of whether this was done at the study or outcome level), and how this information is to be used in any data synthesis. | 6 |
| Summary measures | 13 | State the principal summary measures (e.g., risk ratio, difference in means). | 7 |
| Synthesis of results | 14 | Describe the methods of handling data and combining results of studies, if done, including measures of consistency (e.g., $I^2$) for each meta-analysis. | 7 |
| Risk of bias across studies | 15 | Specify any assessment of risk of bias that may affect the cumulative evidence (e.g., publication bias, selective reporting within studies). | NA |
| Additional analyses | 16 | Describe methods of additional analyses (e.g., sensitivity or subgroup analyses, meta-regression), if done, indicating which were pre-specified. | 7 |
| **RESULTS** | | | |
| Study selection | 17 | Give numbers of studies screened, assessed for eligibility, and included in the review, with reasons for exclusions at each stage, ideally with a flow diagram. | 8, Fig 1 |

| | | | |
|---|---|---|---|
| Study characteristics | 18 | For each study, present characteristics for which data were extracted (e.g., study size, PICOS, follow-up period) and provide the citations. | 8, Suppl |
| Risk of bias within studies | 19 | Present data on risk of bias of each study and, if available, any outcome level assessment (see item 12). | 9, Suppl |
| Results of individual studies | 20 | For all outcomes considered (benefits or harms), present, for each study: (a) simple summary data for each intervention group (b) effect estimates and confidence intervals, ideally with a forest plot. | 10-11, Fig 2-4 |
| Synthesis of results | 21 | Present results of each meta-analysis done, including confidence intervals and measures of consistency. | 10-11 |
| Risk of bias across studies | 22 | Present results of any assessment of risk of bias across studies (see Item 15). | NA |
| Additional analysis | 23 | Give results of additional analyses, if done (e.g., sensitivity or subgroup analyses, meta-regression [see Item 16]). | 10 |
| **DISCUSSION** | | | |
| Summary of evidence | 24 | Summarize the main findings including the strength of evidence for each main outcome; consider their relevance to key groups (e.g., healthcare providers, users, and policy makers). | 12-15 |
| Limitations | 25 | Discuss limitations at study and outcome level (e.g., risk of bias), and at review-level (e.g., incomplete retrieval of identified research, reporting bias). | 14 |
| Conclusions | 26 | Provide a general interpretation of the results in the context of other evidence, and implications for future research. | 14-15 |
| **FUNDING** | | | |
| Funding | 27 | Describe sources of funding for the systematic review and other support (e.g., supply of data); role of funders for the systematic review. | 7 |

*From:* Moher D, Liberati A, Tetzlaff J, Altman DG, The PRISMA Group (2009). Preferred Reporting Items for Systematic Reviews and Meta-Analyses: The PRISMA Statement. PLoS Med 6(7): e1000097. doi:10.1371/journal.pmed1000097

## Supplemental references


1. Fried LP, Tangen CM, Walston J, et al. Frailty in older adults: Evidence for a phenotype. *J Gerontol A Biol Sci Med Sci* 2001; **56**(3): M146-M56.
2. Searle SD, Mitnitski A, Gahbauer EA, Gill TM, Rockwood K. A standard procedure for creating a frailty index. *BMC Geriatr* 2008; **8**: 24.
3. Rolfson DB, Majumdar SR, Tsuyuki RT, Tahir A, Rockwood K. Validity and reliability of the Edmonton Frail Scale. *Age Ageing* 2006; **35**(5): 526-9.
4. de Gelder J, Haenen E, Lucke JA, et al. Optimising the ISAR-HP to screen efficiently for functional decline in older patients. *Neth J Med* 2017; **75**(9): 379-85.
5. Rockwood K, Song X, MacKnight C, et al. A global clinical measure of fitness and frailty in elderly people. *CMAJ* 2005; **173**(5): 489-95.
6. Jones DM, Song X, Rockwood K. Operationalizing a frailty index from a standardized comprehensive geriatric assessment. *J Am Geriatr Soc* 2004; **52**(11): 1929-33.
7. Forti P, Maioli F, Zagni E, et al. The physical phenotype of frailty for risk stratification of older medical inpatients. *J Nutr Health Aging* 2014; **18**(10): 912-8.
8. Bonjour T, Waeber G, Marques-Vidal P. Trends in prevalence and outcomes of frailty in a Swiss university hospital: a retrospective observational study. *Age Ageing* 2021; **50**(4): 1306-13.
9. Eckart A, Hauser SI, Haubitz S, et al. Validation of the hospital frailty risk score in a tertiary care hospital in Switzerland: results of a prospective, observational study. *BMJ Open* 2019; **9**(1): e026923.
10. Warnier RMJ, van Rossum E, van Kuijk SMJ, Mulder WJ, Schols J, Kempen G. The Maastricht Frailty Screening Tool for Hospitalised Patients (MFST-HP) to Identify Non-Frail Patients. *Int J Clin Pract* 2017; **71**(9).
11. Ramdass S, Starr R, Liu X, Brennan MJ, Stefan M. Relation of frailty to discharge disposition for hospitalized community dwelling geriatric patients. *J Am Geriatr Soc* 2017; **65 (Supplement 1)**: S78.
12. Fitriana I, Setiati S, Rizal EW, et al. Malnutrition and depression as predictors for 30-day unplanned readmission in older patient: a prospective cohort study to develop 7-point scoring system. *BMC Geriatr* 2021; **21**(1): 256.
13. Laura T, Melvin C, Yoong DY. Depressive symptoms and malnutrition are associated with other geriatric syndromes and increase risk for 30-Day readmission in hospitalized older adults: a prospective cohort study. *BMC Geriatr* 2022; **22**(1): 634.
14. Anani S, Goldhaber G, Brom A, et al. Frailty and Sarcopenia Assessment upon HospitalAdmission to Internal Medicine Predicts Length ofHospital Stay and Re-Admission: A Prospective Study of 980 Patients. *J* 2020; **9**(8): 17.
15. Wallis SJ, Wall J, Biram RW, Romero-Ortuno R. Association of the clinical frailty scale with hospital outcomes. *Qjm* 2015; **108**(12): 943-9.
16. Subramaniam A, Tiruvoipati R, Green C, et al. Frailty status, timely goals of care documentation and clinical outcomes in older hospitalised medical patients. *Intern Med J* 2020; **06**: 06.
17. Street A, Maynou L, Gilbert T, Stone T, Mason S, Conroy S. The use of linked routine data to optimise calculation of the Hospital Frailty Risk Score on the basis of previous hospital admissions: a retrospective observational cohort study. *Lancet Healthy Longev* 2021; **2**(3): e154-e62.

18. Lujic S, Randall DA, Simpson JM, Falster MO, Jorm LR. Interaction effects of multimorbidity and frailty on adverse health outcomes in elderly hospitalised patients. *Sci Rep* 2022; **12**(1): 14139.
19. Gilbert T, Cordier Q, Polazzi S, et al. External validation of the Hospital Frailty Risk Score in France. *Age Ageing* 2022; **51**(1): afab126.
20. McAlister F, van Walraven C. External validation of the Hospital Frailty Risk Score and comparison with the Hospital-patient One-year Mortality Risk Score to predict outcomes in elderly hospitalised patients: a retrospective cohort study. *BMJ Qual Saf* 2019; **28**(4): 284-8.
21. Gilbert T, Neuburger J, Kraindler J, et al. Development and validation of a Hospital Frailty Risk Score focusing on older people in acute care settings using electronic hospital records: an observational study. *Lancet* 2018; **391**(10132): 1775-82.
22. Moola S MZ, Tufanaru C, Aromataris E, Sears K, Sfetcu R, Currie M, Lisy K, Qureshi R, Mattis P, Mu P. Chapter 7: Systematic reviews of etiology and risk.: JBI, 2020.
23. Munn Z MS, Lisy K, Riitano D, Tufanaru C. Chapter 5: Systematic reviews of prevalence and incidence: JBI, 2020.
24. Naing L WT, Rusli BN. Practical issues in calculating the sample size for prevalence studies. *Archives of Orofacial Sciences* 2006; **1**: 9-14.
25. Theou O, Squires E, Mallery K, et al. What do we know about frailty in the acute care setting? A scoping review. *BMC Geriatr* 2018; **18**(1): 139.
26. Inouye SK, Bogardus Jr ST, Vitagliano G, et al. Burden of illness score for elderly persons: risk adjustment incorporating the cumulative impact of diseases, physiologic abnormalities, and functional impairments. *Medical care* 2003; **41**(1): 70-83.
27. Clegg A, Bates C, Young J, et al. Development and validation of an electronic frailty index using routine primary care electronic health record data. *Age & Ageing* 2016; **45**(3): 353-60.
28. Bonizzoni E, Gussoni G, Agnelli G, et al. The complexity of patients hospitalized in Internal Medicine wards evaluated by FADOI-COMPLIMED score(s). A hypothetical approach. *PLoS One* 2018; **13**(4): e0195805.
29. Evans SJ, Sayers M, Mitnitski A, Rockwood K. The risk of adverse outcomes in hospitalized older patients in relation to a frailty index based on a comprehensive geriatric assessment. *Age Ageing* 2014; **43**(1): 127-32.
30. Morley JE, Malmstrom TK, Miller DK. A simple frailty questionnaire (FRAIL) predicts outcomes in middle aged African Americans. *J Nutr Health Aging* 2012; **16**(7): 601-8.
31. Hoogerduijn JG, Buurman BM, Korevaar JC, Grobbee DE, de Rooij SE, Schuurmans MJ. The prediction of functional decline in older hospitalised patients. *Age Ageing* 2012; **41**(3): 381-7.
32. Hirdes JP, Poss JW, Curtin-Telegdi N. The Method for Assigning Priority Levels (MAPLe): a new decision-support system for allocating home care resources. *BMC Med* 2008; **6**: 9.
33. Pilotto A, Ferrucci L, Franceschi M, et al. Development and validation of a multidimensional prognostic index for one-year mortality from comprehensive geriatric assessment in hospitalized older patients. *Rejuvenation Res* 2008; **11**(1): 151-61.
34. Hilmer SN, Perera V, Mitchell S, et al. The assessment of frailty in older people in acute care. *Australas J Ageing* 2009; **28**(4): 182-8.
35. Eeles EM, White SV, O'Mahony SM, Bayer AJ, Hubbard RE. The impact of frailty and delirium on mortality in older inpatients. *Age Ageing* 2012; **41**(3): 412-6.

36. Ensrud KE, Ewing SK, Taylor BC, et al. Comparison of 2 frailty indexes for prediction of falls, disability, fractures, and death in older women. *Arch Intern Med* 2008; **168**(4): 382-9.
37. Asmus-Szepesi KJ, de Vreede PL, Flinterman LE, et al. Prognosis of hospitalised older people with different levels of functioning: a prospective cohort study. *Age Ageing* 2013; **42**(6): 803-9.
38. Fujita K, Lo SY, Hubbard RE, Gnjidic D, Hilmer SN. Comparison of a multidomain frailty index from routine health data with the hospital frailty risk score in older patients in an Australian hospital. *Australas J Ageing* 2022.
39. Geriatric Medicine Research C. Delirium is prevalent in older hospital inpatients and associated with adverse outcomes: results of a prospective multi-centre study on World Delirium Awareness Day. *BMC Med* 2019; **17**(1): 229.
40. Hollinghurst J, Housley G, Watkins A, Clegg A, Gilbert T, Conroy SP. A comparison of two national frailty scoring systems. *Age Ageing* 2021; **50**(4): 1208-14.
41. Lim SH, Malhotra R, Ostbye T, et al. Sensitivity and specificity of three screening tools for frailty in hospitalized older adults. *Int J Nurs Stud* 2023; **139**: 104435.
42. Romero-Ortuno R, Wallis S, Biram R, Keevil V. Clinical frailty adds to acute illness severity in predicting mortality in hospitalized older adults: An observational study. *Eur J Intern Med* 2016; **35**: 24-34.
43. Romero-Ortuno R, Forsyth DR, Wilson KJ, et al. The Association of Geriatric Syndromes with Hospital Outcomes. *J Hosp Med* 2017; **12**(2): 83-9.
44. Soong J, Poots AJ, Scott S, et al. Quantifying the prevalence of frailty in English hospitals. *BMJ Open* 2015; **5**(10): e008456.
45. Soong JTY, Kaubryte J, Liew D, et al. Dr Foster global frailty score: an international retrospective observational study developing and validating a risk prediction model for hospitalised older persons from administrative data sets. *BMJ Open* 2019; **9**(6): e026759.
46. Timmons S, Manning E, Barrett A, et al. Dementia in older people admitted to hospital: a regional multi-hospital observational study of prevalence, associations and case recognition. *Age Ageing* 2015; **44**(6): 993-9.
47. Warnier RMJ, van Rossum E, van Kuijk SMJ, Magdelijns F, Schols J, Kempen G. Frailty screening in hospitalised older adults: How does the brief Dutch National Safety Management Program perform compared to a more extensive approach? *J Clin Nurs* 2020; **29**(7-8): 1064-73.
48. Belga S, Majumdar SR, Kahlon S, et al. Comparing three different measures of frailty in medical inpatients: Multicenter prospective cohort study examining 30-day risk of readmission or death. *J Hosp Med* 2016; **11**(8): 556-62.
49. Kahlon S, Pederson J, Majumdar SR, et al. Association between frailty and 30-day outcomes after discharge from hospital. *CMAJ* 2015; **187**(11): 799-804.
50. McAlister FA, Lin M, Bakal JA. Prevalence and Postdischarge Outcomes Associated with Frailty in Medical Inpatients: Impact of Different Frailty Definitions. *J Hosp Med* 2019; **14**(7): 407-10.
51. Buurman BM, Hoogerduijn JG, van Gemert EA, de Haan RJ, Schuurmans MJ, de Rooij SE. Clinical characteristics and outcomes of hospitalized older patients with distinct risk profiles for functional decline: a prospective cohort study. *PLoS ONE* 2012; **7**(1): e29621.

52. Dani M, Owen LH, Jackson TA, Rockwood K, Sampson EL, Davis D. Delirium, Frailty, and Mortality: Interactions in a Prospective Study of Hospitalized Older People. *J Gerontol A Biol Sci Med Sci* 2018; **73**(3): 415-8.
53. El-Sharkawy AM, Watson P, Neal KR, et al. Hydration and outcome in older patients admitted to hospital (The HOOP prospective cohort study). *Age Ageing* 2015; **44**(6): 943-7.
54. Gregorevic KJ, Hubbard RE, Lim WK, Katz B. The clinical frailty scale predicts functional decline and mortality when used by junior medical staff: a prospective cohort study. *BMC Geriatr* 2016; **16**: 117.
55. Hernandez-Luis R, Martin-Ponce E, Monereo-Munoz M, et al. Prognostic value of physical function tests and muscle mass in elderly hospitalized patients. A prospective observational study. *Geriatr Gerontol Int* 2018; **18**(1): 57-64.
56. Irina G, Refaela C, Adi B, et al. Low Blood ALT Activity and High FRAIL Questionnaire Scores Correlate with Increased Mortality and with Each Other. A Prospective Study in the Internal Medicine Department. *J* 2018; **7**(11): 25.
57. Juma S, Taabazuing M-M, Montero-Odasso M. Clinical Frailty Scale in an Acute Medicine Unit: a Simple Tool That Predicts Length of Stay. *Can Geriatr J* 2016; **19**(2): 34-9.
58. Khandelwal D, Goel A, Kumar U, Gulati V, Narang R, Dey AB. Frailty is associated with longer hospital stay and increased mortality in hospitalized older patients. *J Nutr Health Aging* 2012; **16**(8): 732-5.
59. McCrow J, Morton M, Travers C, Harvey K, Eeles E. Associations Between Dehydration, Cognitive Impairment, and Frailty in Older Hospitalized Patients. *J Gerontol Nurs* 2016; **42**(5): 19-27.
60. Nardi R, Nozzoli C, Berti F, et al. Prognostic value for mortality of the new FADOI-COMPLIMED score(s) in patients hospitalized in medical wards. *PLoS ONE* 2019; **14**(7): e0219767.
61. Noro A, Poss JW, Hirdes JP, et al. Method for Assigning Priority Levels in Acute Care (MAPLe-AC) predicts outcomes of acute hospital care of older persons--a cross-national validation. *BMC Med Inf Decis Mak* 2011; **11**: 39.
62. Polidoro A, Stefanelli F, Ciacciarelli M, Pacelli A, Di Sanzo D, Alessandri C. Frailty in patients affected by atrial fibrillation. *Arch Gerontol Geriatr* 2013; **57**(3): 325-7.
63. Ramdass SK, Brennan MJ, Starr R, et al. The association of frailty with discharge disposition for hospitalized community dwelling elderly patients. *J Hosp Med* 2018; **13**(3): 182-4.
64. Rizza S, Morabito P, De Meo L, et al. IL-6 levels influence 3-month all-cause mortality in frail hospitalized older patients. *Aging and Disease* 2021; **12**(2): 353-9.
65. Rose M, Pan H, Levinson MR, Staples M. Can frailty predict complicated care needs and length of stay? *Intern Med J* 2014; **44**(8): 800-5.
66. Sharma Y, Avina P, Ross E, Horwood C, Hakendorf P, Thompson C. Validity of the Malnutrition Universal Screening Tool for Evaluation of Frailty Status in Older Hospitalised Patients. *Gerontol Geriatr Med* 2022; **8**.
67. Oud FMM, De Rooij SEJA, Schuurman T, Duijvelaar KM, Van Munster BC. Predictive value of the VMS theme 'Frail elderly': Delirium, falling and mortality in elderly hospital patients. [Dutch]. *Ned Tijdschr Geneeskd* 2015; **159**(14).

68. Heim N, van Fenema EM, Weverling-Rijnsburger AW, et al. Optimal screening for increased risk for adverse outcomes in hospitalised older adults. *Age Ageing* 2015; **44**(2): 239-44.
69. Lo SY, Zhang M, Hubbard RE, Gnjidic D, Redston MR, Hilmer SN. Development and validation of a frailty index based on data routinely collected across multiple domains in NSW hospitals. *Australas J Ageing* 2021; **40**(2): 184-94.
70. Romero-Ortuno R, Walsh CD, Lawlor BA, Kenny RA. A frailty instrument for primary care: findings from the Survey of Health, Ageing and Retirement in Europe (SHARE). *BMC Geriatr* 2010; **10**: 57.
71. Munn Z, Moola S, Lisy K, Riitano D, Tufanaru C. Methodological guidance for systematic reviews of observational epidemiological studies reporting prevalence and cumulative incidence data. *Int J Evid Based Healthc* 2015; **13**(3): 147-53.
